# The tomato WRKY-B transcription factor modulates lateral branching by targeting *BLIND*, *PIN4*, and *IAA15*

**DOI:** 10.1093/hr/uhae193

**Published:** 2024-07-11

**Authors:** Huanhuan Yang, Ke Zhou, Qingfei Wu, Xinyi Jia, Hexuan Wang, Wenhui Yang, Lihao Lin, Xiaomeng Hu, Bingqing Pan, Ping Li, Tingting Huang, Xiangyang Xu, Jingfu Li, Jingbin Jiang, Minmin Du

**Affiliations:** College of Horticulture and Landscape Architecture, Northeast Agricultural University, Harbin 150030, China; College of Horticulture, China Agricultural University, Beijing 100083, China; College of Life Sciences, Yan’an University, Yan’an 716000, China; College of Horticulture and Landscape Architecture, Northeast Agricultural University, Harbin 150030, China; College of Horticulture and Landscape Architecture, Northeast Agricultural University, Harbin 150030, China; College of Horticulture and Landscape Architecture, Northeast Agricultural University, Harbin 150030, China; College of Agriculture, Ningxia Universisty, Yinchuan 750002, China; College of Horticulture, China Agricultural University, Beijing 100083, China; College of Horticulture, China Agricultural University, Beijing 100083, China; Qingdao Academy of Agricultural Sciences, Qingdao City 266000, China; Qingdao Academy of Agricultural Sciences, Qingdao City 266000, China; College of Horticulture and Landscape Architecture, Northeast Agricultural University, Harbin 150030, China; College of Horticulture and Landscape Architecture, Northeast Agricultural University, Harbin 150030, China; College of Horticulture and Landscape Architecture, Northeast Agricultural University, Harbin 150030, China; College of Horticulture, China Agricultural University, Beijing 100083, China

## Abstract

Lateral branching is a crucial agronomic trait that impacts crop yield. In tomato (***Solanum lycopersicum***), excessive lateral branching is unfavorable and results in substantial labor and management costs. Therefore, optimizing lateral branching is a primary objective in tomato breeding. Although many genes related to lateral branching have been reported in tomato, the molecular mechanism underlying their network remains elusive. In this study, we found that the expression profile of a *WRKY* gene, *WRKY-B* (for *WRKY-BRANCING*), was associated with the auxin-dependent axillary bud development process. *Wrky-b* mutants generated by the CRISPR/Cas9 editing system presented fewer lateral branches, while *WRKY-B* overexpression lines presented more lateral branches than did wild-type plants. Furthermore, WRKY-B can directly target the well-known branching gene *BLIND* (*BL*) and the auxin efflux carrier gene *PIN4* to activate their expression. Both the *bl* and *pin4* mutants exhibited reduced lateral branching, similar to the *wrky-b* mutant. The IAA contents in the axillary buds of the *wrky-b*, *bl*, and *pin4* mutant plants were significantly higher than those in the wild-type plants. In addition, WRKY-B can also directly target the AUX/IAA gene *IAA15* and repress its expression. In summary, *WRKY-B* works upstream of *BL*, *PIN4*, and *IAA15* to regulate the development of lateral branches in tomato.

## Introduction

Plant architecture plays a crucial role in shaping plant morphology and influencing agricultural productivity. It is determined by factors such as plant height, branching patterns, leaf morphology, and panicle structure [1]. Developing an optimal plant architecture is vital for enhancing crop yield through progressive crop domestication [2]. During the process of crop domestication, characteristics like lateral branching were favored through selective breeding [3]. Domesticated crop plants often exhibit reduced axillary branching numbers and angles compared to their wild counterparts, making them more suitable for dense cultivation and higher yields [4, 5]. For instance, the evolution of cultivated maize from wild teosinte involved a shift from a multibranched to a branchless architecture, largely due to a mutation in the *TB1* (*Teosinte Branched 1*) gene [6, 7]. Similarly, cultivated rice plants are characterized by fewer tillers due to long-term artificial selection and domestication, while wild rice plants exhibit a sprawling growth pattern with multiple tillers. *OsTB2* and *PROSTRATE GROWTH 1* (*PROG1*) were artificially selected during rice domestication [8]. Therefore, further elucidation of the specific molecular mechanisms regulating branching is highly important for understanding plant domestication and for improving crop genetics.

The development of lateral branches in plants is controlled by an intricate regulatory network consisting of two main processes: the initiation of axillary meristem (AM) and the outgrowth of the axillary bud [9, 10]. Over the years, researchers have discovered many essential genes that play a role in regulating lateral branch development in various plant species including rice, *Arabidopsis*, peas, *Camellia sinensis* and other plants [11–15]. These genes can be grouped into three categories according to their influence on the stages of lateral branch development. The first category encompasses genes associated with AM formation, including *LATERAL SUPPRESSOR* (*LAS*) and *Lax Panicle 1* (*LAX1*) in rice, as well as *Regulator of Axillary Meristems 1* (*RAX1*) in *Arabidopsis*. *LAS* is a member of the GRAS transcription factor family [16]. The lack or disruption of *LAS*, along with its orthologous genes such as lateral suppressor (*LS*) in tomato and *MONOCULM1* (*MOC1*) in rice, leads to the absence of AM, branches, or tillers. This finding indicated a high level of conservation in the gene functionality [17, 18]. In rice, the *LAX1* gene encodes a bHLH transcription factor, and its mutation results in a decrease in panicle and tiller numbers [19]. The *RAX1* gene, which regulates AM formation in *Arabidopsis*, is homologous to *BLIND* (*BL*) in tomato and is part of the R2R3 subclass of the MYB gene family [20, 21]. The second category includes genes implicated in axillary bud outgrowth, such as *More Axillary Growth* (*MAX*) and *TERMINAL FLOWER 1* (*TFL1*). *MAX* genes play a crucial role in the biosynthesis and signal transduction of strigolactones, which are key regulators of lateral bud growth in *Arabidopsis* [22]. On the other hand, *TFL1*, a gene encoding a homologous protein to the phosphatidylethanolamine binding protein (PEBP), has been identified as the master regulator of lateral bud activation in *Arabidopsis* [23]. The third category includes genes that affect both the formation and outgrowth of lateral buds, such as *Supershoot* (*SPS*), *Bushy* (*BUS*), and *TB1*. *SPS* and *BUS* belong to the cytochrome P450 family, with the ability to regulate lateral branching through influencing AM initiation and growth [24, 25]. The function of *TB1* in maize, rice and *Arabidopsis*, as described above, is conserved, and all of these genes are involved in AM formation and lateral outgrowth [26].

Hormones are also essential for the development of lateral branches and buds. Studies have demonstrated that hormone levels regulate the dormancy of lateral buds. Auxin (indole-3-acetic acid, IAA) plays a role in inhibiting lateral bud growth by sustaining apical dominance, while cytokinins are involved in promoting lateral bud growth [27, 28]. Gibberellins (GAs) and brassinosteroids (BRs) have contrasting effects on lateral bud growth, with GAs inhibiting and BRs promoting their development [29, 30]. Furthermore, strigolactones (SLs) function as signaling molecules that operate over long distances to suppress the development of branches. These molecules interfere with the polar transport of IAA and can also trigger the expression of *TB1*/*BRC1* genes, which in turn inhibits the growth of lateral buds [31–33]. In cucumber, *CsBRC1* promotes the accumulation of auxin in axillary buds by directly inhibiting *CsPIN3* function, thereby inhibiting the growth of lateral buds [34].

Excessive branching in various horticultural crops like tomato (*Solanum lycopersicum*) can result in nutrient and light competition, ultimately decreasing crop yield. To enhance both yield and quality, it is necessary to manually eliminate additional branches in tomato cultivation, particularly for fresh market tomatoes. To date, many genes regulating lateral branch development have been reported in tomato. For instance, two *BRC1* paralogs identified in tomato, named *SlBRC1a* and *SlBRC1b*, exhibit specific expression patterns in axillary buds. Notably, only *SIBRC1b* is crucial for the outgrowth of lateral buds, whereas *SIBRC1a* is not [35]. The *LS* and *BL* genes are involved in lateral branch development by affecting AM initiation [36]. Meanwhile, miR156a has been found to target seven *SPL* genes, impacting fruit yield and the formation of lateral branches in tomato [37]. The transcription factor *SlTCP26* influences auxin and abscisic acid pathways to diminish apical dominance and activate lateral bud dormancy, thereby enhancing the growth of lateral branches [38]. Mutagenesis of the DNA methyltransferase gene *SlCMT4* causes an increase in lateral branches [39].

Herein, expression pattern analysis revealed that a *WRKY* gene (*Solyc02g071130*) was strongly associated with the auxin-dependent axillary bud development process. *Wrky-b* mutants exhibited reduced lateral branches, while the *WRKY-B* overexpression lines produced many more lateral branches. Furthermore, by combining chromatin immunoprecipitation sequencing (ChIP-Seq), transcriptome sequencing (RNA-Seq), and biochemical analyses, we found that WRKY-B can directly bind the promoters of *BL*, *PIN4*, and *IAA15*, thus regulating AM initiation and lateral bud outgrowth.

## Results

### WRKY-B is an auxin-induced transcription factor

**Figure 1:**
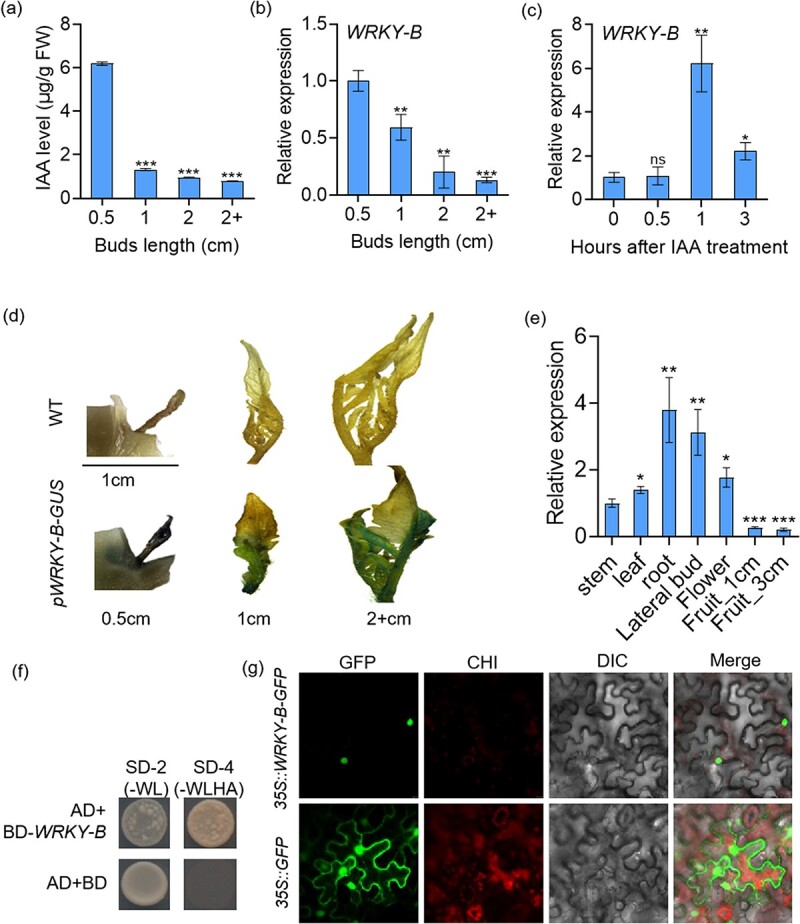
*WRKY-B* is associated with the auxin-dependent axillary bud development process. **(a)** The content of endogenous IAA in the indicated length of axillary buds at the second node of wild-type tomato plants 45 d after sowing in the greenhouse. **(b)** Expression levels of *WRKY-B* in the indicated length of axillary buds at the second node of wild-type tomato plants 45 d after sowing. **(c)** Time course of *WRKY-B* expression in wild-type tomato seedlings treated with 50 μM IAA. **(d)** GUS staining of the indicated length of axillary buds at the second node in *ProWRKY-B::GUS* transgenic plants. The indicated length of axillary buds were harvested at the same node from three independent plants. **(e)** Expression levels of *WRKY-B* in different tomato tissues. **(f)** Transcriptional activation activity of WRKY-B in yeast. **(g)** Subcellular localization of WRKY-B-GFP in *Nicotiana benthamiana* leaves. Error bars represent the SD of three biological replicates. Asterisks indicate significant differences according to Student’s *t*-test (ns, no significant, ^*^P < 0.05, ^**^P < 0.01, ^***^P < 0.001)

We measured IAA (indole-3-acetic acid) levels at various developmental stages in the axillary buds. The results showed that the endogenous IAA content decreased significantly with the early elongation of axillary buds. The content of IAA was found to be highest in the 0.5 cm long axillary buds, which then decreased sharply in the 1 cm long axillary buds. The IAA content further decreased in the 2 cm long auxiliary buds, but the latter decrease was smaller (Fig. 1a). *WRKY-B* (*Solyc02g071130*) was found to have an expression pattern corelated to the change in IAA content during axillary bud development, with a gradual decrease in expression level with increasing axillary bud growth (Fig. 1b). Furthermore, transcription levels of *WRKY-B* were significantly induced by exogenous auxin (Fig. 1c), suggesting that *WRKY-B* may be involved in auxin-dependent axillary bud development. Sequence analysis revealed that WRKY-B encodes a protein of 317 amino acids, and phylogenetic analysis revealed that *WRKY-B* clustered closely with *AtWRKY28*, *AtWRKY8*, and *AtWRKY71* (Fig. S1, see online supplementary material). Transgenic tomato plants harboring *ProWRKY-B*::*GUS* were also constructed to confirm the expression pattern in the axillary buds. Consistent with the change in IAA content during axillary bud development, GUS staining showed that the expression of WRKY-B was highest in the 0.5 cm long axillary buds and gradually decreased as the buds grew (Fig. 1d), indicating that *WRKY-B* expression was higher in the younger axillary buds. To achieve a comprehensive spatiotemporal expression profile of *WRKY-B*, the expression levels of the *WRKY-B* gene were evaluated using qRT-PCR. *WRKY-B* was ubiquitously expressed in various tissues, with the highest expression in roots followed by axillary buds (Fig. 1e). Furthermore, transcriptional activity assay and subcellular localization experiment showed that WRKY-B had the activation activity (Fig. 1f) and was exclusively localized to the nucleus (Fig. 1g), consistent with the defining features of a typical transcription factor.

### WRKY-B regulates the initiation and outgrowth of lateral branches

To understand the role of *WRKY-B* in tomato, the CRISPR/Cas9 system was used to generate *wrky-b* mutants. Two distinct target sites within the first exon region of *WRKY-B* were identified and incorporated into the CRISPR/Cas9 vector (Fig. S2a, see online supplementary material). This resulted in the creation of 12 mutant lines, each exhibiting various mutation types. Sequencing analysis showed that the *wrky-b-c1* line had a single base deletion at the first target site, leading to a frameshift in the open reading frame accompanied by premature translation termination. Similarly, the *wrky-b-c2* line had a one-base deletion at the first target site and two-base deletions at the second target site, causing a frameshift in the open reading frame and premature translation termination as well (Fig. S2a, see online supplementary material). Moreover, *WRKY-B* expression was barely detectable in these two mutants (Fig. S2b, see online supplementary material), indicating that these mutants were loss-of-function mutants. We subsequently selected these two independent mutant lines for further study.

**Figure 2:**
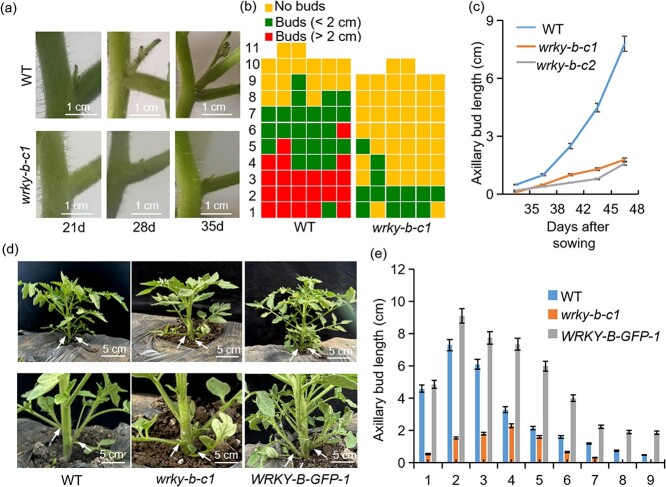
WRKY-B promotes axillary buds development. **(a)** Phenotypes of axillary buds at the second node of WT and *wrky-b* mutants. 21d, 28d, and 35d, respectively represent indicated days after sowing. **(b)** Diagrammatic data showed the development of axillary buds in different nodes in WT and *wrky-b* mutants 45 d after sowing. Each row represents a node in tomato. Each column represents an individual plant of WT or *wrky-b* lines. **(c)** The axillary bud length at the sixth node was measured at the indicated time. Each dot indicates the mean value of the axillary bud length of six individual plants at the sixth node at the indicated time. **(d)** Representative images of WT, *wrky-b* mutants and *WRKY-B-GFP* overexpressing transgenic lines 45 d after sowing. The arrows indicate an axillary bud. Scale bars represent 5 cm. **(e)** The length of each axillary bud in WT, *wrky-b* mutants and *WRKY-B-GFP* overexpressing transgenic lines 45 d after sowing from the first to ninth nodes. Error bars represent the SD of three biological replicates in (c and e). Scale bars represent 1 cm in (a), and 5 cm in (d).

The WT plants began to produce axillary buds 21 d after sowing, while the *wrky-b-c1* mutant began to produce axillary buds 35 d after sowing (Fig. 2a), indicating a slower initiation of axillary bud formation in the *wrky-b* mutant than in WT plants. Quantitative analysis of the branching pattern revealed that 45 d after sowing, the *wrky-b* plants formed only one to three axillary buds that were less than 2 cm in length on the examined nodes, while the WT plants produced seven to nine axillary buds, most of which were greater than 2 cm in length (Fig. 2b). Furthermore, time-course analysis conducted from 35 to 48 d after sowing revealed that the outgrowth rate of axillary buds in the mutant was much slower than that in WT plants, resulting in a shorter axillary bud length in the mutant than in the WT (Fig. 2c). In addition, we generated *35S::WRKY-B-GFP* (*WRKY-B-GFP*) plants in which the *WRKY-B* expression level was significantly greater than that in the WT (Fig. S2b, see online supplementary material). *WRKY-B-GFP* transgenic lines produced more lateral branches than WT plants, while the *wrky-b* mutants exhibited fewer lateral branches (Fig. 2d). Similarly, at 45 d after sowing, the maximum length of lateral branches in WT plants was approximately 7.0 cm, whereas it was around 9 cm for *WRKY-B-GFP* transgenic lines and only 2.5 cm for *wrky-b* mutants (Fig. 2e). Taken together, these data suggested that WRKY-B plays a positive role in regulating the initiation and outgrowth of lateral branches in tomato.

### WRKY-B regulates the expression of genes involved in lateral branching and auxin-related pathway

**Figure 3:**
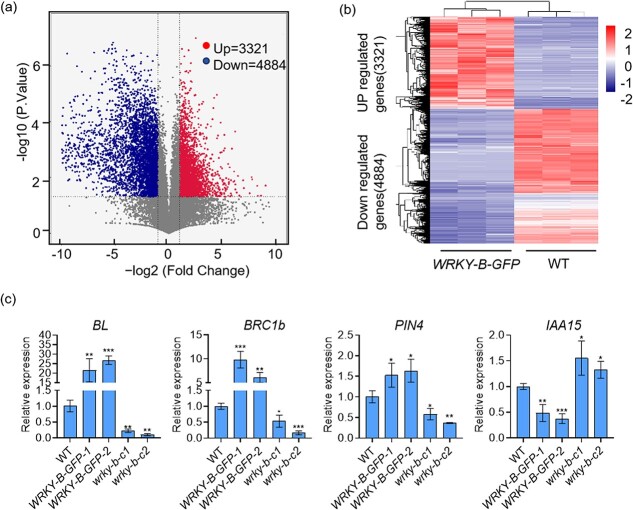
Transcriptome analysis of WRKY-B-regulated genes. **(a)** Volcano plots showing the number of differentially expressed genes (DEGs) regulated by WRKY-B. **(b)** Hierarchical clustering and heatmap analysis of the WRKY-B-regulated DEGs. **(c)** Relative expression of WRKY-B-regulated genes in WT, *wrky-b*, and *WRKY-B-GFP* plants. Error bars represent the SD of three biological replicates. Asterisks indicate significant differences according to Student’s *t*-test (*P < 0.05, **P < 0.01, ***P < 0.001).

Transcriptome analysis was conducted on WT plants and *WRKY-B-GFP* transgenic plants to investigate the role of *WRKY-B* in regulating the development of axillary buds. The RNA-Seq analysis involved extracting total RNA from axillary buds of both types of plants at the sixth nodes during the 45-d-old seedling stage. Each sample included three biological replicates, resulting in the creation of a total of six libraries for sequencing. Differentially expressed genes (DEGs) were identified between the WT and *WRKY-B-GFP* groups using the criteria of a fold change ≥2 and an FDR-adjusted *P* value <0.05. In total, 8205 DEGs were identified. There were 3321 significantly upregulated genes and 4884 downregulated genes in the *WRKY-B-GFP* transgenic plants (Fig. 3a and b). Heatmap analysis revealed that 16 auxin response factors (ARFs) and 18 Aux/IAA proteins were significantly differentially expressed in the *WRKY-B-GFP* plants (Fig. S3a, see online supplementary material). A total of seven auxin efflux carrier genes were differentially expressed according to RNA-Seq; among them, *PIN3*, *PIN4*, *PIN1*, and *PIN9* were significantly upregulated in the *WRKY-B-GFP* plants, while *PIN5*, *PIN8*, and *PIN7* were significantly downregulated. Moreover, five auxin influx carriers, *LAX1*, *LAX2*, *LAX3*, *LAX4*, and *LAX5*, were differentially expressed; only *LAX3* was significantly upregulated in the *WRKY-B-GFP* plants, while the other four were significantly downregulated (Fig. S3b, see online supplementary material). Notably, several well-known genes associated with lateral branch development were found among these DEGs. For instance, *RAX2*, *BL*, *BZR1*, *SPL13*, *BRC1b*, and *CKK2* were significantly upregulated in *WRKY-B-GFP* plants, while *D14*, *ABCB19*, *SIGOB*, and *LOG1* were significantly downregulated in *WRKY-B-GFP* plants (Fig. S3c, see online supplementary material). The expression patterns of several selected genes, including *BL*, *BRC1b*, *PIN4*, and *IAA15*, were analysed via qRT–PCR (Fig. 3c). These results were mostly consistent with the trends observed in the above RNA-seq experiments.

### Genome-wide binding profiles of WRKY-B in tomato

**Figure 4:**
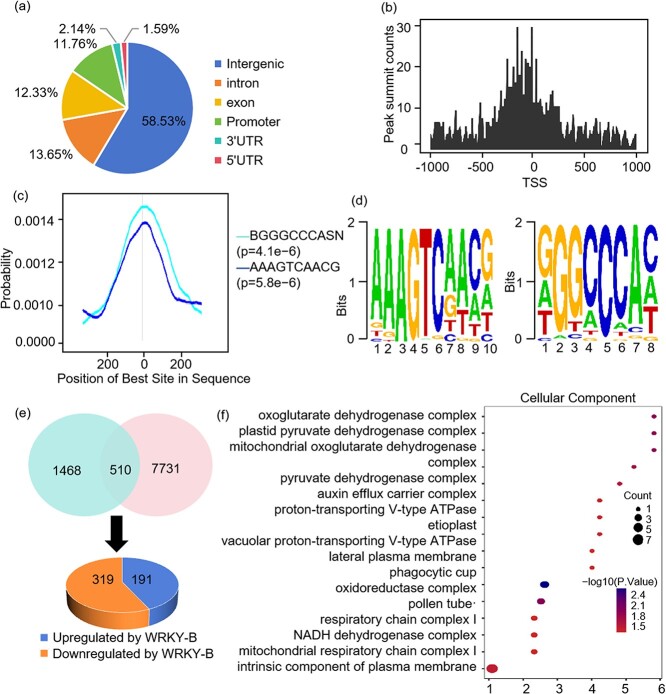
Genome-wide identification of WRKY-B binding sites and motifs. **(a)** Genome-wide distribution analysis of the overlapping WRKY-B binding peaks. **(b)** WRKY-B binding peaks are highly enriched in the 200-bp region immediately upstream of the TSS. The overlapping peaks were used for analysis. **(c)** Binding motifs identified in the overlapping WRKY-B binding peaks. DREME motif search identified two WRKY-B binding motifs (BGGGCCCASN and AAAGTCAACG). Density plots illustrate the dispersion of various WRKY-B binding patterns encompassing the summits of overlapping peaks. The significance level, represented by the *P*-value from a one-tailed binomial test, is enclosed in parentheses. **(d)** Binding motifs identified in the overlapping WRKY-B binding peaks. DREME motif search identified the WRKY-B binding motifs (BGGGCCCASN and AAAGTCAACG). **(e)** Venn diagram showing the overlap of WRKY-B-targeted genes (1978, from ChIP-Seq analysis) and WRKY-B-regulated genes (8241, from RNA-Seq analysis). The genes located in the overlapping region were identified as targets of WRKY-B and were further classified into sets of either upregulated or downregulated genes according to RNA-Seq analysis. **(f)** GO analysis of WRKY-B-targeted genes.

Chromatin immunoprecipitation sequencing (ChIP-Seq) analysis was conducted on axillary buds of 45-day-old *WRKY-B-GFP* plants to pinpoint *WRKY-B* binding sites across the genome. The analysis demonstrated that *WRKY-B* binding peaks were notably present in various genomic regions such as introns, exons, promoters, 3’ UTRs, and intergenic regions (Fig. 4a). A significant portion (11.76%) of *WRKY-B* binding peaks were concentrated in promoter regions located 3 kb upstream of the transcription start site (TSS). Further investigation into the WRKY-B binding profile in the promoter region revealed a high enrichment of binding sites in the proximal promoter region, with a peak occurring approximately 200 bp upstream of the TSSs (Fig. 4b). In order to uncover the binding motifs of WRKY-B, de novo motif prediction was carried out through the utilization of Multiple Em for Motif Elimination (MEME) software, utilizing the WRKY-B binding peaks detected via ChIP-Seq analysis. This examination led to the discovery of two distinct motifs: BGGGCCCASN and AAAGTCAACG (Fig. 4c and d).

Up to 2243 overlapping peaks were detected from different biological replicates in our ChIP-Seq. These overlapping peaks were assigned to the closest genes, and a total of 1978 genes were identified as potential WRKY-B-bound genes (Data Set SS1, see online supplementary material). RNA-Seq data revealed that 8205 genes were regulated by WRKY-B. Combining the ChIP-Seq and RNA-Seq data, 510 overlapping genes were identified as WRKY-B-targeted genes, which are bound and regulated by WRKY-B (Fig. 4e). Among these genes, 191 (37.5%) were upregulated by WRKY-B, while 319 (72.5%) were downregulated by WRKY-B (Fig. 4e). The 510 overlapping genes were further classified and characterized based on the functional terms annotated in the Gene Ontology (GO) database. GO enrichment analysis demonstrated that these genes were significantly enriched in terms such as the auxin efflux carrier complex, proton-transport V-type ATPase, and vacuolar proton-transport V-type ATPase (Fig. 4f).

### WRKY-B directly activates the expression of *BL* during the development of lateral branches

**Figure 5:**
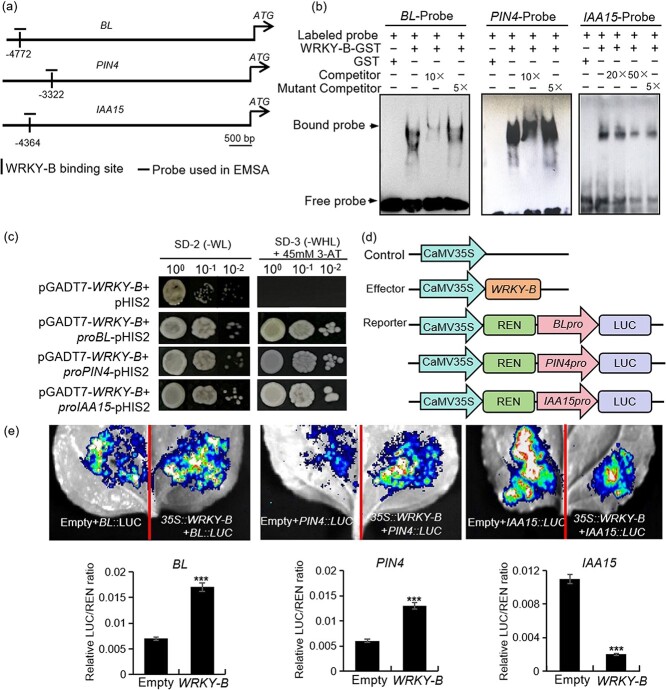
WRKY-B directly regulates gene expressions of *BL*, *PIN4*, and *IAA15.*  **(a)** Schematic diagram of the promoters of the indicated genes. The vertical line indicates WRKY-B binding site, and horizontal line indicates probe used in EMSA. **(b)** EMSA showing that WRKY-B-GST recombinant protein directly binds to the putative TTGAC-box in the promoters of the indicated gene. GST recombinant proteins without WRKY-B were used as negative controls. **(c)** Y1H assays showing that WRKY-B could bind to the promoter of the indicated gene. The *BL* promoter sequence from −4600 to −4999 contains the one TTGAC motif fused to the HIS2 reporter gene. The *PIN4* promoter sequence from −3200 to −3519 contains the one TTGAC motif fused to the HIS2 reporter gene. The *IAA15* promoter sequence from −4260 to −4439 contains the one TTGAC motif fused to the HIS2 reporter gene. pHIS2, empty vector, was used as the negative control; pGADT7-WRKY-B, prey vector containing *WRKY-B.*  **(d)** Schematics showing effector constructs for WRKY-B and reporter constructs with BL, PIN4, and IAA15 driving the firefly luciferase (LUC) gene for transient infiltration experiments. **(e)** Transient dual-luciferase expression assay showing the trans-activation of *BL*, *PIN4*, and *IAA15* by WRKY-B in *Nicotiana benthamiana* leaves. p35S empty vectors were used as the negative control. Bottom shows means ± SD from three biological replicates. Asterisks indicate significant differences according to Student’s *t*-test (***P < 0.001).

In order to provide further insight into the regulatory mechanism of WRKY-B, our study delved into its modulation of downstream gene expression. The tomato *BL* gene, a member of the MYB transcription factor gene family, plays a crucial role in controlling lateral meristem initiation to influence lateral branch development. Our analysis of RNA-Seq and ChIP-Seq data unveiled the *BL* gene as a target of WRKY-B (Data Set SS1 and Fig. S4, see online supplementary material). Subsequent qRT-PCR showed that the expression of *BL* was notably downregulated in *wrky-b* mutants compared to WT, while it was significantly upregulated in *WRKY-B-GFP* transgenic plants. To investigate the binding capability of WRKY-B to the *BL* promoter, electrophoretic mobility shift assays (EMSAs) were conducted using DNA probes derived from the *BL* promoter containing the AAAACTGAAA motif alongside corresponding mutant probes (Fig. 5a; Fig. S4b, see online supplementary material). Our findings indicated direct binding of WRKY-B-GST to the labeled DNA probes containing the mentioned motif, but not to a mutant probe lacking it (Fig. 5b). Furthermore, yeast one-hybrid experiments confirmed the interaction between WRKY-B and the *BL* promoter (Fig. 5c). In a transient transcription dual-luciferase reporter assay in tobacco leaves (Fig. 5d), the activity of LUC derived from *BL* promoters exhibited a notable increase upon co-transfection with WRKY-B (Fig. 5e), signifying direct activation of *BL* expression by WRKY-B.

**Figure 6:**
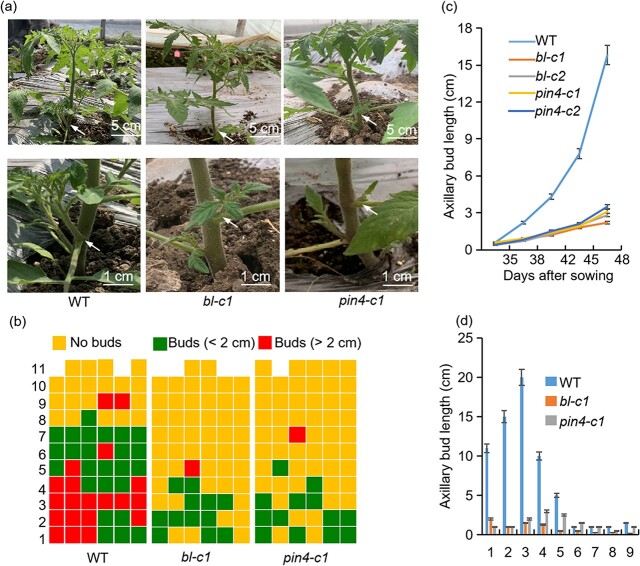
*BL* and *PIN4* positively regulate axillary buds development. **(a)** Representative images of WT, *bl*, and *pin4* plants 45 d after sowing. **(b)** Diagrammatic data showed the development of axillary buds in different nodes in WT, *bl*, and *pin4* plants 45 d after sowing. Each row represents a node in tomato. Each column represents an individual plant of indicated plants. **(c)** The axillary bud length at the sixth nodes of WT, *bl*, and *pin4* plants was measured at the indicated time. Each dot indicates the mean value of the axillary bud length of six individual plants at the sixth nodes at the indicated time. **(d)** The length of each axillary bud from node 1 to node 9 in WT, *bl*, and *pin4* plants 45 d after sowing. This value is the average of the axillary bud lengths of three independent plants on the same node. Error bars represent the SD of three biological replicates.

To delve deeper into the role of *BL* in the development of tomato lateral branches, we created *bl* mutants (*bl-c*) using the CRISPR/Cas9 system. Following genetic transformation and sequencing, we obtained two distinct homozygous *bl* mutants. In these mutants, *bl-c1* exhibited a single base deletion at the initial target site, while *bl-c2* demonstrated two base deletions at the second target site (Fig. S5, see online supplementary material). Both mutations resulted in premature termination of translation, underscoring that these mutants were loss-of-function types. Similar to *wrky-b* mutants, *bl* mutants displayed fewer lateral branches, with their lengths being notably shorter than those observed in the WT (Fig. 6a). A quantitative examination of the branching pattern across 11 assessed nodes indicated that *bl* mutants generated two to four axillary buds, most of which were under 2 cm in length, with only a minority exceeding 2 cm (Fig. 6b). Additionally, a time-course study from 35 to 48 d post-sowing showed that the axillary bud outgrowth percentage was significantly reduced in *bl* mutants compared to WT plants (Fig. 6c). Data quantification showed that at 45 d after sowing, the length of the lateral branch in the WT was approximately 10–20 cm, while the length of the lateral branches in the *bl* mutants was mostly no longer than 2 cm (Fig. 6d). Furthermore, both *wrky-b* and *bl* mutants demonstrated determinate growth habits, with shoot growth ceasing after the development of seven to eight inflorescences, each followed by two consecutive inflorescences (Fig. S6, see online supplementary material). This phenotype was previously reported in the classical *bl* mutant [37]. These findings indicated that *BL* is a direct transcriptional target of WRKY-B during lateral branch development.

### WRKY-B targets *PIN4* and *IAA15* in the auxin signaling pathway

**Figure 7:**
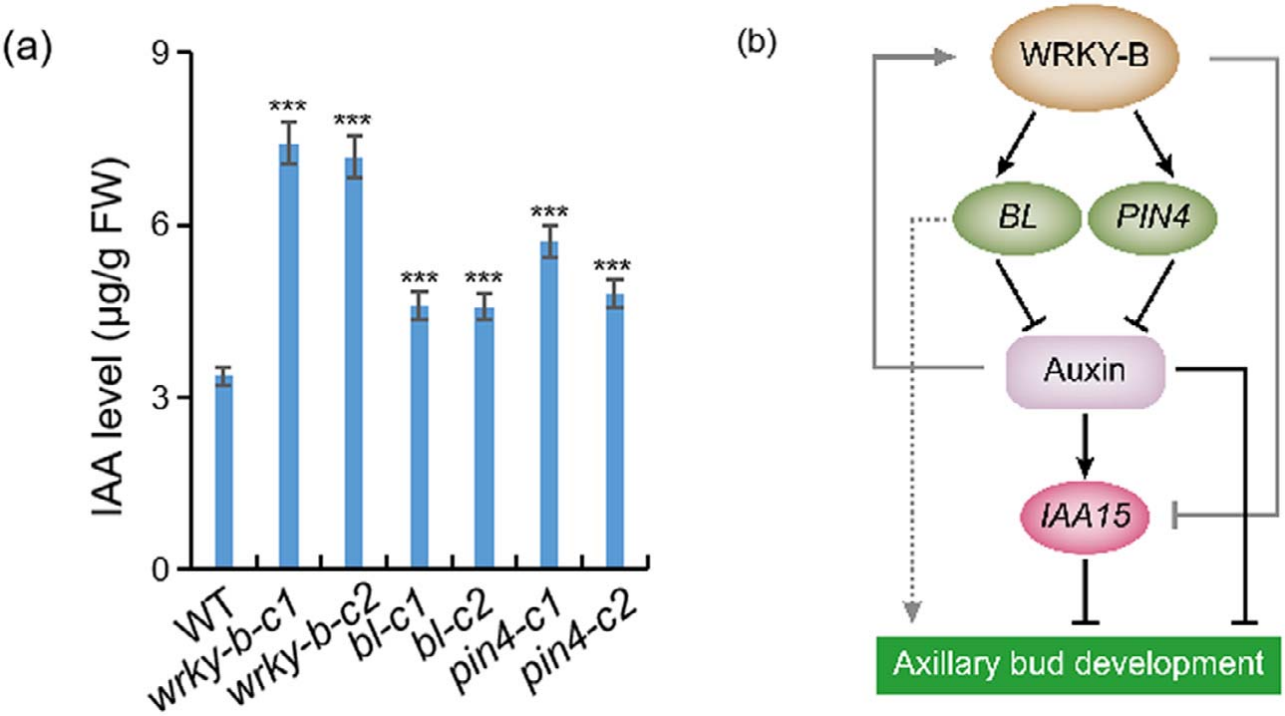
A working model of WRKY-B in promoting lateral shooting in tomato. **(a)** The content of endogenous IAA of axillary buds in WT, *wrky-b*, *bl*, and *pin4* mutants. The sixth node axillary buds were collected to measure the concentration of IAA from different plants 45 d after field sowing. Error bars represent the SD of three biological replicates. Asterisks indicate significant differences according to Student’s *t*-test (***P < 0.001). **(b)** Working model of WRKY-B regulated axillary buds development.

We found that *WRKY-B* expression might be associated with the development of axillary buds in an auxin-dependent manner (Fig. 1a–c). Furthermore, GO enrichment analysis demonstrated that the target genes of WRKY-B were also present in the auxin efflux carrier complex (Fig. 4f). This observation implies that WRKY-B could influence auxin signaling pathways to govern lateral branching. Notably, the gene *PIN4*, which serves as an auxin efflux carrier, was identified as a target gene of WRKY-B. We observed a significant downregulation of *PIN4* expression in the *wrky-b* mutant and an upregulation in *WRKY-B-GFP* plants (Fig. 3c). The promoter region of *PIN4* includes four binding motifs for WRKY-B. To test whether WRKY-B can directly bind to the *PIN4* promoter, EMSA was performed with a WRKY-B-GST fusion protein, *PIN4* promoter DNA probes and corresponding mutant probes (Fig. 5a). It was observed that WRKY-B-GST, as opposed to GST alone, exhibited direct binding to the labeled *PIN4* probe, a binding that was found to be inhibited in the presence of competitor probes (Fig. 5b). Additionally, the yeast one-hybrid assay also confirmed the interaction between WRKY-B and the *PIN4* promoter (Fig. 5c). Similarly, the relative intensity of the LUC signals originating from the *PIN4* promoter increased significantly when the reporter was co-transfected with WRKY-B (Fig. 5d and e), confirming that WRKY-B directly activates *PIN4* transcription. We also successfully knocked out *PIN4* and obtained two homozygous mutant lines (Fig. S5, see online supplementary material). Phenotypic and statistical analysis revealed that, similar to the *wrky-b* and *bl* mutants, the *pin4* mutants also exhibited a reduced number and length of lateral branches (Fig. 6). Owing to *PIN4* encoding an auxin efflux carrier, we analysed the levels of IAA in the axillary buds of both WT and *pin4* mutant plants. The endogenous IAA content in the axillary buds of *pin4* mutants was significantly greater than that in the WT (Fig. 7a), which implied that the efflux of auxin from axillary buds to the main stem was blocked in *pin4* mutants. Moreover, the IAA concentrations in the axillary buds of the *wrky-b* and *bl* mutant plants were higher than those in WT (Fig. 7a), suggesting that axillary bud development in these two mutant plants was also inhibited by increasing auxin concentrations.

The tomato Aux/IAA family gene *IAA15*, which encodes a protein that represses auxin-dependent transcription, was found to be involved in axillary shoot development, and tomato IAA15-suppressed lines produced more and longer lateral branches than did the WT [40]. Interestingly, according to our RNA-Seq data, *IAA15* was significantly differentially expressed, and some WRKY-B-binding peaks were found in the *IAA15* promoter (Data Set SS1, see online supplementary material). qRT–PCR revealed that *IAA15* was significantly upregulated in *wrky-b* mutants but downregulated in the *WRKY-B-GFP* plants (Fig. 3c). We hypothesized that WRKY-B can bind directly to the promoter of *IAA15* and inhibit its expression. As expected, EMSA and yeast one-hybrid assays showed that WRKY-B could directly bind to the promoter of *IAA15* both *in vitro* and *in vivo* (Fig. 5a–c). In addition, the transient transcription dual-luciferase reporter assay indicated that *IAA15* transcription was significantly inhibited by WRKY-B (Fig. 5d–e). These results suggest that *IAA15* is also a downstream target gene of WRKY-B during lateral branch development.

## Discussion

### WRKY-B is a key factor in the development of lateral branches in tomato

Plant lateral branching is a crucial trait that influences the overall morphology and reproductive capacity of a plant, consequently impacting plant productivity. WRKY transcription factors, a prominent group of transcriptional regulators found in plants, play crucial roles in various aspects of plant biology, such as growth, development, and reactions to both biotic and abiotic stimuli [41, 42]. Previous studies have demonstrated that several WRKY transcription factors participate in branching development in *Arabidopsis*. For example, *WRKY71*/*EXB1* has been shown to upregulate *RAX* genes, influencing AM initiation and thereby managing lateral branch development [43]. Phylogenetic analysis reveals that *WRKY8*, *WRKY28*, *WRKY48*, and *WRKY57* are closely affiliated with *WRKY71*/*EXB1*. Overexpression of each of these genes—*WRKY8*, *WRKY28*, *WRKY48*, and *WRKY57*—led to increased branching, akin to the branching phenotype seen in transgenic plants overexpressing *WRKY71*/*EXB1* [43]. In addition, WRKY23 plays a redundant role with WRKY71/EXB1 in regulating lateral branching, and *WRKY23*-overexpressing transgenic plants produce more branches than WT plants [44]. Overexpressing *PhWRKY71* in transgenic plants led to a marked rise in the total lateral branches in petunia plants. This effect closely resembled the phenotype observed in *Arabidopsis* plants overexpressing *WRKY71*, suggesting conservation of function in the homologous gene [45].

In our study, we found a WRKY gene (*Solyc02g071130*), named *WRKY-B*, whose expression pattern was similar to the change in IAA content during axillary bud development. As the axillary bud length increased, the expression level of *WRKY-B* gradually decreased (Fig. 1b). We also found that exogenous auxin significantly induced *WRKY-B* transcription (Fig. 1c), indicating that WRKY-B may be involved in the auxin-dependent axillary bud development process. Furthermore, we found that the *wrky-b* mutant plants exhibited a decrease in the number of lateral branches as well as a reduction in their length (Fig. 2). In contrast, *WRKY-B-GFP* plants produced many more lateral branches that were significantly greater in length than those of the WT plants (Fig. 2). Phylogenetic analysis revealed that *WRKY-B* was closely related to *AtWRKY8*, *AtWRKY28*, and *AtWRKY71* (Fig. S1, see online supplementary material). Notably, these homologous genes of *WRKY-B* in *Arabidopsis* had a high degree of functional redundancy in lateral branch development. The single and multiple mutants of *WRKY8*, *WRKY71*, *WRKY48*, and *WRKY57* had no obvious lateral branch development phenotype [43]. Interestingly, single mutants of *WRKY-B* in tomato show no other noticeable developmental defects except for a determinate growth habit and fewer lateral branches. However, we cannot rule out the possibility that high-order mutants of *WRKY-B* homologs in tomato may have a more severe phenotype in terms of lateral branching. Nonetheless, these findings do not prevent us from concluding that WRKY-B plays a key role in the development of lateral branches in tomato plants.

### WRKY-B promoted lateral branching by regulating the auxin pathway

Previous studies have shown that *BL* is a key regulator of lateral branching in tomato plants and can affect AM initiation [20]. Herein, we found that WRKY-B can directly bind to the *BL* gene both *in vitro* and *in vivo* (Fig. 5), positively activating *BL* transcription. The *BL* knockout mutant generated by CRISPR/Cas9 system exhibited a reduced axillary bud phenotype (Fig. 6). Notably, consistent with previous reports [37], the *bl* mutants also exhibited a determinate growth habit phenotype, which was also observed in the *wrky-b* mutants. Therefore, these results indicated that *WRKY-B* acts upstream of *BL* to regulate lateral branching. It was worth noting that previous studies highlighted a critical role of BL in the AM initiation [20, 37]. Interestingly, we found that the percentage of axillary bud outgrowth was significantly lower in *bl* mutants than that in WT plants (Fig. 6), suggesting *BL* also controlled the outgrowth of axillary bud, probably by affecting auxin homeostasis in the buds (Fig. 7).

Many studies have shown that auxin inhibits bud outgrowth and that only minimal quantities of auxin are necessary for AM initiation [43, 46–48]. In this study, we found that WRKY-B was related to auxin-dependent lateral branching (Fig. 1). Combined RNA-Seq and ChIP-Seq analysis identified *PIN4* as a WRKY-B target (Fig. 4). Furthermore, we used EMSA, yeast one-hybrid and transient transcription dual-luciferase reporter experiments to prove that WRKY-B directly binds to the promoter of *PIN4* and activates its expression (Fig. 5). Extensive research has shown that PIN proteins play a crucial role in polar auxin transport, which affects various aspects of plant development, such as lateral branching [49]. The PIN3 protein is known to be a key player in the cytokinin signaling pathway, facilitating the movement of auxin between shoot apices and governing lateral branching [50]. Additionally, *AtPIN3*, *AtPIN4*, and *AtPIN7* have been identified as independent regulators of lateral branching in *Arabidopsis*, operating separately from each other and having a positive impact on this process [51]. Studies have proven that *CsBRC1* suppresses branching in cucumber by inhibiting the transcription of *CsPIN3*, leading to a decrease in auxin transport from lateral buds to the main stem [35].Our results indicated that *PIN4* knockout led to fewer lateral branches in tomato (Fig. 6). The endogenous IAA contents in the axillary buds of the *pin4*, *wrky-b*, and *bl* mutant plants were markedly elevated compared to those in WT (Fig. 7a), suggesting that WRKY-B serves as a critical component that exports auxin from lateral buds to promote bud outgrowth. Interestingly, *WRKY-B* expression was also induced by auxin (Fig. 1c), suggesting that a negative feedback loop works to maintain an optimum auxin level in the lateral buds for shoot branching.

Aux/IAA acts as a repressor of auxin transcription factors in the auxin-mediated gene regulation pathway [52]. The tomato *IAA15* was reported to participate in axillary shoot development. *IAA15*-suppressed lines exhibited an increase in both the number and length of lateral branches compared to those of the WT [40], in contrast to the findings for the *wrky-b* mutant. We revealed that WRKY-B could also target *IAA15* and negatively regulate *IAA15* expression (Fig. 5). Thus, WRKY-B may promote shoot branching by at least three different mechanisms: first, by binding and activating the critical branching gene *BL*; second, by exporting auxin from buds through activating *PIN4*; and third, by preventing the inhibitory effect of auxin on bud growth through *IAA15* repression (Fig. 7b).

## Materials and methods

### Plant materials and growth conditions

The wild-type tomato cultivars *Ailsa Craig* (AC) were utilized for genetic transformation. All the tomato seeds were initially germinated at 28°C in complete darkness. Subsequently, the tomato plants were grown in growth chambers with a temperature ranging between 25°C ± 2°C and subjected to a photoperiod of 16 h light followed by 8 h of darkness. To conduct transient expression and luciferase assays, tobacco plants (*Nicotiana benthamiana*) were cultivated in a greenhouse at 22°C, following a light–dark cycle of 16 h and 8 h, respectively. Pest and water control measures were implemented based on standard procedures to ensure the optimal growth and health of the plants.

### Construction and transformation of binary vectors

The CRISPR/Cas9 system was employed in creating mutants for *WRKY-B*, *PIN4*, and *BL* genes. The unique sgRNA target sequences for *WRKY-B*, *PIN4*, and *BL* were selected based on their coding exons using information from the CRISPR-PLANT database (www.genome.arizona.edu/crispr/). These specific sites were then inserted into the pKSE401 vectors to generate the pKSE401-WRKY-B, pKSE401-BL, and pKSE401-PIN4 constructs following the established protocol [53]. The open reading frame of WRKY-B, which notably lacks a stop codon, was successfully amplified from tomato cDNA. This amplified segment was then specifically cloned into the pCAMBIA1300-GFP vector [54] to create the *Pro35S*::*WRKY-B*-*GFP* vector. A 3000-bp sequence upstream of the *WRKY-B* gene ATG codon was cloned into the pCAMBIA1301 vector to create the *ProWRKY-B*::*GUS* vector. The binary vectors required for genetic transformation were both introduced into AC using the *Agrobacterium tumefaciens* strain GV3101, following the previously described method [55]. The CTAB method, as described by Murray *et al.* [56], was utilized to extract the genomic DNA from the transgenic plants for PCR amplification and subsequent DNA sequencing. The primers used for construction are listed in Table S1 (see online supplementary material).

### Subcellular localization and β-glucuronidase (GUS) staining

The subcellular localization of WRKY-B was examined by transiently transforming the WRKY-B-GFP fusion vector into tobacco leaves [57], followed by imaging with a confocal laser-scanning microscope (Leica SP8,Wetzlar, Germany). GUS staining was performed to assess the expression of *WRKY-B* in the axillary buds. Briefly, transgenic tomato plants harboring *ProWRKY-B*::*GUS* were stained with a GUS staining kit (Coolaber, Beijing, China) and images were captured with a stereomicroscope (Nikon AZ100 microscope, Tokyo, Japan).

### Hormone treatment and gene expression analyses

For hormone treatment, 2–3 leaf-old AC tomato seedlings were treated with 50 μM IAA, and the leaves were collected 0, 1, and 3 h after IAA treatment. Three biological replicates were performed for the treatment, and each biological replicate consisted of 10 pooled seedlings. For gene expression analyses, RNA was isolated with the TRIzol reagent (Invitrogen, Carlsbad, CA, USA) and reverse transcription was executed with the SuperScript III kit (Invitrogen, Carlsbad, CA, USA). qRT–PCR analysis was carried out with the SYBR Green Master Mix (Toyobo, Shanghai, China) as per a previous protocol [58]. All gene expression levels were normalized to the tomato ACTIN2 gene. All primers used are listed in Table S1 (see online supplementary material).

### Analysis of RNA-Seq data

RNA-seq analysis was carried out on axillary buds from the sixth nodes of tomato plants at the 45-d-old seedling stage, including both WT and *WRKY-B-GFP* plants. All tomato plants were collected from three biological replicates with 10 plants per replicate. Libraries were constructed using total RNA and sequenced on an Illumina HiSeq 2000 platform. The genome version SL4.0 of tomato was acquired from the SOL Genomics Network (http://solgenomics.net/). The RNA-Seq data analysis was conducted according to the methods described previously [59].

### ChIP assays

Sampling the lateral buds of ChIP assays was the same as the RNA-Seq sampling described above, and ChIP-Seq was conducted according to a previous protocol [60]. In brief, the samples underwent cross-linking with 1% formaldehyde (v/v) under vacuum for 10 minutes and were then pulverized into powder using liquid nitrogen. Initially, the chromatin complexes were separated, followed by sonication and immunoprecipitation with polyclonal anti-GFP antibodies from Sigma-Aldrich. The ChIP DNA obtained was then resuspended in water to prepare Illumina sequencing libraries. The Illumina HiSeq 2500 platform was employed for ChIP-seq analysis. Fastqc software version 0.11.5 was applied to screen for clean reads. These clean reads were subsequently mapped to the reference genome utilizing BWA software (version 0.7.15-r1140) [61].

### Electrophoretic mobility shift assays

The *WRKY-B* full-length CDS was attached to the pGEX4T vector, resulting in the WRKY-B-GST plasmid, which was then inserted into the BL21 (DE3) strain of *Escherichia coli*. The GST-fusion proteins were recombinantly expressed in *E. coli* BL21 and subsequently purified by affinity using glutathione beads (GE Healthcare, Chicago, IL, USA). Probes harboring specific *cis*-elements of *BL*, *PIN4*, and *IAA15* were synthesized separately and biotin-labeled. Unlabeled wild-type and mutant probes with specific transcription factor binding motifs mutational were used as competitors. The EMSA Probe Biotin Labeling Kit and Chemiluminescent EMSA Kit (Beyotime, Shanghai, China) were used for the EMSA according to the manufacturer’s instructions. Probes used for EMSA are listed in Table S1 (see online supplementary material).

### Yeast one-hybrid and transcriptional activity assays

In the yeast one-hybrid assay, the CDS of *WRKY-B* was cloned into the pGADT7 vector to create the plasmid pGADT7-WRKY-B.A 400-bp fragment (−4600 to −4999 bp upstream of ATG) of the *BL* promoter, a 320-bp fragment (−3200 to −3519 bp upstream of ATG) of the *PIN4* promoter, and a

180-bp fragment (−4260 to −4439 bp upstream of ATG) of the *IAA15* promoter were inserted into the pHIS2 vector to obtain BL-pHIS2 PIN4-pHIS2 and IAA15-pHIS2, respectively. Following this, pGADT7-WRKY-B and BL-pHIS2, pGADT7-WRKY-B and PIN4-pHIS2, and pGADT7-WRKY-B and IAA15-pHIS2 were co-transformed into the competent Y187 yeast strain (AngYuBio, Shanghai, China). Successful transformation was confirmed using SD/-WL (Synthetic Dropout Medium/−Tryptophan-Leucine) medium and screening on SD/-WHL (Synthetic Dropout Medium/−Tryptophan-Histidine-Leucine) medium supplemented with 445 mM 3-amino-1, 2,4-triazole (3-AT). For the analysis of transcriptional activity, recombinant plasmids pGBKT7-WRKY-B and pGADT7 were co-transformed into AH109 yeast cells (AngYuBio, Shanghai, China). The yeast cells were grown on SD/-WL medium at 30°C for 48–60 h, then streaked on to SD/-WLHA (Synthetic Dropout Medium/−Tryptophan-Leucine-Histidine-Adenine) medium for growth observation.

### Transient transcription dual-luciferase reporter assay

To generate reporter vectors, the 5000 bp promoters of *BL*, *PIN4*, and *IAA15* were individually cloned into the pGreenII-0800-LUC vector [62]. Additionally, the entire *WRKY-B* coding sequence was inserted into the pGreenII 62-SK vector to create an effector vector. These recombinant vectors were then transformed into the *A. tumefaciens* strain GV3101. Various combinations of infiltration agents were prepared and introduced into tobacco leaves. Following infiltration, the tobacco plants were placed in darkness at 21°C for 12 h, then exposed to normal light for 48 h. The leaves were sprayed with a solution of D-luciferin potassium salt containing 0.1% Triton X-100, and images were captured using the Night SHADE LB 985 system (Berthold Technologies, Bad Wildbad, Germany). The LUC/REN ratio was calculated by employing the Dual-Luciferase® Reporter Assay kit (Promega, Madison, WI, USA) in accordance with the provided guidelines.

### Quantification of endogenous auxins

To analyse IAA levels, axillary buds were harvested from the sixth nodes of WT, *wrky-b*, *bl*, and *pin4* plants at the 45-d-old seedling stage. Three biological replicates, each consisting of 10 plants, were used in this study. Afterward, the levels of endogenous auxins were measured via a high-performance liquid chromatography (HPLC) system with electrospray ionization/MS–MS capability (Alliance HPLC 1525, Milford, MA, USA). The equipment employed for assessing IAA concentrations included a UV detector (SPD-20A), a column temperature regulator (CTO-20 AC), and a C18 reversed-phase chromatography column (dimensions: 150 mm $\times$ 4.6 mm, particle size: 5 μm). The rate of flow was modified to 0.8 mL/min, the temperature of the column was established at 30°C, and ultraviolet detection was performed at a wavelength of 254 nm [63].

## Supplementary Material

Web_Material_uhae193

## Data Availability

The raw RNA-seq data have been deposited in Gene Expression Omnibus (GEO) of NCBI (https://www.ncbi.nlm.nih.gov/) under the accession number GSE164382. Other data analysed during the study was included in this article.
