# Supplementary material for: The tomato WRKY-B transcription factor modulates lateral branching by targeting BLIND, PIN4, and IAA15
Source: Hortic Res. 2024 Jul 11;11(9):uhae193. doi: 10.1093/hr/uhae193 (PMC11384121; doi:10.1093/hr/uhae193)
Supplement: Web_Material_uhae193 [file web_material_uhae193.zip › Supplemental Data Set 1.pdf]

Dataset S1. List of genes that were identified by RNA-seq and CHIP-seq.

Overlap(510)	logFC	P.Value	adj.P.Val	Regulated.Type
Solyc00g013160.2	-1.739539538	0.011383634	0.04438103	Down
Solyc00g021640.3	3.073248982	0.036669062	0.095003247	Up
Solyc00g179240.2	2.280323295	0.012821929	0.047582785	Up
Solyc01g005120.3	-2.119911624	0.000767402	0.009380506	Down
Solyc01g006320.3	-1.43623204	0.020033155	0.063666002	Down
Solyc01g006450.3	-1.973203471	0.0010001	0.010688908	Down
Solyc01g006825.1	-1.85349475	0.004979339	0.026737713	Down
Solyc01g008790.3	-1.721844307	0.017809748	0.058884199	Down
Solyc01g011025.1	-1.463349038	0.000945915	0.010424816	Down
Solyc01g013885.1	1.472251438	0.021814564	0.067277394	Up
Solyc01g028860.3	1.535446473	0.004820434	0.026196858	Up
Solyc01g044240.3	-1.138290567	0.00499545	0.026790856	Down
Solyc01g050040.3	2.500141068	0.002662626	0.01842469	Up
Solyc01g057780.3	1.256587826	0.008503036	0.03713313	Up
Solyc01g065495.1	1.793592003	0.007841439	0.035274202	Up
Solyc01g067130.3	3.623296746	0.0357281	0.093255404	Up
Solyc01g067660.3	1.154264828	0.032288363	0.087128497	Up
Solyc01g079400.3	1.650334256	0.016066509	0.054999515	Up
Solyc01g079570.3	-3.919334695	3.27013E-05	0.002188097	Down
Solyc01g079580.3	2.434610242	0.004801802	0.026159103	Up
Solyc01g079620.3	3.981345287	0.015710261	0.054240114	Up
Solyc01g079740.3	-1.851218408	0.006041432	0.029782289	Down
Solyc01g086870.3	1.144920253	0.013780056	0.04980412	Up
Solyc01g087570.2	-1.733022094	0.00918526	0.038942132	Down
Solyc01g087590.3	1.328325866	0.012834962	0.047597007	Up
Solyc01g087970.3	-4.985303489	7.93955E-06	0.001252222	Down
Solyc01g088100.3	1.263579069	0.004461573	0.025076716	Up
Solyc01g088400.3	-1.87714178	0.041630521	0.103143205	Down
Solyc01g088660.3	-1.45675741	0.004721023	0.025915533	Down
Solyc01g089880.3	-1.864772619	0.004734503	0.025956477	Down
Solyc01g090460.3	4.565374131	0.006119743	0.030042376	Up
Solyc01g090790.3	1.724142619	0.01470994	0.051953596	Up
Solyc01g091000.3	19.52292884	0.017744082	0.058766764	Up
Solyc01g091620.3	1.535501422	0.003768605	0.022680021	Up
Solyc01g094010.3	-1.336355415	0.014818467	0.052184407	Down
Solyc01g094750.3	-1.947642975	0.014542138	0.051569732	Down
Solyc01g095070.3	-2.171154515	0.000921387	0.010286765	Down
Solyc01g095180.2	-1.127047396	0.001826955	0.014874418	Down
Solyc01g095620.3	1.052864726	0.005514664	0.028229228	Up
Solyc01g096660.3	-1.191566879	0.000277112	0.005523065	Down
Solyc01g098390.3	3.161199547	0.025196674	0.07380106	Up
Solyc01g099340.3	2.16659808	0.00229529	0.016919262	Up
Solyc01g099630.3	-3.970487348	1.01117E-05	0.001391222	Down
Solyc01g099880.3	-3.251252876	0.00036156	0.006280928	Down
Solyc01g100040.3	2.295169886	0.002426064	0.017440963	Up
Solyc01g100050.3	1.000727715	0.018923893	0.061431402	Up
Solyc01g100310.2	2.28326673	0.032192149	0.086955109	Up
Solyc01g100980.3	-1.726981506	0.008470435	0.03704592	Down

Solyc01g102350.3	-1.238123989	0.005694277	0.028778463	Down
Solyc01g102390.3	-1.14062164	0.002161283	0.016341346	Down
Solyc01g102580.3	-1.048276624	0.016686322	0.056412025	Down
Solyc01g102730.3	-1.255494597	0.010903793	0.043263924	Down
Solyc01g103590.3	1.796709362	0.029234591	0.081283664	Up
Solyc01g104015.1	-2.074375792	0.000953801	0.010461293	Down
Solyc01g105010.3	-2.072325436	0.000451066	0.007003172	Down
Solyc01g105190.3	1.697971463	0.032382242	0.087295368	Up
Solyc01g105410.3	4.454790876	0.04101775	0.102202593	Up
Solyc01g106910.3	3.726317138	0.006718102	0.031773577	Up
Solyc01g107820.2	-1.028285997	0.008004116	0.035788152	Down
Solyc01g108087.1	2.281845778	0.014185094	0.050722904	Up
Solyc01g108230.3	-1.172309228	0.000681236	0.008849413	Down
Solyc01g108300.3	1.815184501	0.023803701	0.071179945	Up
Solyc01g108440.2	1.555061015	0.032362934	0.087252413	Up
Solyc01g108540.3	1.64063811	0.043069176	0.105385233	Up
Solyc01g108610.3	-3.890147332	0.000167272	0.004433042	Down
Solyc01g109530.3	-25.43777696	0.006540432	0.031264983	Down
Solyc01g109700.3	-1.558364917	0.001777512	0.014675108	Down
Solyc01g110130.3	-2.004233441	0.000304749	0.005783308	Down
Solyc01g110380.3	-1.684660084	0.009178194	0.038931317	Down
Solyc01g111145.1	-4.953027853	0.001664089	0.014137332	Down
Solyc01g111360.3	-1.056401963	0.005018812	0.026860458	Down
Solyc01g111500.3	-3.689406104	1.60413E-05	0.001693568	Down
Solyc01g112150.3	-1.041242028	0.000146901	0.0041482	Down
Solyc02g030080.3	1.87126394	0.00480102	0.026159103	Up
Solyc02g032650.3	1.748797957	0.034364596	0.090877685	Up
Solyc02g036370.3	2.337027179	0.041187565	0.102467785	Up
Solyc02g037550.3	-5.680981713	0.002457497	0.017564415	Down
Solyc02g062690.3	-1.073037544	0.008524059	0.037174093	Down
Solyc02g062710.1	-2.634950637	0.008978713	0.038450893	Down
Solyc02g063000.3	-4.176077228	0.00238962	0.017299163	Down
Solyc02g063150.3	-1.349126673	0.000147958	0.004173479	Down
Solyc02g063360.3	-2.049243519	0.010575823	0.04254515	Down
Solyc02g065280.3	-3.435398879	0.000522472	0.007571013	Down
Solyc02g067050.3	2.382556729	0.04914845	0.115371068	Up
Solyc02g067230.3	2.230209327	0.019065023	0.061697901	Up
Solyc02g067310.3	1.294575177	0.021061323	0.065730233	Up
Solyc02g067350.3	-2.48169323	0.00128388	0.012263239	Down
Solyc02g067530.3	-1.199308808	0.002246153	0.016704715	Down
Solyc02g068040.3	20.28395046	0.00868644	0.037603476	Up
Solyc02g068920.3	1.495995055	0.00349331	0.021708373	Up
Solyc02g069190.3	6.335653069	0.035108168	0.092146917	Up
Solyc02g069470.3	-1.142107057	0.002738987	0.018732586	Down
Solyc02g070280.3	-1.747469057	0.00043566	0.006885412	Down
Solyc02g071220.3	-2.077950145	0.001922392	0.015268131	Down
Solyc02g071610.3	-1.356797533	0.014803819	0.052147023	Down
Solyc02g071740.3	1.628141269	0.001158996	0.011583694	Up
Solyc02g071990.3	1.390534483	0.022519854	0.068636822	Up
Solyc02g072150.3	1.65029137	0.038046566	0.097071659	Up

Solyc02g077040.4	-1.863660544	0.001327418	0.012521883	Down
Solyc02g077080.3	-1.292720656	0.003108541	0.020237988	Down
Solyc02g077420.3	-4.88842093	0.002076431	0.015984903	Down
Solyc02g077620.1	1.637182184	0.00625041	0.030458387	Up
Solyc02g077710.1	-3.057496223	0.000436965	0.006887127	Down
Solyc02g077720.3	-1.996161341	3.30228E-06	0.000910024	Down
Solyc02g078040.3	2.892102169	0.020154661	0.063942164	Up
Solyc02g078170.2	2.142677301	0.00851372	0.037142163	Up
Solyc02g079440.2	-3.915581582	0.006101956	0.029983508	Down
Solyc02g079570.3	1.974341045	0.000101064	0.003549248	Up
Solyc02g080200.3	-2.920643751	0.00040969	0.006677397	Down
Solyc02g080450.1	-5.706469659	0.000409451	0.006677397	Down
Solyc02g081330.4	-1.566565944	0.012676069	0.047354015	Down
Solyc02g081390.3	-2.666782293	2.38445E-05	0.001935874	Down
Solyc02g082150.1	-1.865695702	0.049653229	0.116101174	Down
Solyc02g083450.3	-1.327636722	0.003469585	0.021625138	Down
Solyc02g084420.3	1.32125723	0.01910195	0.061800004	Up
Solyc02g084600.3	-1.433201166	0.003195452	0.020555477	Down
Solyc02g084720.3	-1.222320053	0.024276057	0.072051004	Down
Solyc02g084950.3	1.14219271	0.001530647	0.013535627	Up
Solyc02g084990.3	-1.734980969	3.06545E-05	0.002143001	Down
Solyc02g085000.3	-3.128547954	0.000777896	0.009433717	Down
Solyc02g085160.1	2.194889345	0.005662757	0.028658358	Up
Solyc02g085190.2	1.988442969	0.012003775	0.045849033	Up
Solyc02g085360.3	-2.828339947	0.000658752	0.008670207	Down
Solyc02g085750.3	-1.849378137	0.035735406	0.093263244	Down
Solyc02g086590.3	1.384168674	0.033831732	0.090043118	Up
Solyc02g086670.3	1.317662786	0.002996723	0.019804095	Up
Solyc02g086700.3	-3.087458598	0.00439823	0.024861146	Down
Solyc02g086840.3	-2.008461579	0.000919132	0.010286765	Down
Solyc02g087770.3	-1.722946362	0.001667872	0.014139929	Down
Solyc02g089640.3	-1.480805269	0.000102125	0.003562626	Down
Solyc02g089840.3	-3.39501426	0.006739923	0.031847799	Down
Solyc02g089990.1	-1.622841436	0.004652371	0.025707803	Down
Solyc02g090350.3	-1.044430744	0.005195676	0.027357664	Down
Solyc02g090870.1	-1.048541143	0.00067151	0.008776799	Down
Solyc02g091430.3	1.380417429	0.01685621	0.056860031	Up
Solyc02g092580.3	-2.365791909	0.000438249	0.006895224	Down
Solyc02g092750.3	1.761914602	0.021928968	0.067498225	Up
Solyc02g092790.3	-3.219863402	2.64154E-05	0.002004386	Down
Solyc02g092820.3	2.408399712	0.002095758	0.016051873	Up
Solyc02g092840.1	-1.896542688	0.001556025	0.013643441	Down
Solyc02g093050.3	1.582151599	0.038520096	0.097874342	Up
Solyc02g093150.3	-1.009033004	0.004821899	0.026196858	Down
Solyc02g093180.3	-4.616409686	9.52863E-06	0.001376914	Down
Solyc02g093270.3	-1.160783954	0.041601497	0.1030968	Down
Solyc02g093680.3	-1.622474917	0.006084583	0.029937373	Down
Solyc02g093700.3	-3.89024793	0.000186048	0.004609817	Down
Solyc02g093860.3	-2.9065818	0.001501662	0.013404324	Down
Solyc02g094030.3	-2.492539133	3.69949E-05	0.002311376	Down

Solyc02g094300.3	1.276175082	7.15152E-06	0.001201131	Up
Solyc03g005770.3	-2.447561462	7.24142E-05	0.002979978	Down
Solyc03g007780.1	-27.12047056	0.002805501	0.018962933	Down
Solyc03g013600.1	1.431883171	0.041443744	0.102897282	Up
Solyc03g025730.3	-1.032762074	0.005070181	0.0269992	Down
Solyc03g026360.1	-3.03184629	0.019019114	0.061601354	Down
Solyc03g032000.3	-1.069839625	0.002230647	0.016646832	Down
Solyc03g033590.1	-3.292362574	0.00169899	0.014267058	Down
Solyc03g033840.3	-1.501346382	0.016559822	0.056153168	Down
Solyc03g043640.3	-1.560983638	0.012479216	0.046996645	Down
Solyc03g053027.1	1.189230206	0.034050456	0.090320899	Up
Solyc03g058160.3	-2.122030311	0.005168426	0.027281332	Down
Solyc03g078640.1	-1.351700135	0.024510971	0.07251528	Down
Solyc03g079980.2	-3.987927168	0.01021794	0.041628167	Down
Solyc03g080110.3	-2.288977673	0.000165242	0.004398297	Down
Solyc03g083360.3	-3.068692039	0.017526354	0.058239553	Down
Solyc03g083440.3	-2.17160499	0.008206395	0.036361293	Down
Solyc03g083460.3	-1.256579384	0.013810587	0.049845922	Down
Solyc03g093140.3	-1.012478126	0.004885689	0.026410286	Down
Solyc03g096050.3	-1.090504689	0.031960562	0.086555532	Down
Solyc03g096390.3	-1.967929653	1.65876E-05	0.001699699	Down
Solyc03g097120.3	1.97374807	0.022242011	0.06809725	Up
Solyc03g097690.3	-1.206653774	0.023880903	0.07133658	Down
Solyc03g098630.3	1.686819486	0.034423662	0.09094091	Up
Solyc03g111690.3	-4.304696359	0.00040615	0.006655197	Down
Solyc03g112060.3	-1.975020856	2.66082E-05	0.002004797	Down
Solyc03g112297.1	2.875025422	0.031633518	0.08603013	Up
Solyc03g112340.1	-1.342906644	0.000960508	0.010509515	Down
Solyc03g112460.3	-1.923498598	0.001641412	0.014046852	Down
Solyc03g113420.3	-1.850882298	0.004823814	0.026201756	Down
Solyc03g114020.3	1.925452739	0.009736493	0.040392003	Up
Solyc03g114030.3	-2.201129951	0.002791145	0.01891922	Down
Solyc03g114160.1	-5.636969804	3.05225E-06	0.000897152	Down
Solyc03g114690.3	-2.592230501	0.002040173	0.015818681	Down
Solyc03g114730.3	-1.231873876	0.003466323	0.021620427	Down
Solyc03g115247.1	-20.06907209	0.025135287	0.07373403	Down
Solyc03g115990.2	-1.609058572	0.000312337	0.005850996	Down
Solyc03g116100.3	1.439950069	0.029553729	0.081906862	Up
Solyc03g116320.3	1.508109582	0.00120426	0.011848383	Up
Solyc03g116350.3	1.75842571	0.034031131	0.090300495	Up
Solyc03g116480.1	-2.134152314	0.015592494	0.053958708	Down
Solyc03g116730.3	-2.062209921	0.002237526	0.016672692	Down
Solyc03g116770.3	-17.19460298	0.017317552	0.057894519	Down
Solyc03g117210.3	2.513436745	0.019582692	0.06279766	Up
Solyc03g117350.1	-1.890726518	0.000252722	0.005318877	Down
Solyc03g117420.3	2.916728524	0.022119239	0.067909346	Up
Solyc03g117590.3	2.79046433	0.008056044	0.035955372	Up
Solyc03g117600.3	-1.306761028	0.029421625	0.081631891	Down
Solyc03g117675.1	3.296094022	0.018549217	0.060625984	Up
Solyc03g117980.3	1.547359923	0.004227633	0.02424501	Up

Solyc03g118040.3	-1.100716745	0.002078222	0.015989078	Down
Solyc03g119260.3	-5.394216474	0.002608788	0.01822769	Down
Solyc03g119540.3	-1.600281605	0.015992365	0.054834933	Down
Solyc03g120170.1	-1.987093437	0.038259424	0.097403373	Down
Solyc03g120390.3	-1.91177388	0.000759027	0.009322412	Down
Solyc03g120550.2	-1.484885685	0.00053128	0.007638742	Down
Solyc03g120810.3	-1.259478579	0.025231373	0.07384591	Down
Solyc03g120890.3	-2.536257892	0.001848058	0.014970831	Down
Solyc03g121190.3	-2.422062512	0.018967081	0.061468009	Down
Solyc03g121400.1	-1.787221779	0.002968116	0.019670326	Down
Solyc03g123420.1	-1.970899467	0.000906563	0.01023534	Down
Solyc03g123490.1	1.265159718	0.025947136	0.075165037	Up
Solyc04g005650.2	-1.852755872	0.026318289	0.075816226	Down
Solyc04g005660.3	-4.754858649	0.035360933	0.092612989	Down
Solyc04g005800.3	3.785798779	0.021848965	0.067345427	Up
Solyc04g007200.3	-4.490834963	3.69367E-05	0.002311376	Down
Solyc04g007270.3	1.413639447	0.021077502	0.065764858	Up
Solyc04g007660.1	-1.855902667	0.003746391	0.022598916	Down
Solyc04g007870.3	-1.200506609	0.013090515	0.048178609	Down
Solyc04g008480.2	5.792954974	0.037811808	0.096707139	Up
Solyc04g009900.3	-1.290608422	0.017458504	0.058078973	Down
Solyc04g012120.3	-1.59054051	0.001352511	0.012666206	Down
Solyc04g015020.3	-2.221208735	1.63567E-05	0.001699699	Down
Solyc04g015600.3	1.761299106	0.025181392	0.073798085	Up
Solyc04g015850.2	-1.216477093	0.017610593	0.058436895	Down
Solyc04g016250.3	1.458430095	0.004787856	0.026143695	Up
Solyc04g016460.3	-1.744788127	0.009393841	0.039528404	Down
Solyc04g039850.1	-1.26456967	0.026464142	0.076007273	Down
Solyc04g049090.3	-1.626271115	0.005623419	0.028554252	Down
Solyc04g050440.3	-2.302863833	0.001120591	0.011388162	Down
Solyc04g050490.3	-1.577873076	0.001341845	0.012607395	Down
Solyc04g051513.1	-15.60964047	0.037750977	0.096624303	Down
Solyc04g055090.1	-2.053980168	0.00168933	0.014238254	Down
Solyc04g071030.1	-1.014216606	0.002525642	0.017838588	Down
Solyc04g071360.3	-2.953772821	8.62415E-05	0.003261291	Down
Solyc04g071480.1	-1.031590138	0.003129986	0.020321339	Down
Solyc04g072020.3	2.642916388	0.001843437	0.014952689	Up
Solyc04g072480.3	-6.539086678	0.003076664	0.020104747	Down
Solyc04g072850.3	-2.697744047	0.002573776	0.018051325	Down
Solyc04g074290.3	-2.161829697	0.002617393	0.018274759	Down
Solyc04g074680.1	-3.136372442	0.002244237	0.016699176	Down
Solyc04g077450.3	1.927497625	0.035100705	0.092136678	Up
Solyc04g077500.3	1.096215315	0.004799563	0.026159103	Up
Solyc04g077510.3	5.216499559	0.01637656	0.055685041	Up
Solyc04g078390.2	1.07985483	0.01136296	0.044317601	Up
Solyc04g078560.1	-1.867201117	0.032129668	0.086867983	Down
Solyc04g079700.3	-2.073351742	0.030787978	0.084322774	Down
Solyc04g080130.3	3.581320697	0.004364329	0.024728208	Up
Solyc04g080450.1	1.279375354	0.000285435	0.00560596	Up
Solyc04g081250.1	-3.565846085	0.001305405	0.012386506	Down

Solyc04g081300.3	-1.50003602	0.004157095	0.024039218	Down
Solyc04g081550.3	-2.834079994	0.003385851	0.021318995	Down
Solyc04g082030.1	-1.423102668	0.037585588	0.096424427	Down
Solyc04g082710.3	-2.688990298	0.003945855	0.023323453	Down
Solyc04g082970.3	-1.503138278	0.003529704	0.02185949	Down
Solyc05g008060.2	1.27829521	0.021692493	0.067060837	Up
Solyc05g005160.3	-2.303594601	0.002745611	0.018758049	Down
Solyc05g005170.3	-1.69819926	0.018206132	0.059799343	Down
Solyc05g005490.3	-5.231874377	3.91391E-05	0.00231265	Down
Solyc05g005560.4	-2.246847005	0.003320189	0.021084893	Down
Solyc05g006400.2	1.094504493	0.009721997	0.040363223	Up
Solyc05g006510.1	-2.657736101	0.000506066	0.007429001	Down
Solyc05g007830.3	-3.947159395	5.28815E-05	0.002666339	Down
Solyc05g009320.3	-18.0950673	0.036580922	0.094838342	Down
Solyc05g009840.3	1.584279487	0.010980486	0.043387532	Up
Solyc05g009900.2	1.761487913	0.044564379	0.107844767	Up
Solyc05g011930.3	2.014664291	0.002984866	0.019730778	Up
Solyc05g013530.3	-1.549541106	1.16733E-05	0.001482755	Down
Solyc05g013580.3	-1.505258497	0.002377217	0.017228661	Down
Solyc05g013680.3	-1.402856593	0.025061174	0.073604216	Down
Solyc05g014000.3	-7.036929673	0.00024155	0.005219652	Down
Solyc05g015290.1	2.055689374	0.009690215	0.040277534	Up
Solyc05g015420.3	1.879351436	0.004618075	0.025574821	Up
Solyc05g015840.3	3.851922869	0.002125785	0.016167466	Up
Solyc05g021090.3	1.415037499	0.033822812	0.09003405	Up
Solyc05g051290.3	1.984624013	0.031408908	0.085608305	Up
Solyc05g051400.3	-2.960595537	0.000428871	0.006838626	Down
Solyc05g051900.3	2.213298057	0.00188017	0.015117339	Up
Solyc05g052030.1	1.301169535	6.3817E-05	0.002860615	Up
Solyc05g052050.1	1.531543318	0.02003308	0.063666002	Up
Solyc05g053340.3	-1.424497829	0.006572174	0.031358763	Down
Solyc05g053550.3	1.751887148	0.038041794	0.097069058	Up
Solyc05g054440.3	-29.22604382	0.001127086	0.011419193	Down
Solyc05g055400.3	-4.493688223	0.000375688	0.006397335	Down
Solyc06g005390.1	-6.479321171	6.11021E-05	0.002822213	Down
Solyc06g005500.3	1.625509651	0.001627478	0.013968618	Up
Solyc06g009380.3	-1.896937815	9.77208E-05	0.00346575	Down
Solyc06g035560.2	-1.10093536	0.004638271	0.025657297	Down
Solyc06g035960.3	-23.93606996	0.001175449	0.011684898	Down
Solyc06g036130.3	-1.068723519	0.004236552	0.024259905	Down
Solyc06g049040.3	-1.490325627	0.00143395	0.013050858	Down
Solyc06g049050.3	-4.327560014	3.89942E-06	0.000920766	Down
Solyc06g050130.3	1.550719713	0.005932747	0.029471181	Up
Solyc06g050370.1	-1.183661453	0.001331551	0.012547137	Down
Solyc06g050590.3	-1.533577702	0.000317717	0.00588007	Down
Solyc06g050700.3	3.14921468	0.01328515	0.048611356	Up
Solyc06g051460.3	2.070593473	0.015834121	0.054528743	Up
Solyc06g051750.3	-1.720427544	0.003917331	0.023239833	Down
Solyc06g053653.1	1.565119964	0.019571508	0.062769576	Up
Solyc06g053720.2	-1.640168003	0.021535435	0.066708681	Down

Solyc06g053810.3	2.056617696	0.022533396	0.068651216	Up
Solyc06g053840.3	1.304930175	0.009649519	0.040147089	Up
Solyc06g060120.3	2.634328814	0.006912273	0.032343138	Up
Solyc06g060610.2	-1.182447964	0.000687024	0.008876406	Down
Solyc06g061240.3	1.408113684	0.025135758	0.07373403	Up
Solyc06g061260.1	-23.52412527	0.013248286	0.048565533	Down
Solyc06g063120.3	-3.46994584	0.000274651	0.005507884	Down
Solyc06g063295.1	2.437513578	0.003639329	0.02218174	Up
Solyc06g066340.3	1.268079126	0.022168876	0.067980272	Up
Solyc06g066570.3	18.12901463	0.039091407	0.098859829	Up
Solyc06g068140.3	2.133023242	0.0021839	0.016449846	Up
Solyc06g068840.3	1.361207669	0.005972629	0.029602833	Up
Solyc06g069697.1	1.518079891	0.002452281	0.017536826	Up
Solyc06g069760.3	2.415552418	0.026533342	0.076170732	Up
Solyc06g069770.3	1.852726197	0.003367891	0.021288825	Up
Solyc06g071420.3	1.240955152	0.011697743	0.045136194	Up
Solyc06g071510.3	-1.100996227	0.000345277	0.006163787	Down
Solyc06g071950.2	-1.839279689	0.023856173	0.071270938	Down
Solyc06g073245.1	-7.562584493	0.000643547	0.008553947	Down
Solyc06g073860.2	1.290770978	0.004321322	0.024593029	Up
Solyc06g074040.1	-3.08648255	0.000141899	0.004082708	Down
Solyc06g074810.3	1.042196564	0.008400276	0.03689617	Up
Solyc06g075130.3	-5.819427754	0.002777559	0.018857473	Down
Solyc06g075690.3	2.078845947	0.046274871	0.110500906	Up
Solyc06g076090.3	-10.98366023	0.000351853	0.00622253	Down
Solyc06g076490.3	1.39202109	0.017424672	0.05801591	Up
Solyc06g076750.3	1.455994174	0.00270761	0.018591561	Up
Solyc06g082530.2	-2.640457613	0.000196242	0.004721852	Down
Solyc06g082950.3	-1.780763621	0.001288864	0.012283628	Down
Solyc06g083310.3	-1.30425831	7.63737E-05	0.003067521	Down
Solyc06g083650.3	-4.457456943	0.000102456	0.003562626	Down
Solyc06g083680.3	-1.321433554	0.002514539	0.017799963	Down
Solyc07g005960.3	-1.304537958	0.016970564	0.057111155	Down
Solyc07g006220.2	-3.630816712	0.000988062	0.010649831	Down
Solyc07g006310.1	-2.654864514	0.003580207	0.022033221	Down
Solyc07g008280.3	2.27976937	0.037418157	0.096170314	Up
Solyc07g017780.3	-1.639947874	0.001685436	0.014219015	Down
Solyc07g032110.3	2.058671291	1.5637E-08	0.000124185	Up
Solyc07g041620.1	1.40661014	0.000940174	0.01039677	Up
Solyc07g043000.3	-3.91744469	0.001368526	0.012742368	Down
Solyc07g047850.3	-1.223961077	0.001495512	0.013386914	Down
Solyc07g051840.3	-4.555981794	0.011463558	0.044584886	Down
Solyc07g053220.2	1.476212699	0.016324705	0.055596422	Up
Solyc07g053300.1	-6.414941323	0.007421353	0.034096044	Down
Solyc07g053420.3	-2.478971805	0.030735409	0.084203109	Down
Solyc07g054950.2	4.25009535	0.029687454	0.082171823	Up
Solyc07g055210.3	-1.256287573	0.000406526	0.006655197	Down
Solyc07g056000.2	-2.966036997	0.004071329	0.023696891	Down
Solyc07g056150.3	-1.044838482	0.000290879	0.005671736	Down
Solyc07g062250.3	1.818553129	0.013764388	0.049773475	Up

Solyc07g062660.3	1.41314196	0.004508355	0.025262804	Up
Solyc07g062680.2	-1.75023114	0.016685569	0.056412025	Down
Solyc07g062740.3	-2.686084425	3.83269E-05	0.00231265	Down
Solyc07g063350.3	-1.807354922	0.004760174	0.026058553	Down
Solyc07g063460.2	-1.083288658	0.0011055	0.011306787	Down
Solyc07g063850.3	3.13827314	0.017803664	0.058873636	Up
Solyc07g063860.3	-1.261124816	0.002092245	0.016044471	Down
Solyc07g065820.3	-2.049586456	6.01406E-05	0.002805223	Down
Solyc07g066260.3	1.185452733	0.007611932	0.034639378	Up
Solyc07g066550.3	3.623464401	0.000991977	0.010668148	Up
Solyc08g005490.3	-2.863498	0.00506304	0.026987291	Down
Solyc08g006750.3	-1.95300252	0.016143225	0.055203683	Down
Solyc08g008080.1	-2.377956634	5.94578E-05	0.002805223	Down
Solyc08g008610.3	-1.4559651	0.040239823	0.100749518	Down
Solyc08g016215.1	-1.548433018	0.044804376	0.108177207	Down
Solyc08g041890.3	1.421482071	0.00318255	0.02051329	Up
Solyc08g062490.3	-1.22084197	0.036119675	0.093943841	Down
Solyc08g066510.3	-5.965372027	0.002186825	0.016462283	Down
Solyc08g066650.3	-4.333876692	0.025866294	0.07498964	Down
Solyc08g066840.3	-2.302955377	0.00029549	0.005689992	Down
Solyc08g067320.2	-1.370242775	0.015476352	0.053710213	Down
Solyc08g074650.3	-3.946406937	0.0192872	0.062184888	Down
Solyc08g075120.3	-1.261998003	0.014705205	0.051953596	Down
Solyc08g075290.2	-1.827163403	0.027195857	0.077455191	Down
Solyc08g075950.2	4.813600792	0.037927816	0.096864227	Up
Solyc08g076860.3	1.941721336	0.012038617	0.045975323	Up
Solyc08g077440.3	1.638604021	0.03430013	0.090753597	Up
Solyc08g077530.3	1.17797569	0.049319475	0.115583723	Up
Solyc08g078020.1	-1.802546179	0.000916347	0.010279837	Down
Solyc08g078650.3	-1.495259698	0.004566798	0.025425052	Down
Solyc08g078670.2	2.204322134	0.01685383	0.056859419	Up
Solyc08g079740.3	-5.357552005	0.004680601	0.025789748	Down
Solyc08g080150.1	-1.451200996	0.002660652	0.018420886	Down
Solyc08g080750.3	-1.59482953	0.006173968	0.030233977	Down
Solyc08g081220.1	-4.574395166	7.08309E-05	0.00294079	Down
Solyc08g081230.1	-1.03956852	0.004379556	0.024788098	Down
Solyc08g081310.3	1.732798875	0.013257231	0.048565533	Up
Solyc08g081480.3	-3.083624264	0.000110275	0.003637654	Down
Solyc08g081610.3	-2.164906927	0.031828024	0.086379661	Down
Solyc08g081690.3	-2.295210757	0.001601861	0.013840629	Down
Solyc08g081700.1	-2.072270286	0.000127282	0.003873266	Down
Solyc08g082670.3	19.8371762	0.01110885	0.043688842	Up
Solyc08g083060.3	-2.049777561	0.000369644	0.00634705	Down
Solyc08g083140.3	-2.348406872	0.000524058	0.007585504	Down
Solyc09g007770.2	-1.365575008	0.005205708	0.027379185	Down
Solyc09g008060.3	-3.125790176	0.000231973	0.005150389	Down
Solyc09g008270.3	-1.202219965	0.001526374	0.013507082	Down
Solyc09g009100.3	1.428863919	0.004722812	0.025919849	Up
Solyc09g009150.1	-1.449426225	0.005602849	0.028500158	Down
Solyc09g009420.1	-2.97758686	0.002953921	0.019626539	Down

Solyc09g011310.3	-1.287179043	0.00262654	0.018294358	Down
Solyc09g014520.3	-1.319521382	0.000211279	0.004918946	Down
Solyc09g014900.3	2.415578718	0.004359865	0.024717177	Up
Solyc09g015700.3	4.48329554	1.09711E-05	0.001447843	Up
Solyc09g018160.3	1.107518636	0.000832319	0.009773649	Up
Solyc09g018850.3	1.696251921	0.012063569	0.046050216	Up
Solyc09g031820.1	2.454565863	0.010987209	0.043393765	Up
Solyc09g050050.1	1.602344579	0.001699754	0.014267058	Up
Solyc09g055950.1	1.597238981	0.038586843	0.097986185	Up
Solyc09g056360.3	-2.03079387	6.01573E-05	0.002805223	Down
Solyc09g059170.2	-2.440853791	0.01296426	0.047891108	Down
Solyc09g061840.3	-2.809663884	0.000444561	0.006952251	Down
Solyc09g063070.3	1.961719606	0.009034353	0.038577894	Up
Solyc09g064860.3	1.989561788	0.009106192	0.038791296	Up
Solyc09g066010.3	2.915259636	0.013969277	0.050226483	Up
Solyc09g074050.3	-1.748289366	0.001622317	0.013945781	Down
Solyc09g082780.3	-1.094682667	0.006038161	0.029771838	Down
Solyc09g083000.3	1.452773079	0.003036914	0.019952458	Up
Solyc09g083050.3	-1.468219636	0.013908406	0.050070262	Down
Solyc09g083200.3	2.713583668	0.005938608	0.029494632	Up
Solyc09g083390.2	2.914684105	0.003622499	0.022151841	Up
Solyc09g089670.3	-1.077933513	0.007803161	0.035157041	Down
Solyc09g089890.1	-1.77462372	0.001872364	0.015073318	Down
Solyc09g090070.1	-1.295609131	0.007795948	0.035149029	Down
Solyc09g090200.3	-2.375240515	0.035585012	0.09302091	Down
Solyc09g090270.3	1.061625903	0.03677863	0.095150635	Up
Solyc09g090680.3	-1.52502753	0.005974128	0.029602833	Down
Solyc09g090730.2	-1.511181544	0.005993033	0.0296454	Down
Solyc09g091090.2	-2.479690458	0.006269084	0.030532127	Down
Solyc09g091370.3	-1.905454363	0.000178772	0.004537898	Down
Solyc09g092550.3	1.598310996	0.029147001	0.081127335	Up
Solyc10g005000.3	-1.891170137	0.010368225	0.041973762	Down
Solyc10g005510.3	-2.71646122	3.3864E-06	0.000910024	Down
Solyc10g009360.3	1.851924136	0.001014392	0.010773292	Up
Solyc10g017890.1	3.850815341	0.025132867	0.07373403	Up
Solyc10g018520.1	1.637016561	0.038591637	0.097988738	Up
Solyc10g018780.2	1.68461377	0.039922179	0.100196196	Up
Solyc10g018907.1	4.973022185	0.00479156	0.026147358	Up
Solyc10g039230.1	2.670294555	0.014123783	0.050592131	Up
Solyc10g045240.2	-6.074233757	0.000766018	0.009380506	Down
Solyc10g045380.2	-2.266655834	0.003050595	0.019996625	Down
Solyc10g051080.1	2.840713519	0.010920361	0.043296452	Up
Solyc10g054170.2	-17.28771238	0.026271324	0.075764754	Down
Solyc10g054440.2	1.520914014	0.046399318	0.11063786	Up
Solyc10g054570.2	-2.339857683	4.38199E-05	0.002448049	Down
Solyc10g054910.1	-4.26803874	0.008271355	0.036534644	Down
Solyc10g055680.1	-3.657894023	0.000153882	0.004234375	Down
Solyc10g074730.3	-3.559427409	0.013683631	0.049585431	Down
Solyc10g078920.2	3.25047129	0.002937914	0.019580545	Up
Solyc10g079350.2	-3.341484335	0.00014324	0.004098504	Down

Solyc10g079620.2	2.231469887	0.017689788	0.058647022	Up
Solyc10g079640.2	-2.014183481	0.009162903	0.038882981	Down
Solyc10g079790.1	-2.827804598	0.006703172	0.03173792	Down
Solyc10g080900.2	-3.440864605	0.000121914	0.003799323	Down
Solyc10g080940.2	-1.416608635	0.002967834	0.019670326	Down
Solyc10g081320.1	-3.028635868	0.036934622	0.095382721	Down
Solyc10g083300.2	-1.73075663	0.010496449	0.04232372	Down
Solyc10g084370.2	2.404390255	0.00650089	0.031133498	Up
Solyc10g084970.2	3.961913806	0.008156547	0.036244159	Up
Solyc10g085460.2	2.684197111	0.007007814	0.032648434	Up
Solyc10g085800.2	-5.662139436	2.84839E-07	0.000414752	Down
Solyc10g086520.2	-5.519930383	0.000230174	0.005136901	Down
Solyc11g005700.1	-1.541939007	0.000833568	0.009775099	Down
Solyc11g006710.2	1.65073649	0.023092081	0.069843283	Up
Solyc11g008200.1	-3.290677161	0.001309216	0.01241356	Down
Solyc11g009050.2	2.456123718	0.012120398	0.046232041	Up
Solyc11g010120.2	2.412278623	0.002644838	0.018360545	Up
Solyc11g010430.2	1.966907362	0.046260166	0.110475991	Up
Solyc11g010500.1	-1.850633094	0.000150272	0.004193947	Down
Solyc11g011380.2	-1.196145681	7.35299E-05	0.003003856	Down
Solyc11g012320.2	-5.510589659	0.006398618	0.030924265	Down
Solyc11g013170.2	-1.033560524	0.000686523	0.008876406	Down
Solyc11g013250.1	2.689515603	0.003003908	0.019808317	Up
Solyc11g013450.2	-1.930291028	0.000125813	0.003860346	Down
Solyc11g017000.2	1.991671016	0.014012631	0.050323649	Up
Solyc11g021210.1	3.528773654	0.000380382	0.006437196	Up
Solyc11g045150.1	-2.957432828	0.008103052	0.03611414	Down
Solyc11g051170.2	-2.147266983	0.030351997	0.083528541	Down
Solyc11g056270.2	1.669108507	0.041217556	0.102522675	Up
Solyc11g056310.1	2.499304247	0.035628341	0.093092673	Up
Solyc11g056370.1	-2.284956461	0.024061258	0.071654429	Down
Solyc11g062440.2	-1.650199266	0.015039671	0.052719358	Down
Solyc11g065530.1	-6.534584524	0.000234038	0.005169611	Down
Solyc11g065830.2	-1.82317251	7.62699E-05	0.003067521	Down
Solyc11g066020.2	1.106258687	0.000162107	0.004375103	Up
Solyc11g066060.2	-1.219669351	0.000421761	0.006773724	Down
Solyc11g066580.2	-2.25955944	0.007484627	0.034313606	Down
Solyc11g066950.2	-3.762069012	0.000267051	0.005460516	Down
Solyc11g067080.2	2.340892003	0.001518227	0.013481109	Up
Solyc11g069030.1	1.975492298	0.016083751	0.055043966	Up
Solyc11g069390.1	-2.161125614	0.0139846	0.050260618	Down
Solyc12g005640.2	3.028493916	0.040497358	0.101276677	Up
Solyc12g005660.2	3.083698118	0.008948307	0.038333372	Up
Solyc12g007210.2	1.806455953	0.013824476	0.049865277	Up
Solyc12g009520.2	-2.354228587	0.004160341	0.024041862	Down
Solyc12g009790.2	-1.029316409	0.004063141	0.023675872	Down
Solyc12g010540.1	-3.563716581	0.000976635	0.010578581	Down
Solyc12g013895.1	-25.56325494	0.000634663	0.008466319	Down
Solyc12g026470.2	1.214197855	0.0341733	0.090538419	Up
Solyc12g027850.2	1.018790622	0.000962245	0.010513372	Up

Solyc12g044670.1	-21.64232909	0.005557259	0.028368671	Down
Solyc12g049500.2	-2.459431619	0.006332206	0.030735379	Down
Solyc12g049560.2	1.33598043	0.002568358	0.018027993	Up
Solyc12g056510.2	-3.491523779	1.0945E-05	0.001447843	Down
Solyc12g056620.2	-2.535331733	0.044480562	0.107717836	Down
Solyc12g088660.2	-1.575093157	0.000921627	0.010286765	Down
Solyc12g088670.2	1.238598666	0.000753301	0.009291788	Up
Solyc12g088680.2	-1.356328192	0.005372586	0.027877092	Down
Solyc12g088760.1	-3.139323959	0.0001991	0.004772858	Down
Solyc12g095980.2	-2.311291555	0.005913048	0.02942952	Down
Solyc12g098620.2	3.368151112	0.017293772	0.057860652	Up
Solyc12g099780.2	-8.443288927	0.003946755	0.023323453	Down

fold_enrich	name	gene
3.29369	IP_OE_9_vs_In_OE_9_peak_1	Solyc00g006020.1
2.12932	IP_OE_9_vs_In_OE_9_peak_2	Solyc00g005907.1
2.49887	IP_OE_9_vs_In_OE_9_peak_3	Solyc00g006680.2
2.98364	IP_OE_9_vs_In_OE_9_peak_4	Solyc00g008570.2
2.36627	IP_OE_9_vs_In_OE_9_peak_5	Solyc00g008580.1
2.26576	IP_OE_9_vs_In_OE_9_peak_6	Solyc00g009760.2
4.20548	IP_OE_9_vs_In_OE_9_peak_7	Solyc00g010530.1
2.30091	IP_OE_9_vs_In_OE_9_peak_8	Solyc00g011150.1
2.89571	IP_OE_9_vs_In_OE_9_peak_9	Solyc00g013140.2
2.88081	IP_OE_9_vs_In_OE_9_peak_10	Solyc00g013160.2
2.37992	IP_OE_9_vs_In_OE_9_peak_11	Solyc00g013170.1
2.14656	IP_OE_9_vs_In_OE_9_peak_12	Solyc00g013180.1
2.29131	IP_OE_9_vs_In_OE_9_peak_13	Solyc00g014790.2
2.63654	IP_OE_9_vs_In_OE_9_peak_14	Solyc00g014800.1
2.48856	IP_OE_9_vs_In_OE_9_peak_15	Solyc00g014820.2
2.50539	IP_OE_9_vs_In_OE_9_peak_16	Solyc00g014830.3
2.19745	IP_OE_9_vs_In_OE_9_peak_17	Solyc00g014850.1
2.43882	IP_OE_9_vs_In_OE_9_peak_18	Solyc00g014860.1
2.45839	IP_OE_9_vs_In_OE_9_peak_19	Solyc00g019630.2
3.48878	IP_OE_9_vs_In_OE_9_peak_20	Solyc00g019730.2
2.39406	IP_OE_9_vs_In_OE_9_peak_21	Solyc00g019740.1
2.30164	IP_OE_9_vs_In_OE_9_peak_22	Solyc00g019750.1
2.52176	IP_OE_9_vs_In_OE_9_peak_23	Solyc00g019970.2
2.32401	IP_OE_9_vs_In_OE_9_peak_24	Solyc00g019980.2
1.89558	IP_OE_9_vs_In_OE_9_peak_25	Solyc00g019990.1
3.21684	IP_OE_9_vs_In_OE_9_peak_26	Solyc00g020020.2
2.73696	IP_OE_9_vs_In_OE_9_peak_27	Solyc00g020030.1
2.57836	IP_OE_9_vs_In_OE_9_peak_28	Solyc00g020040.1
2.13416	IP_OE_9_vs_In_OE_9_peak_29	Solyc00g021630.1
2.47411	IP_OE_9_vs_In_OE_9_peak_30	Solyc00g021640.3
2.09437	IP_OE_9_vs_In_OE_9_peak_31	Solyc00g021650.1
2.47578	IP_OE_9_vs_In_OE_9_peak_32	Solyc00g022070.1
2.6556	IP_OE_9_vs_In_OE_9_peak_33	Solyc00g022080.1
1.97799	IP_OE_9_vs_In_OE_9_peak_34	Solyc00g022090.2
2.23655	IP_OE_9_vs_In_OE_9_peak_35	Solyc00g022103.1
2.18394	IP_OE_9_vs_In_OE_9_peak_36	Solyc00g023050.2
2.90382	IP_OE_9_vs_In_OE_9_peak_37	Solyc00g023570.1
2.19373	IP_OE_9_vs_In_OE_9_peak_38	Solyc00g023580.1
1.99467	IP_OE_9_vs_In_OE_9_peak_39	Solyc00g023590.2
2.31903	IP_OE_9_vs_In_OE_9_peak_40	Solyc00g023600.2
1.90574	IP_OE_9_vs_In_OE_9_peak_41	Solyc00g023700.2
1.88369	IP_OE_9_vs_In_OE_9_peak_42	Solyc00g026860.1
2.26128	IP_OE_9_vs_In_OE_9_peak_43	Solyc00g026870.1
1.93198	IP_OE_9_vs_In_OE_9_peak_44	Solyc00g028540.2
2.29308	IP_OE_9_vs_In_OE_9_peak_45	Solyc00g028560.1

2.16562	IP_OE_9_vs_In_OE_9_peak_46	Solyc00g030210.2
2.20645	IP_OE_9_vs_In_OE_9_peak_47	Solyc00g030820.1
2.06404	IP_OE_9_vs_In_OE_9_peak_48	Solyc00g031383.1
2.00497	IP_OE_9_vs_In_OE_9_peak_49	Solyc00g031700.2
2.3808	IP_OE_9_vs_In_OE_9_peak_50	Solyc00g036530.1
2.29753	IP_OE_9_vs_In_OE_9_peak_51	Solyc00g042130.2
2.28685	IP_OE_9_vs_In_OE_9_peak_52	Solyc00g042140.1
1.76723	IP_OE_9_vs_In_OE_9_peak_53	Solyc00g042640.1
2.06504	IP_OE_9_vs_In_OE_9_peak_54	Solyc00g044550.1
2.30178	IP_OE_9_vs_In_OE_9_peak_55	Solyc00g052540.1
2.59448	IP_OE_9_vs_In_OE_9_peak_56	Solyc00g055950.1
2.28862	IP_OE_9_vs_In_OE_9_peak_57	Solyc00g068970.2
2.20821	IP_OE_9_vs_In_OE_9_peak_58	Solyc00g069880.2
5.58267	IP_OE_9_vs_In_OE_9_peak_59	Solyc00g088210.2
1.81096	IP_OE_9_vs_In_OE_9_peak_60	Solyc06g024210.2
2.41935	IP_OE_9_vs_In_OE_9_peak_61	Solyc00g090110.1
1.9704	IP_OE_9_vs_In_OE_9_peak_62	Solyc00g090130.2
1.79861	IP_OE_9_vs_In_OE_9_peak_63	Solyc00g094530.1
2.21637	IP_OE_9_vs_In_OE_9_peak_64	Solyc00g094540.1
2.1021	IP_OE_9_vs_In_OE_9_peak_65	Solyc00g094550.1
2.04545	IP_OE_9_vs_In_OE_9_peak_66	Solyc00g097960.2
30.16571	IP_OE_9_vs_In_OE_9_peak_67	Solyc00g112190.2
27.06042	IP_OE_9_vs_In_OE_9_peak_68	Solyc00g112500.2
15.08285	IP_OE_9_vs_In_OE_9_peak_69	Solyc00g112810.2
7.98504	IP_OE_9_vs_In_OE_9_peak_70	Solyc00g117653.1
35.93268	IP_OE_9_vs_In_OE_9_peak_71	Solyc00g118680.2
28.39125	IP_OE_9_vs_In_OE_9_peak_72	Solyc00g121230.2
5.32336	IP_OE_9_vs_In_OE_9_peak_73	Solyc00g125260.2
10.64672	IP_OE_9_vs_In_OE_9_peak_74	Solyc00g127560.2
22.62428	IP_OE_9_vs_In_OE_9_peak_75	Solyc00g130290.1
3.71092	IP_OE_9_vs_In_OE_9_peak_76	Solyc00g133710.2
7.98504	IP_OE_9_vs_In_OE_9_peak_77	Solyc00g142160.1
4.87975	IP_OE_9_vs_In_OE_9_peak_78	Solyc00g142170.3
1.68672	IP_OE_9_vs_In_OE_9_peak_79	Solyc00g164580.1
23.65827	IP_OE_9_vs_In_OE_9_peak_80	Solyc00g164680.2
11.14684	IP_OE_9_vs_In_OE_9_peak_81	Solyc00g179240.2
4.12324	IP_OE_9_vs_In_OE_9_peak_82	Solyc00g181250.2
2.09315	IP_OE_9_vs_In_OE_9_peak_83	Solyc00g183050.2
2.08553	IP_OE_9_vs_In_OE_9_peak_84	Solyc00g203660.2
2.27845	IP_OE_9_vs_In_OE_9_peak_85	Solyc00g207960.1
1.8917	IP_OE_9_vs_In_OE_9_peak_86	Solyc00g227860.1
9.89011	IP_OE_9_vs_In_OE_9_peak_87	Solyc00g230070.1
1.9069	IP_OE_9_vs_In_OE_9_peak_88	Solyc00g230080.1
2.9014	IP_OE_9_vs_In_OE_9_peak_89	Solyc00g245200.2
1.79303	IP_OE_9_vs_In_OE_9_peak_90	Solyc00g249910.2
31.05293	IP_OE_9_vs_In_OE_9_peak_91	Solyc00g256710.2
2.80445	IP_OE_9_vs_In_OE_9_peak_92	Solyc00g265510.2

7.09781	IP_OE_9_vs_In_OE_9_peak_93	Solyc00g313030.1
39.03798	IP_OE_9_vs_In_OE_9_peak_94	Solyc00g320430.1
1.91168	IP_OE_9_vs_In_OE_9_peak_95	Solyc01g005120.3
2.24079	IP_OE_9_vs_In_OE_9_peak_96	Solyc01g005420.2
1.84937	IP_OE_9_vs_In_OE_9_peak_97	Solyc01g006195.1
6.6542	IP_OE_9_vs_In_OE_9_peak_98	Solyc01g006320.3
2.06551	IP_OE_9_vs_In_OE_9_peak_99	Solyc01g006390.2
2.21828	IP_OE_9_vs_In_OE_9_peak_100	Solyc01g006430.3
1.95624	IP_OE_9_vs_In_OE_9_peak_101	Solyc01g006450.3
2.57441	IP_OE_9_vs_In_OE_9_peak_102	Solyc01g006540.3
24.36566	IP_OE_9_vs_In_OE_9_peak_103	Solyc01g006825.1
8.42865	IP_OE_9_vs_In_OE_9_peak_104	Solyc01g007080.3
3.53328	IP_OE_9_vs_In_OE_9_peak_105	Solyc01g007120.3
2.30093	IP_OE_9_vs_In_OE_9_peak_106	Solyc01g008115.1
4.87975	IP_OE_9_vs_In_OE_9_peak_107	Solyc01g008140.3
6.6542	IP_OE_9_vs_In_OE_9_peak_108	Solyc01g008560.3
83.39931	IP_OE_9_vs_In_OE_9_peak_109	Solyc01g008770.3
4.43613	IP_OE_9_vs_In_OE_9_peak_110	Solyc01g008790.3
2.61639	IP_OE_9_vs_In_OE_9_peak_111	Solyc01g009730.2
3.16891	IP_OE_9_vs_In_OE_9_peak_112	Solyc01g009860.3
2.25995	IP_OE_9_vs_In_OE_9_peak_113	Solyc01g010135.1
2.37498	IP_OE_9_vs_In_OE_9_peak_114	Solyc01g010660.3
2.75579	IP_OE_9_vs_In_OE_9_peak_115	Solyc01g010910.2
2.79587	IP_OE_9_vs_In_OE_9_peak_116	Solyc01g010980.2
3.57648	IP_OE_9_vs_In_OE_9_peak_117	Solyc01g011025.1
2.5998	IP_OE_9_vs_In_OE_9_peak_118	Solyc01g011300.1
2.22621	IP_OE_9_vs_In_OE_9_peak_119	Solyc01g013885.1
2.64714	IP_OE_9_vs_In_OE_9_peak_120	Solyc01g014060.2
4.56467	IP_OE_9_vs_In_OE_9_peak_121	Solyc01g015130.1
4.94789	IP_OE_9_vs_In_OE_9_peak_122	Solyc01g016720.2
2.24725	IP_OE_9_vs_In_OE_9_peak_123	Solyc01g016850.1
2.44597	IP_OE_9_vs_In_OE_9_peak_124	Solyc01g017220.1
4.96853	IP_OE_9_vs_In_OE_9_peak_125	Solyc01g017370.1
3.99252	IP_OE_9_vs_In_OE_9_peak_126	Solyc01g017460.2
4.87975	IP_OE_9_vs_In_OE_9_peak_127	Solyc01g017440.1
4.20942	IP_OE_9_vs_In_OE_9_peak_128	Solyc01g017540.1
2.55956	IP_OE_9_vs_In_OE_9_peak_129	Solyc01g028987.1
1.84731	IP_OE_9_vs_In_OE_9_peak_130	Solyc01g028860.3
3.99252	IP_OE_9_vs_In_OE_9_peak_131	Solyc01g044240.3
3.99252	IP_OE_9_vs_In_OE_9_peak_132	Solyc01g044340.3
4.12324	IP_OE_9_vs_In_OE_9_peak_133	Solyc01g044540.1
4.43613	IP_OE_9_vs_In_OE_9_peak_134	Solyc01g049970.2
4.23654	IP_OE_9_vs_In_OE_9_peak_135	Solyc01g050040.3
5.32336	IP_OE_9_vs_In_OE_9_peak_136	Solyc01g056200.1
4.12324	IP_OE_9_vs_In_OE_9_peak_137	Solyc01g056870.2
4.87975	IP_OE_9_vs_In_OE_9_peak_138	Solyc01g057080.1
4.23654	IP_OE_9_vs_In_OE_9_peak_139	Solyc01g057680.3

3.99252	IP_OE_9_vs_In_OE_9_peak_140	Solyc01g057780.3
5.32336	IP_OE_9_vs_In_OE_9_peak_141	Solyc01g058360.1
4.53556	IP_OE_9_vs_In_OE_9_peak_142	Solyc01g058500.3
4.36697	IP_OE_9_vs_In_OE_9_peak_143	Solyc01g059800.1
4.781	IP_OE_9_vs_In_OE_9_peak_144	Solyc01g060085.1
4.43613	IP_OE_9_vs_In_OE_9_peak_145	Solyc01g060170.3
4.87975	IP_OE_9_vs_In_OE_9_peak_146	Solyc01g065495.1
4.43613	IP_OE_9_vs_In_OE_9_peak_147	Solyc01g066200.1
5.32336	IP_OE_9_vs_In_OE_9_peak_148	Solyc01g066430.3
5.76697	IP_OE_9_vs_In_OE_9_peak_149	Solyc01g067130.3
4.94506	IP_OE_9_vs_In_OE_9_peak_150	Solyc01g067660.3
4.94506	IP_OE_9_vs_In_OE_9_peak_151	Solyc01g067740.3
4.18428	IP_OE_9_vs_In_OE_9_peak_152	Solyc01g068250.2
6.21059	IP_OE_9_vs_In_OE_9_peak_153	Solyc01g068430.2
4.69729	IP_OE_9_vs_In_OE_9_peak_154	Solyc01g079200.3
3.99252	IP_OE_9_vs_In_OE_9_peak_155	Solyc01g079400.3
4.58959	IP_OE_9_vs_In_OE_9_peak_156	Solyc01g079410.3
3.63584	IP_OE_9_vs_In_OE_9_peak_157	Solyc01g079570.3
4.43613	IP_OE_9_vs_In_OE_9_peak_158	Solyc01g079580.3
4.58959	IP_OE_9_vs_In_OE_9_peak_159	Solyc01g079620.3
4.87975	IP_OE_9_vs_In_OE_9_peak_160	Solyc01g079740.3
2.72497	IP_OE_9_vs_In_OE_9_peak_161	Solyc01g079880.3
2.12204	IP_OE_9_vs_In_OE_9_peak_162	Solyc01g079890.3
3.37649	IP_OE_9_vs_In_OE_9_peak_163	Solyc01g079940.3
3.38585	IP_OE_9_vs_In_OE_9_peak_164	Solyc01g080160.3
3.21858	IP_OE_9_vs_In_OE_9_peak_165	Solyc01g080500.2
4.75884	IP_OE_9_vs_In_OE_9_peak_166	Solyc01g080580.3
4.26368	IP_OE_9_vs_In_OE_9_peak_167	Solyc01g080800.3
4.56467	IP_OE_9_vs_In_OE_9_peak_168	Solyc01g081570.3
3.99252	IP_OE_9_vs_In_OE_9_peak_169	Solyc01g073660.3
4.33431	IP_OE_9_vs_In_OE_9_peak_170	Solyc01g073680.3
4.43613	IP_OE_9_vs_In_OE_9_peak_171	Solyc01g073700.3
4.59579	IP_OE_9_vs_In_OE_9_peak_172	Solyc01g073880.2
4.12324	IP_OE_9_vs_In_OE_9_peak_173	Solyc01g073900.3
27.06042	IP_OE_9_vs_In_OE_9_peak_174	Solyc01g073940.3
5.32336	IP_OE_9_vs_In_OE_9_peak_175	Solyc01g086810.2
4.93435	IP_OE_9_vs_In_OE_9_peak_176	Solyc01g086870.3
6.21059	IP_OE_9_vs_In_OE_9_peak_177	Solyc01g087170.3
3.53045	IP_OE_9_vs_In_OE_9_peak_178	Solyc01g087480.1
5.11232	IP_OE_9_vs_In_OE_9_peak_179	Solyc01g087570.2
3.931	IP_OE_9_vs_In_OE_9_peak_180	Solyc01g087590.3
1.8758	IP_OE_9_vs_In_OE_9_peak_181	Solyc01g087600.3
3.35346	IP_OE_9_vs_In_OE_9_peak_182	Solyc01g087740.1
5.76697	IP_OE_9_vs_In_OE_9_peak_183	Solyc01g087890.3
2.59053	IP_OE_9_vs_In_OE_9_peak_184	Solyc01g087970.3
5.69853	IP_OE_9_vs_In_OE_9_peak_185	Solyc01g088040.3
7.94103	IP_OE_9_vs_In_OE_9_peak_186	Solyc01g088100.3

3.70402	IP_OE_9_vs_In_OE_9_peak_187	Solyc01g088320.2
7.42183	IP_OE_9_vs_In_OE_9_peak_188	Solyc01g088390.3
4.53556	IP_OE_9_vs_In_OE_9_peak_189	Solyc01g088400.3
4.43613	IP_OE_9_vs_In_OE_9_peak_190	Solyc01g088660.3
6.44095	IP_OE_9_vs_In_OE_9_peak_191	Solyc01g088670.2
4.18428	IP_OE_9_vs_In_OE_9_peak_192	Solyc01g088770.3
4.43613	IP_OE_9_vs_In_OE_9_peak_193	Solyc01g089880.3
3.99252	IP_OE_9_vs_In_OE_9_peak_194	Solyc01g090230.3
3.98416	IP_OE_9_vs_In_OE_9_peak_195	Solyc01g090460.3
5.32055	IP_OE_9_vs_In_OE_9_peak_196	Solyc01g090730.3
3.96765	IP_OE_9_vs_In_OE_9_peak_197	Solyc01g090790.3
4.43613	IP_OE_9_vs_In_OE_9_peak_198	Solyc01g090800.3
3.54891	IP_OE_9_vs_In_OE_9_peak_199	Solyc01g091000.3
4.12324	IP_OE_9_vs_In_OE_9_peak_200	Solyc01g091360.3
4.56467	IP_OE_9_vs_In_OE_9_peak_201	Solyc01g091370.3
6.21059	IP_OE_9_vs_In_OE_9_peak_202	Solyc01g091480.3
4.06583	IP_OE_9_vs_In_OE_9_peak_203	Solyc01g091490.3
6.21059	IP_OE_9_vs_In_OE_9_peak_204	Solyc01g091620.3
4.43613	IP_OE_9_vs_In_OE_9_peak_205	Solyc01g091630.3
4.94506	IP_OE_9_vs_In_OE_9_peak_206	Solyc01g091655.1
3.99252	IP_OE_9_vs_In_OE_9_peak_207	Solyc01g092950.3
5.77253	IP_OE_9_vs_In_OE_9_peak_208	Solyc01g094010.3
4.53556	IP_OE_9_vs_In_OE_9_peak_209	Solyc01g094250.3
4.53556	IP_OE_9_vs_In_OE_9_peak_210	Solyc01g094360.3
4.53556	IP_OE_9_vs_In_OE_9_peak_211	Solyc01g094680.3
3.53352	IP_OE_9_vs_In_OE_9_peak_212	Solyc01g094690.3
3.54891	IP_OE_9_vs_In_OE_9_peak_213	Solyc01g094720.3
4.74114	IP_OE_9_vs_In_OE_9_peak_214	Solyc01g094750.3
5.32336	IP_OE_9_vs_In_OE_9_peak_215	Solyc01g094820.3
4.43613	IP_OE_9_vs_In_OE_9_peak_216	Solyc01g094960.3
6.08076	IP_OE_9_vs_In_OE_9_peak_217	Solyc01g095070.3
4.43613	IP_OE_9_vs_In_OE_9_peak_218	Solyc01g095180.2
4.58959	IP_OE_9_vs_In_OE_9_peak_219	Solyc01g095450.3
4.87975	IP_OE_9_vs_In_OE_9_peak_220	Solyc01g095530.2
3.29369	IP_OE_9_vs_In_OE_9_peak_221	Solyc01g095620.3
4.94789	IP_OE_9_vs_In_OE_9_peak_222	Solyc01g095640.2
3.62306	IP_OE_9_vs_In_OE_9_peak_223	Solyc01g096060.3
3.99252	IP_OE_9_vs_In_OE_9_peak_224	Solyc01g096070.3
3.4235	IP_OE_9_vs_In_OE_9_peak_225	Solyc01g096080.3
3.95242	IP_OE_9_vs_In_OE_9_peak_226	Solyc01g096200.3
4.20305	IP_OE_9_vs_In_OE_9_peak_227	Solyc01g096350.3
6.6542	IP_OE_9_vs_In_OE_9_peak_228	Solyc01g096620.3
7.09781	IP_OE_9_vs_In_OE_9_peak_229	Solyc01g096660.3
3.99252	IP_OE_9_vs_In_OE_9_peak_230	Solyc01g096680.3
4.43613	IP_OE_9_vs_In_OE_9_peak_231	Solyc01g096940.3
5.32336	IP_OE_9_vs_In_OE_9_peak_232	Solyc01g097280.2
5.26999	IP_OE_9_vs_In_OE_9_peak_233	Solyc01g097330.3

3.95242	IP_OE_9_vs_In_OE_9_peak_234	Solyc01g097340.3
5.70933	IP_OE_9_vs_In_OE_9_peak_235	Solyc01g097395.1
6.0567	IP_OE_9_vs_In_OE_9_peak_236	Solyc01g097500.3
5.76697	IP_OE_9_vs_In_OE_9_peak_237	Solyc01g097890.2
5.26177	IP_OE_9_vs_In_OE_9_peak_238	Solyc01g098080.3
5.26999	IP_OE_9_vs_In_OE_9_peak_239	Solyc01g098390.3
5.70583	IP_OE_9_vs_In_OE_9_peak_240	Solyc01g098590.3
4.87975	IP_OE_9_vs_In_OE_9_peak_241	Solyc01g098725.1
4.87975	IP_OE_9_vs_In_OE_9_peak_242	Solyc01g098730.2
4.43613	IP_OE_9_vs_In_OE_9_peak_243	Solyc01g098780.3
5.09189	IP_OE_9_vs_In_OE_9_peak_244	Solyc01g098890.1
3.87192	IP_OE_9_vs_In_OE_9_peak_245	Solyc01g099160.3
7.70095	IP_OE_9_vs_In_OE_9_peak_246	Solyc01g099260.3
4.56467	IP_OE_9_vs_In_OE_9_peak_247	Solyc01g099330.3
3.80389	IP_OE_9_vs_In_OE_9_peak_248	Solyc01g099340.3
6.72124	IP_OE_9_vs_In_OE_9_peak_249	Solyc01g099630.3
3.80389	IP_OE_9_vs_In_OE_9_peak_250	Solyc01g099780.3
5.93486	IP_OE_9_vs_In_OE_9_peak_251	Solyc01g099880.3
5.51094	IP_OE_9_vs_In_OE_9_peak_252	Solyc01g100040.3
6.92195	IP_OE_9_vs_In_OE_9_peak_253	Solyc01g100045.1
3.99252	IP_OE_9_vs_In_OE_9_peak_254	Solyc01g100050.3
5.35917	IP_OE_9_vs_In_OE_9_peak_255	Solyc01g100310.2
4.01269	IP_OE_9_vs_In_OE_9_peak_256	Solyc01g100530.3
7.09781	IP_OE_9_vs_In_OE_9_peak_257	Solyc01g100980.3
6.54798	IP_OE_9_vs_In_OE_9_peak_258	Solyc01g101060.3
4.43613	IP_OE_9_vs_In_OE_9_peak_259	Solyc01g102270.2
3.71092	IP_OE_9_vs_In_OE_9_peak_260	Solyc01g102280.3
6.21059	IP_OE_9_vs_In_OE_9_peak_261	Solyc01g102290.3
3.99252	IP_OE_9_vs_In_OE_9_peak_262	Solyc01g102340.3
6.46661	IP_OE_9_vs_In_OE_9_peak_263	Solyc01g102350.3
5.32336	IP_OE_9_vs_In_OE_9_peak_264	Solyc01g102360.2
3.485	IP_OE_9_vs_In_OE_9_peak_265	Solyc01g102390.3
3.99041	IP_OE_9_vs_In_OE_9_peak_266	Solyc01g102410.3
4.43613	IP_OE_9_vs_In_OE_9_peak_267	Solyc01g102580.3
3.29369	IP_OE_9_vs_In_OE_9_peak_268	Solyc01g102730.3
3.99252	IP_OE_9_vs_In_OE_9_peak_269	Solyc01g102850.2
4.12324	IP_OE_9_vs_In_OE_9_peak_270	Solyc01g103430.3
5.13397	IP_OE_9_vs_In_OE_9_peak_271	Solyc01g103590.3
4.12324	IP_OE_9_vs_In_OE_9_peak_272	Solyc01g104015.1
3.50017	IP_OE_9_vs_In_OE_9_peak_273	Solyc01g104090.3
4.87975	IP_OE_9_vs_In_OE_9_peak_274	Solyc01g104370.3
4.62085	IP_OE_9_vs_In_OE_9_peak_275	Solyc01g104710.3
5.32336	IP_OE_9_vs_In_OE_9_peak_276	Solyc01g104950.3
6.10226	IP_OE_9_vs_In_OE_9_peak_277	Solyc01g105010.3
5.76697	IP_OE_9_vs_In_OE_9_peak_278	Solyc01g105190.3
5.76697	IP_OE_9_vs_In_OE_9_peak_279	Solyc01g105360.3
4.23654	IP_OE_9_vs_In_OE_9_peak_280	Solyc01g105410.3

7.09781	IP_OE_9_vs_In_OE_9_peak_281	Solyc01g105497.1
4.43613	IP_OE_9_vs_In_OE_9_peak_282	Solyc01g105890.3
3.53045	IP_OE_9_vs_In_OE_9_peak_283	Solyc01g106270.1
5.32336	IP_OE_9_vs_In_OE_9_peak_284	Solyc01g106320.2
9.31588	IP_OE_9_vs_In_OE_9_peak_285	Solyc01g106910.3
5.76697	IP_OE_9_vs_In_OE_9_peak_286	Solyc01g107190.3
4.43379	IP_OE_9_vs_In_OE_9_peak_287	Solyc01g107350.3
4.56467	IP_OE_9_vs_In_OE_9_peak_288	Solyc01g107590.3
5.26999	IP_OE_9_vs_In_OE_9_peak_289	Solyc01g107600.3
5.57783	IP_OE_9_vs_In_OE_9_peak_290	Solyc01g107650.3
4.53556	IP_OE_9_vs_In_OE_9_peak_291	Solyc01g107720.3
5.70583	IP_OE_9_vs_In_OE_9_peak_292	Solyc01g107740.3
4.43613	IP_OE_9_vs_In_OE_9_peak_293	Solyc01g107800.3
5.26999	IP_OE_9_vs_In_OE_9_peak_294	Solyc01g107810.2
6.6542	IP_OE_9_vs_In_OE_9_peak_295	Solyc01g107820.2
4.4053	IP_OE_9_vs_In_OE_9_peak_296	Solyc01g107890.3
4.61116	IP_OE_9_vs_In_OE_9_peak_297	Solyc01g107980.3
5.32336	IP_OE_9_vs_In_OE_9_peak_298	Solyc01g107990.3
5.68319	IP_OE_9_vs_In_OE_9_peak_299	Solyc01g108087.1
4.18428	IP_OE_9_vs_In_OE_9_peak_300	Solyc01g108230.3
3.99252	IP_OE_9_vs_In_OE_9_peak_301	Solyc01g108300.3
4.12324	IP_OE_9_vs_In_OE_9_peak_302	Solyc01g108440.2
5.76697	IP_OE_9_vs_In_OE_9_peak_303	Solyc01g108520.2
6.21059	IP_OE_9_vs_In_OE_9_peak_304	Solyc01g108540.3
5.23051	IP_OE_9_vs_In_OE_9_peak_305	Solyc01g108610.3
5.76697	IP_OE_9_vs_In_OE_9_peak_306	Solyc01g109085.1
4.87975	IP_OE_9_vs_In_OE_9_peak_307	Solyc01g109240.2
4.87975	IP_OE_9_vs_In_OE_9_peak_308	Solyc01g109380.3
5.45376	IP_OE_9_vs_In_OE_9_peak_309	Solyc01g109530.3
8.42865	IP_OE_9_vs_In_OE_9_peak_310	Solyc01g109560.3
5.34477	IP_OE_9_vs_In_OE_9_peak_311	Solyc01g109650.3
7.98504	IP_OE_9_vs_In_OE_9_peak_312	Solyc01g109660.2
3.91684	IP_OE_9_vs_In_OE_9_peak_313	Solyc01g109655.1
5.17215	IP_OE_9_vs_In_OE_9_peak_314	Solyc01g109700.3
4.74997	IP_OE_9_vs_In_OE_9_peak_315	Solyc01g110130.3
4.43613	IP_OE_9_vs_In_OE_9_peak_316	Solyc01g110310.3
4.43613	IP_OE_9_vs_In_OE_9_peak_317	Solyc01g110380.3
4.18428	IP_OE_9_vs_In_OE_9_peak_318	Solyc01g110460.3
4.87975	IP_OE_9_vs_In_OE_9_peak_319	Solyc01g110480.3
5.3638	IP_OE_9_vs_In_OE_9_peak_320	Solyc01g110520.3
4.58959	IP_OE_9_vs_In_OE_9_peak_321	Solyc01g110900.1
3.80389	IP_OE_9_vs_In_OE_9_peak_322	Solyc01g110920.3
4.74997	IP_OE_9_vs_In_OE_9_peak_323	Solyc01g111145.1
4.27831	IP_OE_9_vs_In_OE_9_peak_324	Solyc01g111170.3
6.55045	IP_OE_9_vs_In_OE_9_peak_325	Solyc01g111300.3
7.60778	IP_OE_9_vs_In_OE_9_peak_326	Solyc01g111330.3
4.94789	IP_OE_9_vs_In_OE_9_peak_327	Solyc01g111360.3

5.32336	IP_OE_9_vs_In_OE_9_peak_328	Solyc01g111500.3
3.29369	IP_OE_9_vs_In_OE_9_peak_329	Solyc01g111560.3
4.43613	IP_OE_9_vs_In_OE_9_peak_330	Solyc01g111830.3
3.95242	IP_OE_9_vs_In_OE_9_peak_331	Solyc01g111980.3
3.51343	IP_OE_9_vs_In_OE_9_peak_332	Solyc01g112080.3
4.18428	IP_OE_9_vs_In_OE_9_peak_333	Solyc01g112150.3
4.87975	IP_OE_9_vs_In_OE_9_peak_334	Solyc02g011800.1
5.22069	IP_OE_9_vs_In_OE_9_peak_335	Solyc02g011815.1
5.32336	IP_OE_9_vs_In_OE_9_peak_336	Solyc02g011990.1
6.6542	IP_OE_9_vs_In_OE_9_peak_337	Solyc02g012000.1
5.1057	IP_OE_9_vs_In_OE_9_peak_338	Solyc02g014030.2
5.36021	IP_OE_9_vs_In_OE_9_peak_339	Solyc02g014130.2
5.36021	IP_OE_9_vs_In_OE_9_peak_340	Solyc02g022850.1
3.64846	IP_OE_9_vs_In_OE_9_peak_341	Solyc02g022930.3
3.80389	IP_OE_9_vs_In_OE_9_peak_342	Solyc02g024010.1
3.96765	IP_OE_9_vs_In_OE_9_peak_343	Solyc02g024070.3
5.36021	IP_OE_9_vs_In_OE_9_peak_344	Solyc02g030080.3
7.98504	IP_OE_9_vs_In_OE_9_peak_345	Solyc02g031680.1
3.95242	IP_OE_9_vs_In_OE_9_peak_346	Solyc02g031700.2
4.18428	IP_OE_9_vs_In_OE_9_peak_347	Solyc02g032160.1
4.87975	IP_OE_9_vs_In_OE_9_peak_348	Solyc02g032650.3
6.6542	IP_OE_9_vs_In_OE_9_peak_349	Solyc02g032950.3
4.70614	IP_OE_9_vs_In_OE_9_peak_350	Solyc02g036370.3
7.54143	IP_OE_9_vs_In_OE_9_peak_351	Solyc02g037550.3
4.23654	IP_OE_9_vs_In_OE_9_peak_352	Solyc02g038720.2
4.38258	IP_OE_9_vs_In_OE_9_peak_353	Solyc02g062230.1
4.43613	IP_OE_9_vs_In_OE_9_peak_354	Solyc02g062340.3
3.62306	IP_OE_9_vs_In_OE_9_peak_355	Solyc02g062690.3
3.64846	IP_OE_9_vs_In_OE_9_peak_356	Solyc02g062710.1
7.54143	IP_OE_9_vs_In_OE_9_peak_357	Solyc02g063000.3
7.40989	IP_OE_9_vs_In_OE_9_peak_358	Solyc02g063030.3
4.58959	IP_OE_9_vs_In_OE_9_peak_359	Solyc02g063080.1
5.24736	IP_OE_9_vs_In_OE_9_peak_360	Solyc02g063150.3
2.68612	IP_OE_9_vs_In_OE_9_peak_361	Solyc02g063360.3
4.75794	IP_OE_9_vs_In_OE_9_peak_362	Solyc02g063390.3
4.02661	IP_OE_9_vs_In_OE_9_peak_363	Solyc02g063520.3
6.21059	IP_OE_9_vs_In_OE_9_peak_364	Solyc02g064680.3
4.43613	IP_OE_9_vs_In_OE_9_peak_365	Solyc02g064760.2
3.54891	IP_OE_9_vs_In_OE_9_peak_366	Solyc02g064940.1
6.51364	IP_OE_9_vs_In_OE_9_peak_367	Solyc02g065070.3
5.09189	IP_OE_9_vs_In_OE_9_peak_368	Solyc02g065280.3
5.32336	IP_OE_9_vs_In_OE_9_peak_369	Solyc02g067050.3
3.80389	IP_OE_9_vs_In_OE_9_peak_370	Solyc02g067230.3
4.87975	IP_OE_9_vs_In_OE_9_peak_371	Solyc02g067310.3
5.39246	IP_OE_9_vs_In_OE_9_peak_372	Solyc02g067350.3
4.94789	IP_OE_9_vs_In_OE_9_peak_373	Solyc02g067530.3
3.97051	IP_OE_9_vs_In_OE_9_peak_374	Solyc02g067660.3

5.76697	IP_OE_9_vs_In_OE_9_peak_375	Solyc02g067810.1
5.46101	IP_OE_9_vs_In_OE_9_peak_376	Solyc02g067890.3
4.43613	IP_OE_9_vs_In_OE_9_peak_377	Solyc02g068040.3
5.92864	IP_OE_9_vs_In_OE_9_peak_378	Solyc02g068610.2
6.6542	IP_OE_9_vs_In_OE_9_peak_379	Solyc02g068920.3
3.71092	IP_OE_9_vs_In_OE_9_peak_380	Solyc02g069100.3
5.36021	IP_OE_9_vs_In_OE_9_peak_381	Solyc02g069180.3
4.26257	IP_OE_9_vs_In_OE_9_peak_382	Solyc02g069190.3
5.66638	IP_OE_9_vs_In_OE_9_peak_383	Solyc02g069470.3
4.18428	IP_OE_9_vs_In_OE_9_peak_384	Solyc02g069580.3
5.32336	IP_OE_9_vs_In_OE_9_peak_385	Solyc02g069870.1
4.41495	IP_OE_9_vs_In_OE_9_peak_386	Solyc02g070060.1
4.87975	IP_OE_9_vs_In_OE_9_peak_387	Solyc02g070280.3
4.12324	IP_OE_9_vs_In_OE_9_peak_388	Solyc02g070290.3
4.56467	IP_OE_9_vs_In_OE_9_peak_389	Solyc02g070340.3
3.80389	IP_OE_9_vs_In_OE_9_peak_390	Solyc02g071220.3
4.12324	IP_OE_9_vs_In_OE_9_peak_391	Solyc02g071250.3
7.09781	IP_OE_9_vs_In_OE_9_peak_392	Solyc02g071610.3
4.84935	IP_OE_9_vs_In_OE_9_peak_393	Solyc02g071740.3
4.0824	IP_OE_9_vs_In_OE_9_peak_394	Solyc02g071920.3
3.07089	IP_OE_9_vs_In_OE_9_peak_395	Solyc02g071990.3
2.93032	IP_OE_9_vs_In_OE_9_peak_396	Solyc02g072080.1
3.73786	IP_OE_9_vs_In_OE_9_peak_397	Solyc02g072150.3
3.00883	IP_OE_9_vs_In_OE_9_peak_398	Solyc02g072160.3
2.71494	IP_OE_9_vs_In_OE_9_peak_399	Solyc02g072290.1
3.91642	IP_OE_9_vs_In_OE_9_peak_400	Solyc02g072310.3
2.94221	IP_OE_9_vs_In_OE_9_peak_401	Solyc02g072447.1
6.00177	IP_OE_9_vs_In_OE_9_peak_402	Solyc02g072510.3
3.88639	IP_OE_9_vs_In_OE_9_peak_403	Solyc02g072520.3
5.36021	IP_OE_9_vs_In_OE_9_peak_404	Solyc02g073570.1
6.81983	IP_OE_9_vs_In_OE_9_peak_405	Solyc02g076840.3
2.8241	IP_OE_9_vs_In_OE_9_peak_406	Solyc02g076930.2
4.94789	IP_OE_9_vs_In_OE_9_peak_407	Solyc02g077040.4
3.95242	IP_OE_9_vs_In_OE_9_peak_408	Solyc02g077050.3
6.6542	IP_OE_9_vs_In_OE_9_peak_409	Solyc02g077080.3
3.80389	IP_OE_9_vs_In_OE_9_peak_410	Solyc02g077420.3
4.43613	IP_OE_9_vs_In_OE_9_peak_411	Solyc02g077620.1
6.10226	IP_OE_9_vs_In_OE_9_peak_412	Solyc02g077710.1
6.847	IP_OE_9_vs_In_OE_9_peak_413	Solyc02g077720.3
4.43613	IP_OE_9_vs_In_OE_9_peak_414	Solyc02g077780.3
4.43613	IP_OE_9_vs_In_OE_9_peak_415	Solyc02g077860.1
4.56467	IP_OE_9_vs_In_OE_9_peak_416	Solyc02g077920.3
5.74196	IP_OE_9_vs_In_OE_9_peak_417	Solyc02g077950.2
3.99252	IP_OE_9_vs_In_OE_9_peak_418	Solyc02g078030.2
5.32336	IP_OE_9_vs_In_OE_9_peak_419	Solyc02g078040.3
4.62085	IP_OE_9_vs_In_OE_9_peak_420	Solyc02g078170.2
3.53045	IP_OE_9_vs_In_OE_9_peak_421	Solyc02g078490.3

3.95242	IP_OE_9_vs_In_OE_9_peak_422	Solyc02g078520.3
3.80389	IP_OE_9_vs_In_OE_9_peak_423	Solyc02g078560.3
5.32336	IP_OE_9_vs_In_OE_9_peak_424	Solyc02g078740.3
5.57783	IP_OE_9_vs_In_OE_9_peak_425	Solyc02g079150.2
3.10193	IP_OE_9_vs_In_OE_9_peak_426	Solyc02g079220.3
3.53894	IP_OE_9_vs_In_OE_9_peak_427	Solyc02g079280.3
4.43613	IP_OE_9_vs_In_OE_9_peak_428	Solyc02g079430.3
4.43613	IP_OE_9_vs_In_OE_9_peak_429	Solyc02g079440.2
4.79463	IP_OE_9_vs_In_OE_9_peak_430	Solyc02g079510.3
6.6542	IP_OE_9_vs_In_OE_9_peak_431	Solyc02g079570.3
6.21059	IP_OE_9_vs_In_OE_9_peak_432	Solyc02g079590.3
5.76697	IP_OE_9_vs_In_OE_9_peak_433	Solyc02g079740.1
4.87975	IP_OE_9_vs_In_OE_9_peak_434	Solyc02g079760.3
5.70583	IP_OE_9_vs_In_OE_9_peak_435	Solyc02g079890.2
4.43613	IP_OE_9_vs_In_OE_9_peak_436	Solyc02g080150.2
5.36021	IP_OE_9_vs_In_OE_9_peak_437	Solyc02g080200.3
4.20077	IP_OE_9_vs_In_OE_9_peak_438	Solyc02g080340.3
5.51094	IP_OE_9_vs_In_OE_9_peak_439	Solyc02g080450.1
4.18428	IP_OE_9_vs_In_OE_9_peak_440	Solyc02g080635.1
3.29369	IP_OE_9_vs_In_OE_9_peak_441	Solyc02g080760.1
4.53556	IP_OE_9_vs_In_OE_9_peak_442	Solyc02g080800.3
7.39979	IP_OE_9_vs_In_OE_9_peak_443	Solyc02g080890.3
5.76697	IP_OE_9_vs_In_OE_9_peak_444	Solyc02g081040.3
4.18428	IP_OE_9_vs_In_OE_9_peak_445	Solyc02g081070.3
4.87975	IP_OE_9_vs_In_OE_9_peak_446	Solyc02g081330.4
3.99252	IP_OE_9_vs_In_OE_9_peak_447	Solyc02g081390.3
6.21059	IP_OE_9_vs_In_OE_9_peak_448	Solyc02g081400.3
4.58959	IP_OE_9_vs_In_OE_9_peak_449	Solyc02g081440.3
4.94263	IP_OE_9_vs_In_OE_9_peak_450	Solyc02g081770.1
4.5386	IP_OE_9_vs_In_OE_9_peak_451	Solyc02g082035.1
4.42533	IP_OE_9_vs_In_OE_9_peak_452	Solyc02g082040.3
4.43613	IP_OE_9_vs_In_OE_9_peak_453	Solyc02g082150.1
4.01269	IP_OE_9_vs_In_OE_9_peak_454	Solyc02g082160.2
4.43613	IP_OE_9_vs_In_OE_9_peak_455	Solyc02g082550.3
8.3921	IP_OE_9_vs_In_OE_9_peak_456	Solyc02g082740.1
3.8835	IP_OE_9_vs_In_OE_9_peak_457	Solyc02g083310.3
5.36021	IP_OE_9_vs_In_OE_9_peak_458	Solyc02g083350.3
5.60317	IP_OE_9_vs_In_OE_9_peak_459	Solyc02g083410.3
4.53556	IP_OE_9_vs_In_OE_9_peak_460	Solyc02g083450.3
3.97286	IP_OE_9_vs_In_OE_9_peak_461	Solyc02g083460.3
5.32336	IP_OE_9_vs_In_OE_9_peak_462	Solyc02g083820.1
3.94514	IP_OE_9_vs_In_OE_9_peak_463	Solyc02g084420.3
6.21059	IP_OE_9_vs_In_OE_9_peak_464	Solyc02g084440.3
5.29568	IP_OE_9_vs_In_OE_9_peak_465	Solyc02g084570.3
4.43613	IP_OE_9_vs_In_OE_9_peak_466	Solyc02g084600.3
3.99252	IP_OE_9_vs_In_OE_9_peak_467	Solyc02g084720.3
5.5618	IP_OE_9_vs_In_OE_9_peak_468	Solyc02g084800.3

6.6542	IP_OE_9_vs_In_OE_9_peak_469	Solyc02g084840.3
4.23654	IP_OE_9_vs_In_OE_9_peak_470	Solyc02g084940.1
7.54143	IP_OE_9_vs_In_OE_9_peak_471	Solyc02g084950.3
5.92864	IP_OE_9_vs_In_OE_9_peak_472	Solyc02g084990.3
3.29369	IP_OE_9_vs_In_OE_9_peak_473	Solyc02g085000.3
6.00177	IP_OE_9_vs_In_OE_9_peak_474	Solyc02g085005.1
4.97778	IP_OE_9_vs_In_OE_9_peak_475	Solyc02g085010.1
5.29568	IP_OE_9_vs_In_OE_9_peak_476	Solyc02g085160.1
3.5466	IP_OE_9_vs_In_OE_9_peak_477	Solyc02g085190.2
6.85667	IP_OE_9_vs_In_OE_9_peak_478	Solyc02g085340.1
4.12324	IP_OE_9_vs_In_OE_9_peak_479	Solyc02g085360.3
6.21059	IP_OE_9_vs_In_OE_9_peak_480	Solyc02g085375.1
5.46101	IP_OE_9_vs_In_OE_9_peak_481	Solyc02g085750.3
4.92544	IP_OE_9_vs_In_OE_9_peak_482	Solyc02g085935.1
5.32336	IP_OE_9_vs_In_OE_9_peak_483	Solyc02g086130.3
4.35876	IP_OE_9_vs_In_OE_9_peak_484	Solyc02g086210.3
4.38349	IP_OE_9_vs_In_OE_9_peak_485	Solyc02g086520.3
3.62306	IP_OE_9_vs_In_OE_9_peak_486	Solyc02g086590.3
7.56991	IP_OE_9_vs_In_OE_9_peak_487	Solyc02g086650.3
4.84622	IP_OE_9_vs_In_OE_9_peak_488	Solyc02g086670.3
4.53556	IP_OE_9_vs_In_OE_9_peak_489	Solyc02g086700.3
3.99252	IP_OE_9_vs_In_OE_9_peak_490	Solyc02g086840.3
4.53556	IP_OE_9_vs_In_OE_9_peak_491	Solyc02g087030.1
6.81997	IP_OE_9_vs_In_OE_9_peak_492	Solyc02g087415.1
5.67538	IP_OE_9_vs_In_OE_9_peak_493	Solyc02g087430.3
4.61116	IP_OE_9_vs_In_OE_9_peak_494	Solyc02g087480.3
3.99252	IP_OE_9_vs_In_OE_9_peak_495	Solyc02g087500.2
5.32336	IP_OE_9_vs_In_OE_9_peak_496	Solyc02g087550.3
6.04542	IP_OE_9_vs_In_OE_9_peak_497	Solyc02g087770.3
3.39535	IP_OE_9_vs_In_OE_9_peak_498	Solyc02g087850.1
4.79463	IP_OE_9_vs_In_OE_9_peak_499	Solyc02g087980.3
6.97401	IP_OE_9_vs_In_OE_9_peak_500	Solyc02g088050.1
5.5618	IP_OE_9_vs_In_OE_9_peak_501	Solyc02g088110.3
7.09781	IP_OE_9_vs_In_OE_9_peak_502	Solyc02g088180.3
4.80367	IP_OE_9_vs_In_OE_9_peak_503	Solyc02g088510.3
6.18486	IP_OE_9_vs_In_OE_9_peak_504	Solyc02g088570.2
7.42183	IP_OE_9_vs_In_OE_9_peak_505	Solyc02g088730.2
3.99252	IP_OE_9_vs_In_OE_9_peak_506	Solyc02g089090.3
4.03575	IP_OE_9_vs_In_OE_9_peak_507	Solyc02g089100.3
6.21059	IP_OE_9_vs_In_OE_9_peak_508	Solyc02g089160.3
5.1057	IP_OE_9_vs_In_OE_9_peak_509	Solyc02g089190.2
5.13397	IP_OE_9_vs_In_OE_9_peak_510	Solyc02g089550.3
4.43613	IP_OE_9_vs_In_OE_9_peak_511	Solyc02g089640.3
5.32336	IP_OE_9_vs_In_OE_9_peak_512	Solyc02g089760.3
4.43613	IP_OE_9_vs_In_OE_9_peak_513	Solyc02g089840.3
4.43613	IP_OE_9_vs_In_OE_9_peak_514	Solyc02g089850.3
6.21059	IP_OE_9_vs_In_OE_9_peak_515	Solyc02g089870.2

3.71092	IP_OE_9_vs_In_OE_9_peak_516	Solyc02g089900.1
6.21059	IP_OE_9_vs_In_OE_9_peak_517	Solyc02g089980.3
3.02835	IP_OE_9_vs_In_OE_9_peak_518	Solyc02g089990.1
5.1364	IP_OE_9_vs_In_OE_9_peak_519	Solyc07g032640.2
4.56467	IP_OE_9_vs_In_OE_9_peak_520	Solyc02g090160.3
4.87975	IP_OE_9_vs_In_OE_9_peak_521	Solyc02g090350.3
7.83415	IP_OE_9_vs_In_OE_9_peak_522	Solyc02g090410.3
4.83097	IP_OE_9_vs_In_OE_9_peak_523	Solyc02g090580.3
7.09781	IP_OE_9_vs_In_OE_9_peak_524	Solyc02g090870.1
4.77667	IP_OE_9_vs_In_OE_9_peak_525	Solyc02g090890.3
3.98416	IP_OE_9_vs_In_OE_9_peak_526	Solyc02g091430.3
4.6952	IP_OE_9_vs_In_OE_9_peak_527	Solyc02g091560.3
4.53556	IP_OE_9_vs_In_OE_9_peak_528	Solyc02g091660.3
5.80833	IP_OE_9_vs_In_OE_9_peak_529	Solyc02g091840.3
6.60395	IP_OE_9_vs_In_OE_9_peak_530	Solyc02g091860.3
3.62306	IP_OE_9_vs_In_OE_9_peak_531	Solyc02g091970.3
4.12324	IP_OE_9_vs_In_OE_9_peak_532	Solyc02g091990.3
5.32336	IP_OE_9_vs_In_OE_9_peak_533	Solyc02g092210.1
3.99252	IP_OE_9_vs_In_OE_9_peak_534	Solyc02g092330.3
6.21059	IP_OE_9_vs_In_OE_9_peak_535	Solyc02g092440.3
4.87975	IP_OE_9_vs_In_OE_9_peak_536	Solyc02g092470.3
4.87975	IP_OE_9_vs_In_OE_9_peak_537	Solyc02g092527.1
3.95242	IP_OE_9_vs_In_OE_9_peak_538	Solyc02g092537.1
4.12324	IP_OE_9_vs_In_OE_9_peak_539	Solyc02g092550.3
4.74997	IP_OE_9_vs_In_OE_9_peak_540	Solyc02g092580.3
4.23654	IP_OE_9_vs_In_OE_9_peak_541	Solyc02g092700.3
3.8835	IP_OE_9_vs_In_OE_9_peak_542	Solyc02g092750.3
3.99252	IP_OE_9_vs_In_OE_9_peak_543	Solyc02g092790.3
4.87717	IP_OE_9_vs_In_OE_9_peak_544	Solyc02g092800.3
4.87975	IP_OE_9_vs_In_OE_9_peak_545	Solyc02g092820.3
4.43379	IP_OE_9_vs_In_OE_9_peak_546	Solyc02g092840.1
4.87975	IP_OE_9_vs_In_OE_9_peak_547	Solyc02g093020.3
5.32336	IP_OE_9_vs_In_OE_9_peak_548	Solyc02g093050.3
4.43613	IP_OE_9_vs_In_OE_9_peak_549	Solyc02g093060.3
5.29568	IP_OE_9_vs_In_OE_9_peak_550	Solyc02g093110.3
3.99252	IP_OE_9_vs_In_OE_9_peak_551	Solyc02g093130.2
4.87975	IP_OE_9_vs_In_OE_9_peak_552	Solyc02g093140.3
4.56467	IP_OE_9_vs_In_OE_9_peak_553	Solyc02g093150.3
5.32336	IP_OE_9_vs_In_OE_9_peak_554	Solyc02g093180.3
5.32336	IP_OE_9_vs_In_OE_9_peak_555	Solyc02g093270.3
4.68022	IP_OE_9_vs_In_OE_9_peak_556	Solyc02g093290.3
4.87975	IP_OE_9_vs_In_OE_9_peak_557	Solyc02g093410.3
3.62306	IP_OE_9_vs_In_OE_9_peak_558	Solyc02g093590.3
5.76697	IP_OE_9_vs_In_OE_9_peak_559	Solyc02g093680.3
3.62306	IP_OE_9_vs_In_OE_9_peak_560	Solyc02g093700.3
5.77763	IP_OE_9_vs_In_OE_9_peak_561	Solyc02g093860.3
3.71092	IP_OE_9_vs_In_OE_9_peak_562	Solyc02g094030.3

3.71092	IP_OE_9_vs_In_OE_9_peak_563	Solyc02g094300.3
4.53556	IP_OE_9_vs_In_OE_9_peak_564	Solyc03g005770.3
4.45922	IP_OE_9_vs_In_OE_9_peak_565	Solyc03g006840.3
3.99252	IP_OE_9_vs_In_OE_9_peak_566	Solyc03g007050.3
2.41283	IP_OE_9_vs_In_OE_9_peak_567	Solyc03g007400.2
4.53556	IP_OE_9_vs_In_OE_9_peak_568	Solyc03g007430.3
4.87975	IP_OE_9_vs_In_OE_9_peak_569	Solyc03g007680.3
4.87975	IP_OE_9_vs_In_OE_9_peak_570	Solyc03g007740.3
4.61116	IP_OE_9_vs_In_OE_9_peak_571	Solyc03g007775.1
4.43613	IP_OE_9_vs_In_OE_9_peak_572	Solyc03g007780.1
4.94789	IP_OE_9_vs_In_OE_9_peak_573	Solyc03g007790.3
4.87975	IP_OE_9_vs_In_OE_9_peak_574	Solyc03g025160.3
4.76774	IP_OE_9_vs_In_OE_9_peak_575	Solyc03g025420.3
5.70583	IP_OE_9_vs_In_OE_9_peak_576	Solyc03g025730.3
3.71092	IP_OE_9_vs_In_OE_9_peak_577	Solyc03g025795.1
3.4417	IP_OE_9_vs_In_OE_9_peak_578	Solyc03g026210.3
4.25475	IP_OE_9_vs_In_OE_9_peak_579	Solyc03g026360.1
4.94263	IP_OE_9_vs_In_OE_9_peak_580	Solyc03g031420.1
3.99252	IP_OE_9_vs_In_OE_9_peak_581	Solyc03g031850.1
4.18428	IP_OE_9_vs_In_OE_9_peak_582	Solyc03g031970.3
6.70786	IP_OE_9_vs_In_OE_9_peak_583	Solyc03g032000.3
4.54656	IP_OE_9_vs_In_OE_9_peak_584	Solyc03g033590.1
4.40263	IP_OE_9_vs_In_OE_9_peak_585	Solyc03g033840.3
4.43613	IP_OE_9_vs_In_OE_9_peak_586	Solyc03g034060.3
4.94263	IP_OE_9_vs_In_OE_9_peak_587	Solyc03g034370.1
5.00728	IP_OE_9_vs_In_OE_9_peak_588	Solyc03g043640.3
4.87975	IP_OE_9_vs_In_OE_9_peak_589	Solyc03g043900.1
6.00177	IP_OE_9_vs_In_OE_9_peak_590	Solyc03g044097.1
6.08622	IP_OE_9_vs_In_OE_9_peak_591	Solyc03g044300.3
5.32336	IP_OE_9_vs_In_OE_9_peak_592	Solyc03g044580.1
3.92288	IP_OE_9_vs_In_OE_9_peak_593	Solyc03g046470.3
5.32544	IP_OE_9_vs_In_OE_9_peak_594	Solyc03g071700.1
3.54891	IP_OE_9_vs_In_OE_9_peak_595	Solyc03g053027.1
8.71751	IP_OE_9_vs_In_OE_9_peak_596	Solyc03g053080.2
3.95261	IP_OE_9_vs_In_OE_9_peak_597	Solyc03g058150.1
4.12324	IP_OE_9_vs_In_OE_9_peak_598	Solyc03g058160.3
6.96092	IP_OE_9_vs_In_OE_9_peak_599	Solyc03g058210.2
4.30852	IP_OE_9_vs_In_OE_9_peak_600	Solyc03g058630.1
4.59918	IP_OE_9_vs_In_OE_9_peak_601	Solyc03g061540.1
6.46661	IP_OE_9_vs_In_OE_9_peak_602	Solyc03g061550.1
5.09189	IP_OE_9_vs_In_OE_9_peak_603	Solyc03g061655.1
5.76697	IP_OE_9_vs_In_OE_9_peak_604	Solyc03g062660.3
4.23654	IP_OE_9_vs_In_OE_9_peak_605	Solyc03g062780.1
3.9828	IP_OE_9_vs_In_OE_9_peak_606	Solyc03g063070.1
5.32336	IP_OE_9_vs_In_OE_9_peak_607	Solyc03g063515.1
9.12933	IP_OE_9_vs_In_OE_9_peak_608	Solyc03g063585.1
4.43613	IP_OE_9_vs_In_OE_9_peak_609	Solyc03g063600.4

5.66638	IP_OE_9_vs_In_OE_9_peak_610	Solyc03g063860.1
4.33431	IP_OE_9_vs_In_OE_9_peak_611	Solyc03g013610.1
6.6542	IP_OE_9_vs_In_OE_9_peak_612	Solyc03g013600.1
5.32336	IP_OE_9_vs_In_OE_9_peak_613	Solyc03g078480.2
4.87975	IP_OE_9_vs_In_OE_9_peak_614	Solyc03g078630.3
5.76697	IP_OE_9_vs_In_OE_9_peak_615	Solyc03g078640.1
3.54891	IP_OE_9_vs_In_OE_9_peak_616	Solyc03g078650.3
3.95242	IP_OE_9_vs_In_OE_9_peak_617	Solyc03g079980.2
5.64872	IP_OE_9_vs_In_OE_9_peak_618	Solyc03g080090.3
4.87975	IP_OE_9_vs_In_OE_9_peak_619	Solyc03g080110.3
4.43613	IP_OE_9_vs_In_OE_9_peak_620	Solyc03g080120.1
3.48701	IP_OE_9_vs_In_OE_9_peak_621	Solyc03g081335.1
4.87975	IP_OE_9_vs_In_OE_9_peak_622	Solyc03g082540.3
5.32336	IP_OE_9_vs_In_OE_9_peak_623	Solyc03g082610.1
5.32336	IP_OE_9_vs_In_OE_9_peak_624	Solyc03g082720.3
5.57783	IP_OE_9_vs_In_OE_9_peak_625	Solyc03g083260.1
5.4217	IP_OE_9_vs_In_OE_9_peak_626	Solyc03g083340.2
6.21059	IP_OE_9_vs_In_OE_9_peak_627	Solyc03g083360.3
5.76697	IP_OE_9_vs_In_OE_9_peak_628	Solyc03g083430.3
4.25988	IP_OE_9_vs_In_OE_9_peak_629	Solyc03g083440.3
5.70583	IP_OE_9_vs_In_OE_9_peak_630	Solyc03g083460.3
4.56467	IP_OE_9_vs_In_OE_9_peak_631	Solyc03g083615.1
5.64515	IP_OE_9_vs_In_OE_9_peak_632	Solyc03g083910.4
6.08622	IP_OE_9_vs_In_OE_9_peak_633	Solyc03g083970.3
4.87975	IP_OE_9_vs_In_OE_9_peak_634	Solyc03g093140.3
4.26754	IP_OE_9_vs_In_OE_9_peak_635	Solyc03g093310.3
4.94053	IP_OE_9_vs_In_OE_9_peak_636	Solyc03g095940.1
6.18486	IP_OE_9_vs_In_OE_9_peak_637	Solyc03g096050.3
4.9424	IP_OE_9_vs_In_OE_9_peak_638	Solyc03g096390.3
4.12324	IP_OE_9_vs_In_OE_9_peak_639	Solyc03g097030.3
4.43613	IP_OE_9_vs_In_OE_9_peak_640	Solyc03g097120.3
7.54143	IP_OE_9_vs_In_OE_9_peak_641	Solyc03g097670.3
5.77763	IP_OE_9_vs_In_OE_9_peak_642	Solyc03g097690.3
4.18428	IP_OE_9_vs_In_OE_9_peak_643	Solyc03g097730.2
4.56467	IP_OE_9_vs_In_OE_9_peak_644	Solyc03g097740.1
4.31816	IP_OE_9_vs_In_OE_9_peak_645	Solyc03g097886.1
4.58959	IP_OE_9_vs_In_OE_9_peak_646	Solyc03g097920.1
4.54656	IP_OE_9_vs_In_OE_9_peak_647	Solyc03g097940.3
4.56467	IP_OE_9_vs_In_OE_9_peak_648	Solyc03g098080.1
3.90818	IP_OE_9_vs_In_OE_9_peak_649	Solyc03g098630.3
4.43613	IP_OE_9_vs_In_OE_9_peak_650	Solyc03g098730.1
6.6542	IP_OE_9_vs_In_OE_9_peak_651	Solyc03g111170.3
3.485	IP_OE_9_vs_In_OE_9_peak_652	Solyc03g111690.3
4.39179	IP_OE_9_vs_In_OE_9_peak_653	Solyc03g111710.3
4.38258	IP_OE_9_vs_In_OE_9_peak_654	Solyc03g111995.1
4.56467	IP_OE_9_vs_In_OE_9_peak_655	Solyc03g112060.3
3.39535	IP_OE_9_vs_In_OE_9_peak_656	Solyc03g112150.1

6.18486	IP_OE_9_vs_In_OE_9_peak_657	Solyc03g112297.1
4.28179	IP_OE_9_vs_In_OE_9_peak_658	Solyc03g112300.3
4.83097	IP_OE_9_vs_In_OE_9_peak_659	Solyc03g112310.1
4.79463	IP_OE_9_vs_In_OE_9_peak_660	Solyc03g112335.1
4.87975	IP_OE_9_vs_In_OE_9_peak_661	Solyc03g112340.1
4.36697	IP_OE_9_vs_In_OE_9_peak_662	Solyc03g112460.3
4.56467	IP_OE_9_vs_In_OE_9_peak_663	Solyc03g112540.3
4.43613	IP_OE_9_vs_In_OE_9_peak_664	Solyc03g112710.1
7.09781	IP_OE_9_vs_In_OE_9_peak_665	Solyc03g112930.3
5.27015	IP_OE_9_vs_In_OE_9_peak_666	Solyc03g113020.3
6.46661	IP_OE_9_vs_In_OE_9_peak_667	Solyc03g113040.3
4.69747	IP_OE_9_vs_In_OE_9_peak_668	Solyc03g113120.3
4.87975	IP_OE_9_vs_In_OE_9_peak_669	Solyc03g113270.3
4.94789	IP_OE_9_vs_In_OE_9_peak_670	Solyc03g113420.3
4.5386	IP_OE_9_vs_In_OE_9_peak_671	Solyc03g113450.3
3.99252	IP_OE_9_vs_In_OE_9_peak_672	Solyc03g114020.3
4.37506	IP_OE_9_vs_In_OE_9_peak_673	Solyc03g114030.3
3.54891	IP_OE_9_vs_In_OE_9_peak_674	Solyc03g114160.1
4.30852	IP_OE_9_vs_In_OE_9_peak_675	Solyc03g114330.3
4.87975	IP_OE_9_vs_In_OE_9_peak_676	Solyc03g114400.3
4.43613	IP_OE_9_vs_In_OE_9_peak_677	Solyc03g114593.1
4.43613	IP_OE_9_vs_In_OE_9_peak_678	Solyc03g114690.3
3.99252	IP_OE_9_vs_In_OE_9_peak_679	Solyc03g114710.3
4.43613	IP_OE_9_vs_In_OE_9_peak_680	Solyc03g114720.3
5.24036	IP_OE_9_vs_In_OE_9_peak_681	Solyc03g114730.3
4.43613	IP_OE_9_vs_In_OE_9_peak_682	Solyc03g114840.3
3.8835	IP_OE_9_vs_In_OE_9_peak_683	Solyc03g115020.3
5.76697	IP_OE_9_vs_In_OE_9_peak_684	Solyc03g115040.3
6.11283	IP_OE_9_vs_In_OE_9_peak_685	Solyc03g115220.3
4.43613	IP_OE_9_vs_In_OE_9_peak_686	Solyc03g115247.1
5.30432	IP_OE_9_vs_In_OE_9_peak_687	Solyc03g115460.2
2.83027	IP_OE_9_vs_In_OE_9_peak_688	Solyc03g115680.3
3.54891	IP_OE_9_vs_In_OE_9_peak_689	Solyc03g115720.3
3.77217	IP_OE_9_vs_In_OE_9_peak_690	Solyc03g115770.3
2.43715	IP_OE_9_vs_In_OE_9_peak_691	Solyc03g115920.3
2.2531	IP_OE_9_vs_In_OE_9_peak_692	Solyc03g115930.2
1.88048	IP_OE_9_vs_In_OE_9_peak_693	Solyc03g115990.2
4.43613	IP_OE_9_vs_In_OE_9_peak_694	Solyc03g116100.3
3.28323	IP_OE_9_vs_In_OE_9_peak_695	Solyc03g116110.3
2.96088	IP_OE_9_vs_In_OE_9_peak_696	Solyc03g116200.2
2.27889	IP_OE_9_vs_In_OE_9_peak_697	Solyc03g116320.3
1.87267	IP_OE_9_vs_In_OE_9_peak_698	Solyc03g116335.1
4.25103	IP_OE_9_vs_In_OE_9_peak_699	Solyc03g116350.3
5.04093	IP_OE_9_vs_In_OE_9_peak_700	Solyc03g116480.1
3.8835	IP_OE_9_vs_In_OE_9_peak_701	Solyc03g116490.1
3.99252	IP_OE_9_vs_In_OE_9_peak_702	Solyc03g116590.3
3.07661	IP_OE_9_vs_In_OE_9_peak_703	Solyc03g116670.3

3.68475	IP_OE_9_vs_In_OE_9_peak_704	Solyc03g116730.3
4.62211	IP_OE_9_vs_In_OE_9_peak_705	Solyc03g116770.3
5.64872	IP_OE_9_vs_In_OE_9_peak_706	Solyc03g117210.3
4.87975	IP_OE_9_vs_In_OE_9_peak_707	Solyc03g117300.3
4.43613	IP_OE_9_vs_In_OE_9_peak_708	Solyc03g117350.1
4.58959	IP_OE_9_vs_In_OE_9_peak_709	Solyc03g117360.3
4.45922	IP_OE_9_vs_In_OE_9_peak_710	Solyc03g117420.3
4.87717	IP_OE_9_vs_In_OE_9_peak_711	Solyc03g117490.3
4.12324	IP_OE_9_vs_In_OE_9_peak_712	Solyc03g117540.3
6.06207	IP_OE_9_vs_In_OE_9_peak_713	Solyc03g117590.3
6.847	IP_OE_9_vs_In_OE_9_peak_714	Solyc03g117600.3
4.87975	IP_OE_9_vs_In_OE_9_peak_715	Solyc03g117610.1
4.43613	IP_OE_9_vs_In_OE_9_peak_716	Solyc03g117675.1
3.62306	IP_OE_9_vs_In_OE_9_peak_717	Solyc03g117750.3
4.87975	IP_OE_9_vs_In_OE_9_peak_718	Solyc03g117860.3
5.55603	IP_OE_9_vs_In_OE_9_peak_719	Solyc03g117870.3
4.43613	IP_OE_9_vs_In_OE_9_peak_720	Solyc03g117980.3
5.87526	IP_OE_9_vs_In_OE_9_peak_721	Solyc03g118040.3
7.72949	IP_OE_9_vs_In_OE_9_peak_722	Solyc03g118190.3
5.32336	IP_OE_9_vs_In_OE_9_peak_723	Solyc03g118225.1
6.10226	IP_OE_9_vs_In_OE_9_peak_724	Solyc03g118290.3
5.66638	IP_OE_9_vs_In_OE_9_peak_725	Solyc03g118620.3
6.9036	IP_OE_9_vs_In_OE_9_peak_726	Solyc03g118760.3
4.43613	IP_OE_9_vs_In_OE_9_peak_727	Solyc03g118770.3
4.28179	IP_OE_9_vs_In_OE_9_peak_728	Solyc03g118820.3
3.97521	IP_OE_9_vs_In_OE_9_peak_729	Solyc03g118840.3
5.66638	IP_OE_9_vs_In_OE_9_peak_730	Solyc03g119080.4
4.30852	IP_OE_9_vs_In_OE_9_peak_731	Solyc03g119250.3
3.485	IP_OE_9_vs_In_OE_9_peak_732	Solyc03g119260.3
3.80389	IP_OE_9_vs_In_OE_9_peak_733	Solyc03g119440.3
3.2881	IP_OE_9_vs_In_OE_9_peak_734	Solyc03g119520.3
4.94506	IP_OE_9_vs_In_OE_9_peak_735	Solyc03g119540.3
4.87975	IP_OE_9_vs_In_OE_9_peak_736	Solyc03g119560.1
5.70583	IP_OE_9_vs_In_OE_9_peak_737	Solyc03g119610.1
2.78446	IP_OE_9_vs_In_OE_9_peak_738	Solyc03g119910.3
4.43613	IP_OE_9_vs_In_OE_9_peak_739	Solyc03g119930.1
3.99252	IP_OE_9_vs_In_OE_9_peak_740	Solyc03g120170.1
3.85048	IP_OE_9_vs_In_OE_9_peak_741	Solyc03g120390.3
5.04093	IP_OE_9_vs_In_OE_9_peak_742	Solyc03g120550.2
4.94789	IP_OE_9_vs_In_OE_9_peak_743	Solyc03g120770.3
5.76697	IP_OE_9_vs_In_OE_9_peak_744	Solyc03g120810.3
4.87975	IP_OE_9_vs_In_OE_9_peak_745	Solyc03g120890.3
3.99252	IP_OE_9_vs_In_OE_9_peak_746	Solyc03g120910.3
5.36021	IP_OE_9_vs_In_OE_9_peak_747	Solyc03g120970.3
3.54891	IP_OE_9_vs_In_OE_9_peak_748	Solyc03g121140.3
5.76697	IP_OE_9_vs_In_OE_9_peak_749	Solyc03g121190.3
4.81409	IP_OE_9_vs_In_OE_9_peak_750	Solyc03g121260.3

4.53556	IP_OE_9_vs_In_OE_9_peak_751	Solyc03g121400.1
3.70402	IP_OE_9_vs_In_OE_9_peak_752	Solyc03g121660.3
5.32544	IP_OE_9_vs_In_OE_9_peak_753	Solyc03g121670.3
7.442	IP_OE_9_vs_In_OE_9_peak_754	Solyc03g121760.3
4.28179	IP_OE_9_vs_In_OE_9_peak_755	Solyc03g121860.1
4.43613	IP_OE_9_vs_In_OE_9_peak_756	Solyc03g121880.3
3.39535	IP_OE_9_vs_In_OE_9_peak_757	Solyc03g121960.3
3.92288	IP_OE_9_vs_In_OE_9_peak_758	Solyc03g121980.3
3.99252	IP_OE_9_vs_In_OE_9_peak_759	Solyc03g122000.3
4.42533	IP_OE_9_vs_In_OE_9_peak_760	Solyc03g122300.2
5.36021	IP_OE_9_vs_In_OE_9_peak_761	Solyc03g123370.3
7.52837	IP_OE_9_vs_In_OE_9_peak_762	Solyc03g123420.1
4.58959	IP_OE_9_vs_In_OE_9_peak_763	Solyc03g123490.1
6.189	IP_OE_9_vs_In_OE_9_peak_764	Solyc03g123630.3
5.58241	IP_OE_9_vs_In_OE_9_peak_765	Solyc03g123860.3
4.31816	IP_OE_9_vs_In_OE_9_peak_766	Solyc04g005050.1
4.43613	IP_OE_9_vs_In_OE_9_peak_767	Solyc04g005060.3
8.42865	IP_OE_9_vs_In_OE_9_peak_768	Solyc04g005290.3
5.32336	IP_OE_9_vs_In_OE_9_peak_769	Solyc04g005650.2
4.43613	IP_OE_9_vs_In_OE_9_peak_770	Solyc04g005660.3
4.9512	IP_OE_9_vs_In_OE_9_peak_771	Solyc04g005800.3
4.62085	IP_OE_9_vs_In_OE_9_peak_772	Solyc04g005830.3
4.67165	IP_OE_9_vs_In_OE_9_peak_773	Solyc04g006990.3
4.63003	IP_OE_9_vs_In_OE_9_peak_774	Solyc04g007200.3
4.43613	IP_OE_9_vs_In_OE_9_peak_775	Solyc04g007270.3
5.76697	IP_OE_9_vs_In_OE_9_peak_776	Solyc04g007660.1
6.46661	IP_OE_9_vs_In_OE_9_peak_777	Solyc04g007700.3
4.5386	IP_OE_9_vs_In_OE_9_peak_778	Solyc04g007870.3
3.71092	IP_OE_9_vs_In_OE_9_peak_779	Solyc04g008070.2
5.09189	IP_OE_9_vs_In_OE_9_peak_780	Solyc04g008210.2
4.43613	IP_OE_9_vs_In_OE_9_peak_781	Solyc04g008480.2
3.62306	IP_OE_9_vs_In_OE_9_peak_782	Solyc04g009040.3
4.75884	IP_OE_9_vs_In_OE_9_peak_783	Solyc04g009870.1
4.53556	IP_OE_9_vs_In_OE_9_peak_784	Solyc04g009900.3
3.71092	IP_OE_9_vs_In_OE_9_peak_785	Solyc04g009980.3
6.00071	IP_OE_9_vs_In_OE_9_peak_786	Solyc04g012120.3
4.40263	IP_OE_9_vs_In_OE_9_peak_787	Solyc04g014250.3
4.94506	IP_OE_9_vs_In_OE_9_peak_788	Solyc04g014400.3
5.66638	IP_OE_9_vs_In_OE_9_peak_789	Solyc04g014530.1
5.36021	IP_OE_9_vs_In_OE_9_peak_790	Solyc04g015020.3
5.36021	IP_OE_9_vs_In_OE_9_peak_791	Solyc04g015030.3
6.21059	IP_OE_9_vs_In_OE_9_peak_792	Solyc04g015360.3
6.41746	IP_OE_9_vs_In_OE_9_peak_793	Solyc04g015600.3
4.18428	IP_OE_9_vs_In_OE_9_peak_794	Solyc04g015765.1
4.43613	IP_OE_9_vs_In_OE_9_peak_795	Solyc04g015850.2
5.76697	IP_OE_9_vs_In_OE_9_peak_796	Solyc04g015970.3
3.99252	IP_OE_9_vs_In_OE_9_peak_797	Solyc04g016250.3

4.4169	IP_OE_9_vs_In_OE_9_peak_798	Solyc04g016430.3
4.87717	IP_OE_9_vs_In_OE_9_peak_799	Solyc04g016460.3
4.87717	IP_OE_9_vs_In_OE_9_peak_800	Solyc04g017685.1
4.75972	IP_OE_9_vs_In_OE_9_peak_801	Solyc04g017690.3
4.18428	IP_OE_9_vs_In_OE_9_peak_802	Solyc04g017750.3
5.2699	IP_OE_9_vs_In_OE_9_peak_803	Solyc04g019330.1
4.23654	IP_OE_9_vs_In_OE_9_peak_804	Solyc04g039850.1
3.99041	IP_OE_9_vs_In_OE_9_peak_805	Solyc04g039840.1
3.66886	IP_OE_9_vs_In_OE_9_peak_806	Solyc04g039830.2
6.87065	IP_OE_9_vs_In_OE_9_peak_807	Solyc04g039810.1
4.71484	IP_OE_9_vs_In_OE_9_peak_808	Solyc04g039800.2
4.87975	IP_OE_9_vs_In_OE_9_peak_809	Solyc04g028580.2
4.93869	IP_OE_9_vs_In_OE_9_peak_810	Solyc04g026110.3
5.47092	IP_OE_9_vs_In_OE_9_peak_811	Solyc04g025290.3
3.99252	IP_OE_9_vs_In_OE_9_peak_812	Solyc04g024840.3
3.71092	IP_OE_9_vs_In_OE_9_peak_813	Solyc04g024710.3
4.94789	IP_OE_9_vs_In_OE_9_peak_814	Solyc04g024510.3
5.67538	IP_OE_9_vs_In_OE_9_peak_815	Solyc04g045300.1
6.59718	IP_OE_9_vs_In_OE_9_peak_816	Solyc04g049090.3
4.12324	IP_OE_9_vs_In_OE_9_peak_817	Solyc04g049150.2
4.94506	IP_OE_9_vs_In_OE_9_peak_818	Solyc04g049350.3
7.00951	IP_OE_9_vs_In_OE_9_peak_819	Solyc04g049920.3
5.32336	IP_OE_9_vs_In_OE_9_peak_820	Solyc04g050440.3
3.99041	IP_OE_9_vs_In_OE_9_peak_821	Solyc04g050490.3
6.60583	IP_OE_9_vs_In_OE_9_peak_822	Solyc04g050720.3
4.43613	IP_OE_9_vs_In_OE_9_peak_823	Solyc04g050790.3
4.26754	IP_OE_9_vs_In_OE_9_peak_824	Solyc04g050840.1
3.99252	IP_OE_9_vs_In_OE_9_peak_825	Solyc04g051513.1
5.73104	IP_OE_9_vs_In_OE_9_peak_826	Solyc04g051800.3
5.32336	IP_OE_9_vs_In_OE_9_peak_827	Solyc04g054910.3
5.23051	IP_OE_9_vs_In_OE_9_peak_828	Solyc04g054930.3
4.30213	IP_OE_9_vs_In_OE_9_peak_829	Solyc04g055030.2
4.55481	IP_OE_9_vs_In_OE_9_peak_830	Solyc04g055090.1
5.76697	IP_OE_9_vs_In_OE_9_peak_831	Solyc04g056310.3
4.5386	IP_OE_9_vs_In_OE_9_peak_832	Solyc04g071030.1
3.66126	IP_OE_9_vs_In_OE_9_peak_833	Solyc04g071040.3
4.94263	IP_OE_9_vs_In_OE_9_peak_834	Solyc04g071080.1
3.71092	IP_OE_9_vs_In_OE_9_peak_835	Solyc04g071360.3
5.36021	IP_OE_9_vs_In_OE_9_peak_836	Solyc04g071480.1
5.36021	IP_OE_9_vs_In_OE_9_peak_837	Solyc04g071615.1
4.10065	IP_OE_9_vs_In_OE_9_peak_838	Solyc04g071640.1
4.43613	IP_OE_9_vs_In_OE_9_peak_839	Solyc04g071770.3
5.32336	IP_OE_9_vs_In_OE_9_peak_840	Solyc04g071890.3
4.87975	IP_OE_9_vs_In_OE_9_peak_841	Solyc04g071940.3
7.09781	IP_OE_9_vs_In_OE_9_peak_842	Solyc04g071990.3
5.08702	IP_OE_9_vs_In_OE_9_peak_843	Solyc04g072020.3
4.26257	IP_OE_9_vs_In_OE_9_peak_844	Solyc04g072100.1

3.99252	IP_OE_9_vs_In_OE_9_peak_845	Solyc04g072480.3
5.21091	IP_OE_9_vs_In_OE_9_peak_846	Solyc04g072785.1
3.71092	IP_OE_9_vs_In_OE_9_peak_847	Solyc04g072850.3
5.17941	IP_OE_9_vs_In_OE_9_peak_848	Solyc04g072900.1
4.87975	IP_OE_9_vs_In_OE_9_peak_849	Solyc04g073960.3
4.43613	IP_OE_9_vs_In_OE_9_peak_850	Solyc04g074230.3
3.80389	IP_OE_9_vs_In_OE_9_peak_851	Solyc04g074290.3
5.88108	IP_OE_9_vs_In_OE_9_peak_852	Solyc04g074300.3
4.28622	IP_OE_9_vs_In_OE_9_peak_853	Solyc04g074410.2
5.36021	IP_OE_9_vs_In_OE_9_peak_854	Solyc04g074510.3
4.40263	IP_OE_9_vs_In_OE_9_peak_855	Solyc04g074680.1
3.58575	IP_OE_9_vs_In_OE_9_peak_856	Solyc04g074700.3
4.5386	IP_OE_9_vs_In_OE_9_peak_857	Solyc04g074800.3
4.61116	IP_OE_9_vs_In_OE_9_peak_858	Solyc04g074980.3
4.56467	IP_OE_9_vs_In_OE_9_peak_859	Solyc04g076020.3
4.43613	IP_OE_9_vs_In_OE_9_peak_860	Solyc04g076050.3
5.32544	IP_OE_9_vs_In_OE_9_peak_861	Solyc04g076190.1
5.27923	IP_OE_9_vs_In_OE_9_peak_862	Solyc04g076210.2
6.56282	IP_OE_9_vs_In_OE_9_peak_863	Solyc04g076390.3
4.49064	IP_OE_9_vs_In_OE_9_peak_864	Solyc04g076610.2
2.87136	IP_OE_9_vs_In_OE_9_peak_865	Solyc04g076715.1
3.99252	IP_OE_9_vs_In_OE_9_peak_866	Solyc04g076780.3
4.20305	IP_OE_9_vs_In_OE_9_peak_867	Solyc04g076940.3
7.98504	IP_OE_9_vs_In_OE_9_peak_868	Solyc04g076980.3
4.38349	IP_OE_9_vs_In_OE_9_peak_869	Solyc04g077020.3
4.87975	IP_OE_9_vs_In_OE_9_peak_870	Solyc04g077260.3
4.56467	IP_OE_9_vs_In_OE_9_peak_871	Solyc04g077360.3
4.45922	IP_OE_9_vs_In_OE_9_peak_872	Solyc04g077450.3
3.99252	IP_OE_9_vs_In_OE_9_peak_873	Solyc04g077500.3
5.32336	IP_OE_9_vs_In_OE_9_peak_874	Solyc04g077510.3
4.56467	IP_OE_9_vs_In_OE_9_peak_875	Solyc04g077530.1
4.70021	IP_OE_9_vs_In_OE_9_peak_876	Solyc04g077615.1
4.31816	IP_OE_9_vs_In_OE_9_peak_877	Solyc04g077700.1
3.96765	IP_OE_9_vs_In_OE_9_peak_878	Solyc04g077860.3
5.32336	IP_OE_9_vs_In_OE_9_peak_879	Solyc04g077950.2
3.80445	IP_OE_9_vs_In_OE_9_peak_880	Solyc04g077970.3
2.54518	IP_OE_9_vs_In_OE_9_peak_881	Solyc04g078310.3
4.43613	IP_OE_9_vs_In_OE_9_peak_882	Solyc04g078390.2
6.6542	IP_OE_9_vs_In_OE_9_peak_883	Solyc04g078420.1
5.93486	IP_OE_9_vs_In_OE_9_peak_884	Solyc04g078440.3
4.29379	IP_OE_9_vs_In_OE_9_peak_885	Solyc04g078430.2
6.21059	IP_OE_9_vs_In_OE_9_peak_886	Solyc04g078480.3
3.99252	IP_OE_9_vs_In_OE_9_peak_887	Solyc04g078490.1
5.32336	IP_OE_9_vs_In_OE_9_peak_888	Solyc04g078550.3
3.80389	IP_OE_9_vs_In_OE_9_peak_889	Solyc04g078560.1
5.76697	IP_OE_9_vs_In_OE_9_peak_890	Solyc04g078830.3
3.70402	IP_OE_9_vs_In_OE_9_peak_891	Solyc04g079110.1

4.87975	IP_OE_9_vs_In_OE_9_peak_892	Solyc04g079250.3
3.54891	IP_OE_9_vs_In_OE_9_peak_893	Solyc04g079330.2
4.18428	IP_OE_9_vs_In_OE_9_peak_894	Solyc04g079350.1
6.6542	IP_OE_9_vs_In_OE_9_peak_895	Solyc04g079420.3
6.21059	IP_OE_9_vs_In_OE_9_peak_896	Solyc04g079550.3
4.43613	IP_OE_9_vs_In_OE_9_peak_897	Solyc04g079643.1
4.06583	IP_OE_9_vs_In_OE_9_peak_898	Solyc04g079700.3
3.99252	IP_OE_9_vs_In_OE_9_peak_899	Solyc04g079870.2
5.36021	IP_OE_9_vs_In_OE_9_peak_900	Solyc04g079900.3
3.53045	IP_OE_9_vs_In_OE_9_peak_901	Solyc04g079930.3
4.18428	IP_OE_9_vs_In_OE_9_peak_902	Solyc04g080040.3
4.43613	IP_OE_9_vs_In_OE_9_peak_903	Solyc04g080130.3
5.32544	IP_OE_9_vs_In_OE_9_peak_904	Solyc04g080290.3
4.63003	IP_OE_9_vs_In_OE_9_peak_905	Solyc04g080440.3
6.32408	IP_OE_9_vs_In_OE_9_peak_906	Solyc04g080450.1
3.99252	IP_OE_9_vs_In_OE_9_peak_907	Solyc04g080540.2
4.56467	IP_OE_9_vs_In_OE_9_peak_908	Solyc04g080610.3
5.98653	IP_OE_9_vs_In_OE_9_peak_909	Solyc04g080720.3
4.12324	IP_OE_9_vs_In_OE_9_peak_910	Solyc04g080740.1
4.31816	IP_OE_9_vs_In_OE_9_peak_911	Solyc04g080960.3
3.7807	IP_OE_9_vs_In_OE_9_peak_912	Solyc04g081180.1
5.7764	IP_OE_9_vs_In_OE_9_peak_913	Solyc04g081235.1
3.11191	IP_OE_9_vs_In_OE_9_peak_914	Solyc04g081250.1
2.99372	IP_OE_9_vs_In_OE_9_peak_915	Solyc04g081290.3
2.27271	IP_OE_9_vs_In_OE_9_peak_916	Solyc04g081300.3
2.25347	IP_OE_9_vs_In_OE_9_peak_917	Solyc04g081430.3
2.56148	IP_OE_9_vs_In_OE_9_peak_918	Solyc04g081550.3
2.24766	IP_OE_9_vs_In_OE_9_peak_919	Solyc04g081560.3
4.27486	IP_OE_9_vs_In_OE_9_peak_920	Solyc04g081570.3
6.6542	IP_OE_9_vs_In_OE_9_peak_921	Solyc04g081695.1
4.92544	IP_OE_9_vs_In_OE_9_peak_922	Solyc04g082030.1
3.99252	IP_OE_9_vs_In_OE_9_peak_923	Solyc04g082150.1
3.92288	IP_OE_9_vs_In_OE_9_peak_924	Solyc04g082200.2
3.4235	IP_OE_9_vs_In_OE_9_peak_925	Solyc04g082250.3
3.64416	IP_OE_9_vs_In_OE_9_peak_926	Solyc04g082460.3
3.35647	IP_OE_9_vs_In_OE_9_peak_927	Solyc04g082500.3
5.76697	IP_OE_9_vs_In_OE_9_peak_928	Solyc04g082710.3
5.79704	IP_OE_9_vs_In_OE_9_peak_929	Solyc04g082910.1
3.70402	IP_OE_9_vs_In_OE_9_peak_930	Solyc04g082970.3
6.59718	IP_OE_9_vs_In_OE_9_peak_931	Solyc04g082990.3
4.56467	IP_OE_9_vs_In_OE_9_peak_932	Solyc05g005000.3
4.58959	IP_OE_9_vs_In_OE_9_peak_933	Solyc05g005010.3
4.87975	IP_OE_9_vs_In_OE_9_peak_934	Solyc05g005050.3
4.63003	IP_OE_9_vs_In_OE_9_peak_935	Solyc05g005090.3
5.27923	IP_OE_9_vs_In_OE_9_peak_936	Solyc05g005160.3
3.49176	IP_OE_9_vs_In_OE_9_peak_937	Solyc05g005170.3
5.76697	IP_OE_9_vs_In_OE_9_peak_938	Solyc05g005280.3

4.43613	IP_OE_9_vs_In_OE_9_peak_939	Solyc05g005490.3
4.87717	IP_OE_9_vs_In_OE_9_peak_940	Solyc05g005560.4
5.76697	IP_OE_9_vs_In_OE_9_peak_941	Solyc05g005670.1
4.92544	IP_OE_9_vs_In_OE_9_peak_942	Solyc05g005680.3
5.32336	IP_OE_9_vs_In_OE_9_peak_943	Solyc05g005735.1
4.05384	IP_OE_9_vs_In_OE_9_peak_944	Solyc05g005760.3
6.189	IP_OE_9_vs_In_OE_9_peak_945	Solyc05g005790.3
4.43613	IP_OE_9_vs_In_OE_9_peak_946	Solyc05g005960.3
4.87975	IP_OE_9_vs_In_OE_9_peak_947	Solyc05g006050.3
4.126	IP_OE_9_vs_In_OE_9_peak_948	Solyc05g006343.1
3.39535	IP_OE_9_vs_In_OE_9_peak_949	Solyc05g006400.2
6.6542	IP_OE_9_vs_In_OE_9_peak_950	Solyc05g006510.1
3.99252	IP_OE_9_vs_In_OE_9_peak_951	Solyc05g006960.3
5.32544	IP_OE_9_vs_In_OE_9_peak_952	Solyc05g007150.3
6.21059	IP_OE_9_vs_In_OE_9_peak_953	Solyc05g007280.3
4.43613	IP_OE_9_vs_In_OE_9_peak_954	Solyc05g007830.3
5.32336	IP_OE_9_vs_In_OE_9_peak_955	Solyc05g007880.3
3.99252	IP_OE_9_vs_In_OE_9_peak_956	Solyc05g008060.3
4.87975	IP_OE_9_vs_In_OE_9_peak_957	Solyc05g008070.3
4.53556	IP_OE_9_vs_In_OE_9_peak_958	Solyc05g008120.3
4.87975	IP_OE_9_vs_In_OE_9_peak_959	Solyc05g008570.2
5.76697	IP_OE_9_vs_In_OE_9_peak_960	Solyc05g009000.3
7.54143	IP_OE_9_vs_In_OE_9_peak_961	Solyc05g009110.1
5.10289	IP_OE_9_vs_In_OE_9_peak_962	Solyc05g009170.2
4.43613	IP_OE_9_vs_In_OE_9_peak_963	Solyc05g009180.1
5.32336	IP_OE_9_vs_In_OE_9_peak_964	Solyc05g009320.3
4.53556	IP_OE_9_vs_In_OE_9_peak_965	Solyc05g009340.1
3.99252	IP_OE_9_vs_In_OE_9_peak_966	Solyc05g009360.3
4.16768	IP_OE_9_vs_In_OE_9_peak_967	Solyc05g009490.2
5.7764	IP_OE_9_vs_In_OE_9_peak_968	Solyc05g009650.3
4.43613	IP_OE_9_vs_In_OE_9_peak_969	Solyc05g009680.1
5.32336	IP_OE_9_vs_In_OE_9_peak_970	Solyc05g009780.3
3.29369	IP_OE_9_vs_In_OE_9_peak_971	Solyc05g009790.1
3.54891	IP_OE_9_vs_In_OE_9_peak_972	Solyc05g009840.3
3.54891	IP_OE_9_vs_In_OE_9_peak_973	Solyc05g009900.2
5.76697	IP_OE_9_vs_In_OE_9_peak_974	Solyc05g009930.3
4.87975	IP_OE_9_vs_In_OE_9_peak_975	Solyc05g010000.1
4.43613	IP_OE_9_vs_In_OE_9_peak_976	Solyc05g010120.3
4.35876	IP_OE_9_vs_In_OE_9_peak_977	Solyc05g010700.1
4.70614	IP_OE_9_vs_In_OE_9_peak_978	Solyc05g010710.2
4.56467	IP_OE_9_vs_In_OE_9_peak_979	Solyc05g011930.3
5.76697	IP_OE_9_vs_In_OE_9_peak_980	Solyc05g012320.1
4.43613	IP_OE_9_vs_In_OE_9_peak_981	Solyc05g012450.3
5.32336	IP_OE_9_vs_In_OE_9_peak_982	Solyc05g012580.1
4.94053	IP_OE_9_vs_In_OE_9_peak_983	Solyc05g013420.1
3.99252	IP_OE_9_vs_In_OE_9_peak_984	Solyc05g013460.3
4.76774	IP_OE_9_vs_In_OE_9_peak_985	Solyc05g013530.3

5.32336	IP_OE_9_vs_In_OE_9_peak_986	Solyc05g013580.3
5.32336	IP_OE_9_vs_In_OE_9_peak_987	Solyc05g013670.3
4.43613	IP_OE_9_vs_In_OE_9_peak_988	Solyc05g013680.3
3.99252	IP_OE_9_vs_In_OE_9_peak_989	Solyc05g013690.3
5.32336	IP_OE_9_vs_In_OE_9_peak_990	Solyc05g013760.3
4.47344	IP_OE_9_vs_In_OE_9_peak_991	Solyc05g014000.3
5.21091	IP_OE_9_vs_In_OE_9_peak_992	Solyc05g015290.1
5.76697	IP_OE_9_vs_In_OE_9_peak_993	Solyc05g015390.3
6.41879	IP_OE_9_vs_In_OE_9_peak_994	Solyc05g015420.3
3.54026	IP_OE_9_vs_In_OE_9_peak_995	Solyc05g015430.1
5.32336	IP_OE_9_vs_In_OE_9_peak_996	Solyc05g015690.1
4.94506	IP_OE_9_vs_In_OE_9_peak_997	Solyc05g015810.2
4.87975	IP_OE_9_vs_In_OE_9_peak_998	Solyc05g015840.3
5.76697	IP_OE_9_vs_In_OE_9_peak_999	Solyc05g016120.2
5.1364	IP_OE_9_vs_In_OE_9_peak_1000	Solyc05g016185.1
4.53556	IP_OE_9_vs_In_OE_9_peak_1001	Solyc05g016310.1
3.80389	IP_OE_9_vs_In_OE_9_peak_1002	Solyc05g016580.2
3.53045	IP_OE_9_vs_In_OE_9_peak_1003	Solyc05g017740.1
3.99252	IP_OE_9_vs_In_OE_9_peak_1004	Solyc05g018120.1
4.78563	IP_OE_9_vs_In_OE_9_peak_1005	Solyc05g018230.3
3.54891	IP_OE_9_vs_In_OE_9_peak_1006	Solyc05g019980.2
4.87975	IP_OE_9_vs_In_OE_9_peak_1007	Solyc05g021090.3
3.99252	IP_OE_9_vs_In_OE_9_peak_1008	Solyc05g023670.2
4.94263	IP_OE_9_vs_In_OE_9_peak_1009	Solyc05g023720.1
3.29369	IP_OE_9_vs_In_OE_9_peak_1010	Solyc05g023900.1
4.20077	IP_OE_9_vs_In_OE_9_peak_1011	Solyc05g025580.1
4.87975	IP_OE_9_vs_In_OE_9_peak_1012	Solyc05g025820.3
5.26999	IP_OE_9_vs_In_OE_9_peak_1013	Solyc05g025870.3
4.87975	IP_OE_9_vs_In_OE_9_peak_1014	Solyc05g026335.1
10.02514	IP_OE_9_vs_In_OE_9_peak_1015	Solyc05g041360.2
5.32544	IP_OE_9_vs_In_OE_9_peak_1016	Solyc05g041870.1
5.32544	IP_OE_9_vs_In_OE_9_peak_1017	Solyc05g041910.3
3.89359	IP_OE_9_vs_In_OE_9_peak_1018	Solyc05g043240.1
4.92544	IP_OE_9_vs_In_OE_9_peak_1019	Solyc05g047590.3
7.54143	IP_OE_9_vs_In_OE_9_peak_1020	Solyc05g050230.3
7.22739	IP_OE_9_vs_In_OE_9_peak_1021	Solyc05g050380.3
4.43613	IP_OE_9_vs_In_OE_9_peak_1022	Solyc05g050770.3
4.85859	IP_OE_9_vs_In_OE_9_peak_1023	Solyc05g051040.3
6.60395	IP_OE_9_vs_In_OE_9_peak_1024	Solyc05g051070.3
4.781	IP_OE_9_vs_In_OE_9_peak_1025	Solyc05g051200.1
4.56467	IP_OE_9_vs_In_OE_9_peak_1026	Solyc05g051290.3
3.99252	IP_OE_9_vs_In_OE_9_peak_1027	Solyc05g051400.3
4.12324	IP_OE_9_vs_In_OE_9_peak_1028	Solyc05g051460.3
4.56467	IP_OE_9_vs_In_OE_9_peak_1029	Solyc05g051470.2
5.76697	IP_OE_9_vs_In_OE_9_peak_1030	Solyc05g051550.2
4.56467	IP_OE_9_vs_In_OE_9_peak_1031	Solyc05g051690.3
4.94789	IP_OE_9_vs_In_OE_9_peak_1032	Solyc05g051810.1

4.64666	IP_OE_9_vs_In_OE_9_peak_1033	Solyc05g051900.3
7.09781	IP_OE_9_vs_In_OE_9_peak_1034	Solyc05g052030.1
3.99252	IP_OE_9_vs_In_OE_9_peak_1035	Solyc05g052050.1
4.87975	IP_OE_9_vs_In_OE_9_peak_1036	Solyc05g052520.3
5.58241	IP_OE_9_vs_In_OE_9_peak_1037	Solyc05g052550.1
4.35876	IP_OE_9_vs_In_OE_9_peak_1038	Solyc05g052600.3
4.43613	IP_OE_9_vs_In_OE_9_peak_1039	Solyc05g052810.3
3.99252	IP_OE_9_vs_In_OE_9_peak_1040	Solyc05g052980.3
4.87975	IP_OE_9_vs_In_OE_9_peak_1041	Solyc05g053070.3
5.19146	IP_OE_9_vs_In_OE_9_peak_1042	Solyc05g053210.3
4.56467	IP_OE_9_vs_In_OE_9_peak_1043	Solyc05g053340.3
7.09781	IP_OE_9_vs_In_OE_9_peak_1044	Solyc05g053500.3
7.84576	IP_OE_9_vs_In_OE_9_peak_1045	Solyc05g053530.1
5.6346	IP_OE_9_vs_In_OE_9_peak_1046	Solyc05g053550.3
5.30712	IP_OE_9_vs_In_OE_9_peak_1047	Solyc05g053760.3
3.485	IP_OE_9_vs_In_OE_9_peak_1048	Solyc05g053780.3
4.61116	IP_OE_9_vs_In_OE_9_peak_1049	Solyc05g054030.3
4.38349	IP_OE_9_vs_In_OE_9_peak_1050	Solyc05g054390.3
5.36021	IP_OE_9_vs_In_OE_9_peak_1051	Solyc05g054440.3
6.6542	IP_OE_9_vs_In_OE_9_peak_1052	Solyc05g054730.3
4.56467	IP_OE_9_vs_In_OE_9_peak_1053	Solyc05g054880.3
3.54891	IP_OE_9_vs_In_OE_9_peak_1054	Solyc05g055400.3
5.29568	IP_OE_9_vs_In_OE_9_peak_1055	Solyc05g055440.1
3.95242	IP_OE_9_vs_In_OE_9_peak_1056	Solyc05g056130.3
3.99252	IP_OE_9_vs_In_OE_9_peak_1057	Solyc06g005090.3
3.5268	IP_OE_9_vs_In_OE_9_peak_1058	Solyc06g005150.3
4.9512	IP_OE_9_vs_In_OE_9_peak_1059	Solyc06g005390.1
3.99252	IP_OE_9_vs_In_OE_9_peak_1060	Solyc06g005500.3
6.59718	IP_OE_9_vs_In_OE_9_peak_1061	Solyc06g005520.3
2.66051	IP_OE_9_vs_In_OE_9_peak_1062	Solyc06g005560.3
5.76697	IP_OE_9_vs_In_OE_9_peak_1063	Solyc06g007350.3
5.20117	IP_OE_9_vs_In_OE_9_peak_1064	Solyc06g008830.1
3.8835	IP_OE_9_vs_In_OE_9_peak_1065	Solyc06g008880.3
4.56467	IP_OE_9_vs_In_OE_9_peak_1066	Solyc06g008890.3
6.21059	IP_OE_9_vs_In_OE_9_peak_1067	Solyc06g008940.3
7.78719	IP_OE_9_vs_In_OE_9_peak_1068	Solyc06g009340.3
5.32336	IP_OE_9_vs_In_OE_9_peak_1069	Solyc06g009380.3
4.53556	IP_OE_9_vs_In_OE_9_peak_1070	Solyc06g009520.3
3.4235	IP_OE_9_vs_In_OE_9_peak_1071	Solyc06g010050.1
3.90781	IP_OE_9_vs_In_OE_9_peak_1072	Solyc06g024240.1
6.16018	IP_OE_9_vs_In_OE_9_peak_1073	Solyc06g016765.1
6.18486	IP_OE_9_vs_In_OE_9_peak_1074	Solyc06g017910.1
4.70614	IP_OE_9_vs_In_OE_9_peak_1075	Solyc06g030630.1
4.40013	IP_OE_9_vs_In_OE_9_peak_1076	Solyc06g033980.2
6.73344	IP_OE_9_vs_In_OE_9_peak_1077	Solyc06g035560.2
4.76774	IP_OE_9_vs_In_OE_9_peak_1078	Solyc06g035690.3
4.43613	IP_OE_9_vs_In_OE_9_peak_1079	Solyc06g035960.3

5.88108	IP_OE_9_vs_In_OE_9_peak_1080	Solyc06g036130.3
4.43613	IP_OE_9_vs_In_OE_9_peak_1081	Solyc06g036170.1
4.43613	IP_OE_9_vs_In_OE_9_peak_1082	Solyc06g036210.1
5.76614	IP_OE_9_vs_In_OE_9_peak_1083	Solyc06g036770.1
3.99252	IP_OE_9_vs_In_OE_9_peak_1084	Solyc06g048637.1
7.09781	IP_OE_9_vs_In_OE_9_peak_1085	Solyc06g049040.3
5.76697	IP_OE_9_vs_In_OE_9_peak_1086	Solyc06g049050.3
4.23654	IP_OE_9_vs_In_OE_9_peak_1087	Solyc06g050130.3
4.23654	IP_OE_9_vs_In_OE_9_peak_1088	Solyc06g050260.1
6.08622	IP_OE_9_vs_In_OE_9_peak_1089	Solyc06g050370.1
4.23654	IP_OE_9_vs_In_OE_9_peak_1090	Solyc06g050440.3
5.25025	IP_OE_9_vs_In_OE_9_peak_1091	Solyc06g050590.3
5.95665	IP_OE_9_vs_In_OE_9_peak_1092	Solyc06g050700.3
4.18428	IP_OE_9_vs_In_OE_9_peak_1093	Solyc06g050720.3
5.32336	IP_OE_9_vs_In_OE_9_peak_1094	Solyc06g050980.3
4.55331	IP_OE_9_vs_In_OE_9_peak_1095	Solyc06g051460.3
3.47394	IP_OE_9_vs_In_OE_9_peak_1096	Solyc06g051750.3
5.76697	IP_OE_9_vs_In_OE_9_peak_1097	Solyc06g051850.2
3.70402	IP_OE_9_vs_In_OE_9_peak_1098	Solyc06g051920.3
3.91684	IP_OE_9_vs_In_OE_9_peak_1099	Solyc06g052020.2
4.94506	IP_OE_9_vs_In_OE_9_peak_1100	Solyc06g053240.3
3.99252	IP_OE_9_vs_In_OE_9_peak_1101	Solyc06g053640.1
5.04093	IP_OE_9_vs_In_OE_9_peak_1102	Solyc06g053653.1
5.76697	IP_OE_9_vs_In_OE_9_peak_1103	Solyc06g053720.2
4.87975	IP_OE_9_vs_In_OE_9_peak_1104	Solyc06g053810.3
6.17189	IP_OE_9_vs_In_OE_9_peak_1105	Solyc06g053840.3
4.53556	IP_OE_9_vs_In_OE_9_peak_1106	Solyc06g059800.3
5.53117	IP_OE_9_vs_In_OE_9_peak_1107	Solyc06g059870.1
5.76697	IP_OE_9_vs_In_OE_9_peak_1108	Solyc06g060110.3
4.50651	IP_OE_9_vs_In_OE_9_peak_1109	Solyc06g060120.3
5.36021	IP_OE_9_vs_In_OE_9_peak_1110	Solyc06g060310.3
3.80389	IP_OE_9_vs_In_OE_9_peak_1111	Solyc06g060340.3
5.13397	IP_OE_9_vs_In_OE_9_peak_1112	Solyc06g060460.3
4.87975	IP_OE_9_vs_In_OE_9_peak_1113	Solyc06g060500.1
4.58959	IP_OE_9_vs_In_OE_9_peak_1114	Solyc06g060520.1
4.18428	IP_OE_9_vs_In_OE_9_peak_1115	Solyc06g060610.2
4.63766	IP_OE_9_vs_In_OE_9_peak_1116	Solyc06g060680.2
4.53556	IP_OE_9_vs_In_OE_9_peak_1117	Solyc06g061030.3
4.87975	IP_OE_9_vs_In_OE_9_peak_1118	Solyc06g061240.3
4.62085	IP_OE_9_vs_In_OE_9_peak_1119	Solyc06g061260.1
6.189	IP_OE_9_vs_In_OE_9_peak_1120	Solyc06g062680.2
3.80389	IP_OE_9_vs_In_OE_9_peak_1121	Solyc06g062950.1
4.87975	IP_OE_9_vs_In_OE_9_peak_1122	Solyc06g063120.3
6.6542	IP_OE_9_vs_In_OE_9_peak_1123	Solyc06g063295.1
6.42933	IP_OE_9_vs_In_OE_9_peak_1124	Solyc06g064450.3
4.43613	IP_OE_9_vs_In_OE_9_peak_1125	Solyc06g064460.3
3.41526	IP_OE_9_vs_In_OE_9_peak_1126	Solyc06g064870.3

6.21059	IP_OE_9_vs_In_OE_9_peak_1127	Solyc06g065040.3
4.87975	IP_OE_9_vs_In_OE_9_peak_1128	Solyc06g065190.1
5.32336	IP_OE_9_vs_In_OE_9_peak_1129	Solyc06g065260.3
4.43613	IP_OE_9_vs_In_OE_9_peak_1130	Solyc06g065670.3
4.70021	IP_OE_9_vs_In_OE_9_peak_1131	Solyc06g065690.3
4.35876	IP_OE_9_vs_In_OE_9_peak_1132	Solyc06g065820.3
4.30852	IP_OE_9_vs_In_OE_9_peak_1133	Solyc06g065890.1
4.26257	IP_OE_9_vs_In_OE_9_peak_1134	Solyc06g084820.2
6.21059	IP_OE_9_vs_In_OE_9_peak_1135	Solyc06g066340.3
5.32336	IP_OE_9_vs_In_OE_9_peak_1136	Solyc06g066370.3
4.48274	IP_OE_9_vs_In_OE_9_peak_1137	Solyc06g066570.3
4.54656	IP_OE_9_vs_In_OE_9_peak_1138	Solyc06g066650.3
6.17189	IP_OE_9_vs_In_OE_9_peak_1139	Solyc06g068140.3
4.43613	IP_OE_9_vs_In_OE_9_peak_1140	Solyc06g068460.3
3.99252	IP_OE_9_vs_In_OE_9_peak_1141	Solyc06g068620.3
3.99252	IP_OE_9_vs_In_OE_9_peak_1142	Solyc06g068650.3
4.10704	IP_OE_9_vs_In_OE_9_peak_1143	Solyc06g068840.3
4.23654	IP_OE_9_vs_In_OE_9_peak_1144	Solyc06g069060.1
3.99252	IP_OE_9_vs_In_OE_9_peak_1145	Solyc06g069385.1
3.99252	IP_OE_9_vs_In_OE_9_peak_1146	Solyc06g069565.1
6.18486	IP_OE_9_vs_In_OE_9_peak_1147	Solyc06g069697.1
4.87975	IP_OE_9_vs_In_OE_9_peak_1148	Solyc06g069760.3
4.56467	IP_OE_9_vs_In_OE_9_peak_1149	Solyc06g069770.3
3.99252	IP_OE_9_vs_In_OE_9_peak_1150	Solyc06g069860.3
4.781	IP_OE_9_vs_In_OE_9_peak_1151	Solyc06g070900.3
4.43613	IP_OE_9_vs_In_OE_9_peak_1152	Solyc06g070990.3
2.52685	IP_OE_9_vs_In_OE_9_peak_1153	Solyc06g071100.3
2.16094	IP_OE_9_vs_In_OE_9_peak_1154	Solyc06g071285.1
1.81596	IP_OE_9_vs_In_OE_9_peak_1155	Solyc06g071420.3
1.43053	IP_OE_9_vs_In_OE_9_peak_1156	Solyc06g071510.3
6.6542	IP_OE_9_vs_In_OE_9_peak_1157	Solyc06g071805.1
4.38258	IP_OE_9_vs_In_OE_9_peak_1158	Solyc06g071810.1
3.54891	IP_OE_9_vs_In_OE_9_peak_1159	Solyc06g071890.3
2.86534	IP_OE_9_vs_In_OE_9_peak_1160	Solyc06g071950.2
5.32336	IP_OE_9_vs_In_OE_9_peak_1161	Solyc06g072018.1
6.6542	IP_OE_9_vs_In_OE_9_peak_1162	Solyc06g072690.2
4.87975	IP_OE_9_vs_In_OE_9_peak_1163	Solyc06g072710.3
4.77667	IP_OE_9_vs_In_OE_9_peak_1164	Solyc06g073050.2
5.87526	IP_OE_9_vs_In_OE_9_peak_1165	Solyc06g073200.3
4.94506	IP_OE_9_vs_In_OE_9_peak_1166	Solyc06g073245.1
6.6542	IP_OE_9_vs_In_OE_9_peak_1167	Solyc06g073620.3
4.87975	IP_OE_9_vs_In_OE_9_peak_1168	Solyc06g073860.2
4.12324	IP_OE_9_vs_In_OE_9_peak_1169	Solyc06g074040.1
4.87975	IP_OE_9_vs_In_OE_9_peak_1170	Solyc06g074120.3
3.54891	IP_OE_9_vs_In_OE_9_peak_1171	Solyc06g074350.3
5.09189	IP_OE_9_vs_In_OE_9_peak_1172	Solyc06g074600.1
4.43379	IP_OE_9_vs_In_OE_9_peak_1173	Solyc06g074680.3

3.54891	IP_OE_9_vs_In_OE_9_peak_1174	Solyc06g074810.3
5.36021	IP_OE_9_vs_In_OE_9_peak_1175	Solyc06g074930.2
4.18428	IP_OE_9_vs_In_OE_9_peak_1176	Solyc06g075130.3
5.00728	IP_OE_9_vs_In_OE_9_peak_1177	Solyc06g075180.1
3.29369	IP_OE_9_vs_In_OE_9_peak_1178	Solyc06g075567.1
5.46101	IP_OE_9_vs_In_OE_9_peak_1179	Solyc06g075610.1
5.36021	IP_OE_9_vs_In_OE_9_peak_1180	Solyc06g075690.3
5.32544	IP_OE_9_vs_In_OE_9_peak_1181	Solyc06g075960.1
3.16664	IP_OE_9_vs_In_OE_9_peak_1182	Solyc06g076090.3
3.8835	IP_OE_9_vs_In_OE_9_peak_1183	Solyc06g076100.3
5.17941	IP_OE_9_vs_In_OE_9_peak_1184	Solyc06g076130.3
3.53045	IP_OE_9_vs_In_OE_9_peak_1185	Solyc06g076290.1
3.99252	IP_OE_9_vs_In_OE_9_peak_1186	Solyc06g076400.3
3.71092	IP_OE_9_vs_In_OE_9_peak_1187	Solyc06g076490.3
4.87975	IP_OE_9_vs_In_OE_9_peak_1188	Solyc06g076510.3
4.79463	IP_OE_9_vs_In_OE_9_peak_1189	Solyc06g076750.3
6.40564	IP_OE_9_vs_In_OE_9_peak_1190	Solyc06g076760.2
5.32336	IP_OE_9_vs_In_OE_9_peak_1191	Solyc06g076800.3
4.87975	IP_OE_9_vs_In_OE_9_peak_1192	Solyc06g076840.2
4.87975	IP_OE_9_vs_In_OE_9_peak_1193	Solyc06g076850.3
4.68883	IP_OE_9_vs_In_OE_9_peak_1194	Solyc06g082010.3
3.54891	IP_OE_9_vs_In_OE_9_peak_1195	Solyc06g082020.3
4.18428	IP_OE_9_vs_In_OE_9_peak_1196	Solyc06g082210.1
3.54891	IP_OE_9_vs_In_OE_9_peak_1197	Solyc06g082220.3
3.99252	IP_OE_9_vs_In_OE_9_peak_1198	Solyc06g082310.3
5.23051	IP_OE_9_vs_In_OE_9_peak_1199	Solyc06g082410.1
4.43613	IP_OE_9_vs_In_OE_9_peak_1200	Solyc06g082530.2
6.21059	IP_OE_9_vs_In_OE_9_peak_1201	Solyc06g082570.2
4.18428	IP_OE_9_vs_In_OE_9_peak_1202	Solyc06g082590.1
5.70583	IP_OE_9_vs_In_OE_9_peak_1203	Solyc06g082950.3
3.99252	IP_OE_9_vs_In_OE_9_peak_1204	Solyc06g083210.3
4.12324	IP_OE_9_vs_In_OE_9_peak_1205	Solyc06g083310.3
3.92347	IP_OE_9_vs_In_OE_9_peak_1206	Solyc06g083390.3
4.93364	IP_OE_9_vs_In_OE_9_peak_1207	Solyc06g083650.3
4.50651	IP_OE_9_vs_In_OE_9_peak_1208	Solyc06g083680.3
6.00177	IP_OE_9_vs_In_OE_9_peak_1209	Solyc06g083930.2
4.43613	IP_OE_9_vs_In_OE_9_peak_1210	Solyc06g084070.3
6.21059	IP_OE_9_vs_In_OE_9_peak_1211	Solyc06g084430.3
5.76697	IP_OE_9_vs_In_OE_9_peak_1212	Solyc07g004993.1
3.4235	IP_OE_9_vs_In_OE_9_peak_1213	Solyc07g005090.3
4.35058	IP_OE_9_vs_In_OE_9_peak_1214	Solyc07g005400.3
4.33431	IP_OE_9_vs_In_OE_9_peak_1215	Solyc07g005410.3
3.99252	IP_OE_9_vs_In_OE_9_peak_1216	Solyc07g005420.2
3.39535	IP_OE_9_vs_In_OE_9_peak_1217	Solyc07g005450.3
7.09781	IP_OE_9_vs_In_OE_9_peak_1218	Solyc07g005700.3
4.30213	IP_OE_9_vs_In_OE_9_peak_1219	Solyc07g005765.1
4.87975	IP_OE_9_vs_In_OE_9_peak_1220	Solyc07g005960.3

4.43613	IP_OE_9_vs_In_OE_9_peak_1221	Solyc07g006090.2
3.88634	IP_OE_9_vs_In_OE_9_peak_1222	Solyc07g006220.2
4.18428	IP_OE_9_vs_In_OE_9_peak_1223	Solyc07g006310.1
2.86541	IP_OE_9_vs_In_OE_9_peak_1224	Solyc07g006370.1
2.54875	IP_OE_9_vs_In_OE_9_peak_1225	Solyc07g006420.1
4.85859	IP_OE_9_vs_In_OE_9_peak_1226	Solyc07g006480.3
1.92921	IP_OE_9_vs_In_OE_9_peak_1227	Solyc07g006540.3
5.70933	IP_OE_9_vs_In_OE_9_peak_1228	Solyc07g006770.2
4.58959	IP_OE_9_vs_In_OE_9_peak_1229	Solyc07g007160.3
4.30852	IP_OE_9_vs_In_OE_9_peak_1230	Solyc07g007170.3
4.63003	IP_OE_9_vs_In_OE_9_peak_1231	Solyc07g007320.2
5.76697	IP_OE_9_vs_In_OE_9_peak_1232	Solyc07g007540.1
5.04093	IP_OE_9_vs_In_OE_9_peak_1233	Solyc07g007630.3
3.19639	IP_OE_9_vs_In_OE_9_peak_1234	Solyc07g007950.1
4.87975	IP_OE_9_vs_In_OE_9_peak_1235	Solyc07g007980.3
6.6542	IP_OE_9_vs_In_OE_9_peak_1236	Solyc07g008250.3
4.94506	IP_OE_9_vs_In_OE_9_peak_1237	Solyc07g008280.3
4.87717	IP_OE_9_vs_In_OE_9_peak_1238	Solyc07g008630.1
3.99252	IP_OE_9_vs_In_OE_9_peak_1239	Solyc07g009110.2
3.99252	IP_OE_9_vs_In_OE_9_peak_1240	Solyc07g016200.3
5.04093	IP_OE_9_vs_In_OE_9_peak_1241	Solyc07g017777.1
4.32136	IP_OE_9_vs_In_OE_9_peak_1242	Solyc07g017780.3
6.59718	IP_OE_9_vs_In_OE_9_peak_1243	Solyc07g018190.3
4.38258	IP_OE_9_vs_In_OE_9_peak_1244	Solyc07g018387.1
4.77667	IP_OE_9_vs_In_OE_9_peak_1245	Solyc07g020790.3
4.18428	IP_OE_9_vs_In_OE_9_peak_1246	Solyc07g021700.3
4.27043	IP_OE_9_vs_In_OE_9_peak_1247	Solyc07g022810.1
6.0794	IP_OE_9_vs_In_OE_9_peak_1248	Solyc07g024040.2
4.20077	IP_OE_9_vs_In_OE_9_peak_1249	Solyc07g026680.2
4.12324	IP_OE_9_vs_In_OE_9_peak_1250	Solyc07g032050.1
4.56467	IP_OE_9_vs_In_OE_9_peak_1251	Solyc07g032110.3
5.04093	IP_OE_9_vs_In_OE_9_peak_1252	Solyc07g032450.1
4.38258	IP_OE_9_vs_In_OE_9_peak_1253	Solyc07g039250.1
4.43613	IP_OE_9_vs_In_OE_9_peak_1254	Solyc07g039570.3
5.76697	IP_OE_9_vs_In_OE_9_peak_1255	Solyc07g040800.1
5.70583	IP_OE_9_vs_In_OE_9_peak_1256	Solyc07g041213.1
5.60317	IP_OE_9_vs_In_OE_9_peak_1257	Solyc07g041620.1
5.15299	IP_OE_9_vs_In_OE_9_peak_1258	Solyc07g041950.1
5.32336	IP_OE_9_vs_In_OE_9_peak_1259	Solyc07g042130.3
4.53556	IP_OE_9_vs_In_OE_9_peak_1260	Solyc07g042150.3
3.97521	IP_OE_9_vs_In_OE_9_peak_1261	Solyc07g042160.3
4.74997	IP_OE_9_vs_In_OE_9_peak_1262	Solyc07g042170.3
4.94789	IP_OE_9_vs_In_OE_9_peak_1263	Solyc07g042190.3
3.99252	IP_OE_9_vs_In_OE_9_peak_1264	Solyc07g042270.3
4.43613	IP_OE_9_vs_In_OE_9_peak_1265	Solyc07g042790.2
4.20077	IP_OE_9_vs_In_OE_9_peak_1266	Solyc07g043000.3
7.09781	IP_OE_9_vs_In_OE_9_peak_1267	Solyc07g043130.3

5.07774	IP_OE_9_vs_In_OE_9_peak_1268	Solyc07g044710.2
4.12324	IP_OE_9_vs_In_OE_9_peak_1269	Solyc07g044860.3
4.5386	IP_OE_9_vs_In_OE_9_peak_1270	Solyc07g045000.3
4.86786	IP_OE_9_vs_In_OE_9_peak_1271	Solyc07g045030.3
4.93364	IP_OE_9_vs_In_OE_9_peak_1272	Solyc07g045450.1
6.21059	IP_OE_9_vs_In_OE_9_peak_1273	Solyc07g045530.2
6.24458	IP_OE_9_vs_In_OE_9_peak_1274	Solyc07g047780.3
4.68022	IP_OE_9_vs_In_OE_9_peak_1275	Solyc07g047850.3
4.94506	IP_OE_9_vs_In_OE_9_peak_1276	Solyc07g047960.3
5.14346	IP_OE_9_vs_In_OE_9_peak_1277	Solyc07g047970.3
4.72357	IP_OE_9_vs_In_OE_9_peak_1278	Solyc07g049220.3
7.54143	IP_OE_9_vs_In_OE_9_peak_1279	Solyc07g049370.2
4.87975	IP_OE_9_vs_In_OE_9_peak_1280	Solyc07g049385.1
5.69853	IP_OE_9_vs_In_OE_9_peak_1281	Solyc07g049520.2
4.92544	IP_OE_9_vs_In_OE_9_peak_1282	Solyc07g049530.3
6.51364	IP_OE_9_vs_In_OE_9_peak_1283	Solyc07g049640.3
4.94789	IP_OE_9_vs_In_OE_9_peak_1284	Solyc07g051840.3
4.87975	IP_OE_9_vs_In_OE_9_peak_1285	Solyc07g052490.3
4.43613	IP_OE_9_vs_In_OE_9_peak_1286	Solyc07g052650.2
4.74114	IP_OE_9_vs_In_OE_9_peak_1287	Solyc07g053110.3
5.27015	IP_OE_9_vs_In_OE_9_peak_1288	Solyc07g053140.3
3.64846	IP_OE_9_vs_In_OE_9_peak_1289	Solyc07g053200.3
5.46101	IP_OE_9_vs_In_OE_9_peak_1290	Solyc07g053220.2
3.99252	IP_OE_9_vs_In_OE_9_peak_1291	Solyc07g053300.1
3.99252	IP_OE_9_vs_In_OE_9_peak_1292	Solyc07g053420.3
5.68319	IP_OE_9_vs_In_OE_9_peak_1293	Solyc07g053700.3
5.76697	IP_OE_9_vs_In_OE_9_peak_1294	Solyc07g053740.1
5.19146	IP_OE_9_vs_In_OE_9_peak_1295	Solyc07g053970.3
4.79463	IP_OE_9_vs_In_OE_9_peak_1296	Solyc07g054220.1
6.03852	IP_OE_9_vs_In_OE_9_peak_1297	Solyc07g054280.1
4.64666	IP_OE_9_vs_In_OE_9_peak_1298	Solyc07g054720.2
4.31816	IP_OE_9_vs_In_OE_9_peak_1299	Solyc07g054800.1
5.05927	IP_OE_9_vs_In_OE_9_peak_1300	Solyc07g054950.2
3.14546	IP_OE_9_vs_In_OE_9_peak_1301	Solyc07g054960.2
4.43613	IP_OE_9_vs_In_OE_9_peak_1302	Solyc07g055210.3
3.93027	IP_OE_9_vs_In_OE_9_peak_1303	Solyc07g055560.3
4.53556	IP_OE_9_vs_In_OE_9_peak_1304	Solyc07g055670.1
6.847	IP_OE_9_vs_In_OE_9_peak_1305	Solyc07g055940.2
3.99252	IP_OE_9_vs_In_OE_9_peak_1306	Solyc07g056000.2
4.56467	IP_OE_9_vs_In_OE_9_peak_1307	Solyc07g056150.3
7.98817	IP_OE_9_vs_In_OE_9_peak_1308	Solyc07g056210.3
4.58959	IP_OE_9_vs_In_OE_9_peak_1309	Solyc07g056360.1
4.58959	IP_OE_9_vs_In_OE_9_peak_1310	Solyc07g056380.1
3.8835	IP_OE_9_vs_In_OE_9_peak_1311	Solyc07g056400.1
7.11039	IP_OE_9_vs_In_OE_9_peak_1312	Solyc07g056450.3
4.36697	IP_OE_9_vs_In_OE_9_peak_1313	Solyc07g056600.1
4.14049	IP_OE_9_vs_In_OE_9_peak_1314	Solyc07g061700.1

4.12324	IP_OE_9_vs_In_OE_9_peak_1315	Solyc07g061770.2
8.252	IP_OE_9_vs_In_OE_9_peak_1316	Solyc07g061790.3
7.42183	IP_OE_9_vs_In_OE_9_peak_1317	Solyc07g062250.3
3.8835	IP_OE_9_vs_In_OE_9_peak_1318	Solyc07g062270.3
4.70614	IP_OE_9_vs_In_OE_9_peak_1319	Solyc07g062610.3
4.87975	IP_OE_9_vs_In_OE_9_peak_1320	Solyc07g062660.3
4.23654	IP_OE_9_vs_In_OE_9_peak_1321	Solyc07g062680.2
4.71484	IP_OE_9_vs_In_OE_9_peak_1322	Solyc07g062740.3
5.76697	IP_OE_9_vs_In_OE_9_peak_1323	Solyc07g063070.1
6.35481	IP_OE_9_vs_In_OE_9_peak_1324	Solyc07g063350.3
4.18428	IP_OE_9_vs_In_OE_9_peak_1325	Solyc07g063440.3
4.43613	IP_OE_9_vs_In_OE_9_peak_1326	Solyc07g063460.2
5.32336	IP_OE_9_vs_In_OE_9_peak_1327	Solyc07g063470.1
2.48688	IP_OE_9_vs_In_OE_9_peak_1328	Solyc07g063770.3
4.87975	IP_OE_9_vs_In_OE_9_peak_1329	Solyc07g063830.3
4.87975	IP_OE_9_vs_In_OE_9_peak_1330	Solyc07g063840.1
5.76393	IP_OE_9_vs_In_OE_9_peak_1331	Solyc07g063850.3
2.67153	IP_OE_9_vs_In_OE_9_peak_1332	Solyc07g063860.3
5.5618	IP_OE_9_vs_In_OE_9_peak_1333	Solyc07g063870.3
4.43613	IP_OE_9_vs_In_OE_9_peak_1334	Solyc07g063940.2
4.87975	IP_OE_9_vs_In_OE_9_peak_1335	Solyc07g064620.2
3.74675	IP_OE_9_vs_In_OE_9_peak_1336	Solyc07g064700.3
4.20077	IP_OE_9_vs_In_OE_9_peak_1337	Solyc07g064720.3
5.76697	IP_OE_9_vs_In_OE_9_peak_1338	Solyc07g065260.3
8.3921	IP_OE_9_vs_In_OE_9_peak_1339	Solyc07g065820.3
6.21059	IP_OE_9_vs_In_OE_9_peak_1340	Solyc07g066230.3
4.53556	IP_OE_9_vs_In_OE_9_peak_1341	Solyc07g066260.3
3.99252	IP_OE_9_vs_In_OE_9_peak_1342	Solyc07g066310.3
8.65243	IP_OE_9_vs_In_OE_9_peak_1343	Solyc07g066360.1
8.28164	IP_OE_9_vs_In_OE_9_peak_1344	Solyc07g066550.3
6.93489	IP_OE_9_vs_In_OE_9_peak_1345	Solyc07g066560.1
4.43613	IP_OE_9_vs_In_OE_9_peak_1346	Solyc07g066570.3
3.8835	IP_OE_9_vs_In_OE_9_peak_1347	Solyc08g005020.3
6.6542	IP_OE_9_vs_In_OE_9_peak_1348	Solyc08g005240.2
3.85175	IP_OE_9_vs_In_OE_9_peak_1349	Solyc08g005490.3
5.66638	IP_OE_9_vs_In_OE_9_peak_1350	Solyc08g005610.3
3.83883	IP_OE_9_vs_In_OE_9_peak_1351	Solyc08g005620.3
4.18428	IP_OE_9_vs_In_OE_9_peak_1352	Solyc08g006256.1
5.24736	IP_OE_9_vs_In_OE_9_peak_1353	Solyc08g006320.3
5.15299	IP_OE_9_vs_In_OE_9_peak_1354	Solyc08g006750.3
4.43613	IP_OE_9_vs_In_OE_9_peak_1355	Solyc08g007000.3
3.99252	IP_OE_9_vs_In_OE_9_peak_1356	Solyc08g007120.3
8.87227	IP_OE_9_vs_In_OE_9_peak_1357	Solyc08g007130.3
6.6542	IP_OE_9_vs_In_OE_9_peak_1358	Solyc08g007220.3
3.82927	IP_OE_9_vs_In_OE_9_peak_1359	Solyc08g007820.1
3.91552	IP_OE_9_vs_In_OE_9_peak_1360	Solyc08g008080.1
4.12324	IP_OE_9_vs_In_OE_9_peak_1361	Solyc08g008610.3

3.99252	IP_OE_9_vs_In_OE_9_peak_1362	Solyc08g008620.3
5.76697	IP_OE_9_vs_In_OE_9_peak_1363	Solyc08g014150.3
3.53045	IP_OE_9_vs_In_OE_9_peak_1364	Solyc08g016215.1
3.7134	IP_OE_9_vs_In_OE_9_peak_1365	Solyc08g016440.3
5.59927	IP_OE_9_vs_In_OE_9_peak_1366	Solyc08g036520.2
4.81409	IP_OE_9_vs_In_OE_9_peak_1367	Solyc08g048240.3
3.54891	IP_OE_9_vs_In_OE_9_peak_1368	Solyc08g044265.1
4.12324	IP_OE_9_vs_In_OE_9_peak_1369	Solyc08g041980.3
5.76697	IP_OE_9_vs_In_OE_9_peak_1370	Solyc08g041930.1
3.4235	IP_OE_9_vs_In_OE_9_peak_1371	Solyc08g041890.3
5.57208	IP_OE_9_vs_In_OE_9_peak_1372	Solyc08g041820.3
5.14346	IP_OE_9_vs_In_OE_9_peak_1373	Solyc08g023493.1
2.03928	IP_OE_9_vs_In_OE_9_peak_1374	Solyc08g023320.1
4.53556	IP_OE_9_vs_In_OE_9_peak_1375	Solyc08g022140.1
3.4235	IP_OE_9_vs_In_OE_9_peak_1376	Solyc08g022020.1
6.46661	IP_OE_9_vs_In_OE_9_peak_1377	Solyc08g021830.1
4.87975	IP_OE_9_vs_In_OE_9_peak_1378	Solyc08g021820.3
4.38258	IP_OE_9_vs_In_OE_9_peak_1379	Solyc08g029400.2
5.08702	IP_OE_9_vs_In_OE_9_peak_1380	Solyc08g029343.1
5.32336	IP_OE_9_vs_In_OE_9_peak_1381	Solyc08g028975.1
4.74997	IP_OE_9_vs_In_OE_9_peak_1382	Solyc08g028770.1
5.76697	IP_OE_9_vs_In_OE_9_peak_1383	Solyc08g060970.3
4.32136	IP_OE_9_vs_In_OE_9_peak_1384	Solyc08g061090.3
5.32336	IP_OE_9_vs_In_OE_9_peak_1385	Solyc08g061240.2
4.43613	IP_OE_9_vs_In_OE_9_peak_1386	Solyc08g061570.2
4.45922	IP_OE_9_vs_In_OE_9_peak_1387	Solyc08g061595.1
3.54891	IP_OE_9_vs_In_OE_9_peak_1388	Solyc08g061610.3
6.6542	IP_OE_9_vs_In_OE_9_peak_1389	Solyc08g061910.3
3.71092	IP_OE_9_vs_In_OE_9_peak_1390	Solyc08g062490.3
7.54143	IP_OE_9_vs_In_OE_9_peak_1391	Solyc08g062680.1
4.87975	IP_OE_9_vs_In_OE_9_peak_1392	Solyc08g062930.2
4.58959	IP_OE_9_vs_In_OE_9_peak_1393	Solyc08g065210.1
4.94789	IP_OE_9_vs_In_OE_9_peak_1394	Solyc08g065350.3
4.1541	IP_OE_9_vs_In_OE_9_peak_1395	Solyc08g065417.1
4.23654	IP_OE_9_vs_In_OE_9_peak_1396	Solyc08g065840.3
3.71092	IP_OE_9_vs_In_OE_9_peak_1397	Solyc08g065850.1
4.73234	IP_OE_9_vs_In_OE_9_peak_1398	Solyc08g065860.3
6.6699	IP_OE_9_vs_In_OE_9_peak_1399	Solyc08g066110.3
4.94263	IP_OE_9_vs_In_OE_9_peak_1400	Solyc08g066440.3
4.94263	IP_OE_9_vs_In_OE_9_peak_1401	Solyc08g066510.3
4.6952	IP_OE_9_vs_In_OE_9_peak_1402	Solyc08g066650.3
5.76697	IP_OE_9_vs_In_OE_9_peak_1403	Solyc08g066660.1
5.59179	IP_OE_9_vs_In_OE_9_peak_1404	Solyc08g066840.3
6.19546	IP_OE_9_vs_In_OE_9_peak_1405	Solyc08g067123.1
4.56467	IP_OE_9_vs_In_OE_9_peak_1406	Solyc08g067170.2
3.40424	IP_OE_9_vs_In_OE_9_peak_1407	Solyc08g067320.2
3.4235	IP_OE_9_vs_In_OE_9_peak_1408	Solyc08g067360.3

3.99252	IP_OE_9_vs_In_OE_9_peak_1409	Solyc08g067520.1
2.48642	IP_OE_9_vs_In_OE_9_peak_1410	Solyc08g067550.1
3.91124	IP_OE_9_vs_In_OE_9_peak_1411	Solyc08g067600.2
6.00177	IP_OE_9_vs_In_OE_9_peak_1412	Solyc08g068730.1
3.53352	IP_OE_9_vs_In_OE_9_peak_1413	Solyc08g074495.1
4.61249	IP_OE_9_vs_In_OE_9_peak_1414	Solyc08g074650.3
3.29171	IP_OE_9_vs_In_OE_9_peak_1415	Solyc08g075017.1
3.50679	IP_OE_9_vs_In_OE_9_peak_1416	Solyc08g075120.3
4.12324	IP_OE_9_vs_In_OE_9_peak_1417	Solyc08g075290.2
1.86682	IP_OE_9_vs_In_OE_9_peak_1418	Solyc08g075320.3
3.95261	IP_OE_9_vs_In_OE_9_peak_1419	Solyc08g075705.1
4.70614	IP_OE_9_vs_In_OE_9_peak_1420	Solyc08g075790.3
5.32336	IP_OE_9_vs_In_OE_9_peak_1421	Solyc08g075925.1
4.53556	IP_OE_9_vs_In_OE_9_peak_1422	Solyc08g075950.2
4.43613	IP_OE_9_vs_In_OE_9_peak_1423	Solyc08g075970.3
5.32544	IP_OE_9_vs_In_OE_9_peak_1424	Solyc08g076390.3
4.87975	IP_OE_9_vs_In_OE_9_peak_1425	Solyc08g076520.3
4.94789	IP_OE_9_vs_In_OE_9_peak_1426	Solyc08g076690.2
6.97083	IP_OE_9_vs_In_OE_9_peak_1427	Solyc08g076730.3
4.43613	IP_OE_9_vs_In_OE_9_peak_1428	Solyc08g076790.3
6.30116	IP_OE_9_vs_In_OE_9_peak_1429	Solyc08g076860.3
3.95242	IP_OE_9_vs_In_OE_9_peak_1430	Solyc08g076930.1
5.77253	IP_OE_9_vs_In_OE_9_peak_1431	Solyc08g076960.1
3.53045	IP_OE_9_vs_In_OE_9_peak_1432	Solyc08g077060.3
4.12324	IP_OE_9_vs_In_OE_9_peak_1433	Solyc08g077070.3
5.5618	IP_OE_9_vs_In_OE_9_peak_1434	Solyc08g077110.3
3.80389	IP_OE_9_vs_In_OE_9_peak_1435	Solyc08g077150.3
5.91323	IP_OE_9_vs_In_OE_9_peak_1436	Solyc08g077220.3
4.28179	IP_OE_9_vs_In_OE_9_peak_1437	Solyc08g077370.3
4.56467	IP_OE_9_vs_In_OE_9_peak_1438	Solyc08g077440.3
4.87975	IP_OE_9_vs_In_OE_9_peak_1439	Solyc08g077530.3
4.87975	IP_OE_9_vs_In_OE_9_peak_1440	Solyc08g077640.2
5.45113	IP_OE_9_vs_In_OE_9_peak_1441	Solyc08g078020.1
6.17189	IP_OE_9_vs_In_OE_9_peak_1442	Solyc08g078090.1
3.36549	IP_OE_9_vs_In_OE_9_peak_1443	Solyc08g078250.3
7.09781	IP_OE_9_vs_In_OE_9_peak_1444	Solyc08g078650.3
4.18428	IP_OE_9_vs_In_OE_9_peak_1445	Solyc08g078670.2
4.53556	IP_OE_9_vs_In_OE_9_peak_1446	Solyc08g078780.2
5.76697	IP_OE_9_vs_In_OE_9_peak_1447	Solyc08g078790.1
4.56467	IP_OE_9_vs_In_OE_9_peak_1448	Solyc08g078810.1
4.87975	IP_OE_9_vs_In_OE_9_peak_1449	Solyc08g078980.1
4.27831	IP_OE_9_vs_In_OE_9_peak_1450	Solyc08g079180.3
4.43613	IP_OE_9_vs_In_OE_9_peak_1451	Solyc08g079470.3
6.21059	IP_OE_9_vs_In_OE_9_peak_1452	Solyc08g079560.1
3.62306	IP_OE_9_vs_In_OE_9_peak_1453	Solyc08g079650.3
3.54891	IP_OE_9_vs_In_OE_9_peak_1454	Solyc08g079670.3
5.76697	IP_OE_9_vs_In_OE_9_peak_1455	Solyc08g079690.3

4.53556	IP_OE_9_vs_In_OE_9_peak_1456	Solyc08g079740.3
4.94053	IP_OE_9_vs_In_OE_9_peak_1457	Solyc08g079760.3
4.87975	IP_OE_9_vs_In_OE_9_peak_1458	Solyc08g080050.3
3.54891	IP_OE_9_vs_In_OE_9_peak_1459	Solyc08g080100.3
3.53045	IP_OE_9_vs_In_OE_9_peak_1460	Solyc08g080120.3
5.76697	IP_OE_9_vs_In_OE_9_peak_1461	Solyc08g080130.3
3.4235	IP_OE_9_vs_In_OE_9_peak_1462	Solyc08g080150.1
4.43613	IP_OE_9_vs_In_OE_9_peak_1463	Solyc08g080200.3
4.87975	IP_OE_9_vs_In_OE_9_peak_1464	Solyc08g080540.3
5.32336	IP_OE_9_vs_In_OE_9_peak_1465	Solyc08g080750.3
4.18428	IP_OE_9_vs_In_OE_9_peak_1466	Solyc08g080960.3
4.43613	IP_OE_9_vs_In_OE_9_peak_1467	Solyc08g080990.3
4.18428	IP_OE_9_vs_In_OE_9_peak_1468	Solyc08g081020.1
3.54891	IP_OE_9_vs_In_OE_9_peak_1469	Solyc08g081190.3
5.66638	IP_OE_9_vs_In_OE_9_peak_1470	Solyc08g081210.3
4.43613	IP_OE_9_vs_In_OE_9_peak_1471	Solyc08g081220.1
4.43613	IP_OE_9_vs_In_OE_9_peak_1472	Solyc08g081230.1
3.99252	IP_OE_9_vs_In_OE_9_peak_1473	Solyc08g081310.3
5.13397	IP_OE_9_vs_In_OE_9_peak_1474	Solyc08g081390.3
4.75884	IP_OE_9_vs_In_OE_9_peak_1475	Solyc08g081385.1
5.88108	IP_OE_9_vs_In_OE_9_peak_1476	Solyc08g081480.3
4.43613	IP_OE_9_vs_In_OE_9_peak_1477	Solyc08g081493.1
3.99252	IP_OE_9_vs_In_OE_9_peak_1478	Solyc08g081610.3
4.87975	IP_OE_9_vs_In_OE_9_peak_1479	Solyc08g081690.3
4.68022	IP_OE_9_vs_In_OE_9_peak_1480	Solyc08g081700.1
4.18428	IP_OE_9_vs_In_OE_9_peak_1481	Solyc08g082110.3
4.23654	IP_OE_9_vs_In_OE_9_peak_1482	Solyc08g082210.3
6.10226	IP_OE_9_vs_In_OE_9_peak_1483	Solyc08g082370.1
5.32336	IP_OE_9_vs_In_OE_9_peak_1484	Solyc08g082460.3
6.21059	IP_OE_9_vs_In_OE_9_peak_1485	Solyc08g082590.3
2.99135	IP_OE_9_vs_In_OE_9_peak_1486	Solyc08g082610.3
4.43613	IP_OE_9_vs_In_OE_9_peak_1487	Solyc08g082670.3
6.21059	IP_OE_9_vs_In_OE_9_peak_1488	Solyc08g082990.3
4.43613	IP_OE_9_vs_In_OE_9_peak_1489	Solyc08g083060.3
4.31816	IP_OE_9_vs_In_OE_9_peak_1490	Solyc08g083130.3
5.32336	IP_OE_9_vs_In_OE_9_peak_1491	Solyc08g083140.3
3.53045	IP_OE_9_vs_In_OE_9_peak_1492	Solyc08g083400.3
4.87975	IP_OE_9_vs_In_OE_9_peak_1493	Solyc09g004991.1
6.21059	IP_OE_9_vs_In_OE_9_peak_1494	Solyc09g005080.1
3.73394	IP_OE_9_vs_In_OE_9_peak_1495	Solyc09g005550.3
5.32336	IP_OE_9_vs_In_OE_9_peak_1496	Solyc09g005570.3
6.57523	IP_OE_9_vs_In_OE_9_peak_1497	Solyc09g005840.1
4.86461	IP_OE_9_vs_In_OE_9_peak_1498	Solyc09g005860.3
3.53045	IP_OE_9_vs_In_OE_9_peak_1499	Solyc09g007560.3
4.43613	IP_OE_9_vs_In_OE_9_peak_1500	Solyc09g007770.2
5.32544	IP_OE_9_vs_In_OE_9_peak_1501	Solyc09g007790.1
4.94506	IP_OE_9_vs_In_OE_9_peak_1502	Solyc09g007900.3

4.69747	IP_OE_9_vs_In_OE_9_peak_1503	Solyc09g008060.3
4.87975	IP_OE_9_vs_In_OE_9_peak_1504	Solyc09g008240.3
5.32336	IP_OE_9_vs_In_OE_9_peak_1505	Solyc09g008270.3
4.4085	IP_OE_9_vs_In_OE_9_peak_1506	Solyc09g008610.3
6.59718	IP_OE_9_vs_In_OE_9_peak_1507	Solyc09g008830.3
5.98963	IP_OE_9_vs_In_OE_9_peak_1508	Solyc09g009080.3
5.36021	IP_OE_9_vs_In_OE_9_peak_1509	Solyc09g009100.3
4.87975	IP_OE_9_vs_In_OE_9_peak_1510	Solyc09g009130.3
4.05384	IP_OE_9_vs_In_OE_9_peak_1511	Solyc09g009150.1
5.32336	IP_OE_9_vs_In_OE_9_peak_1512	Solyc09g009420.1
5.24736	IP_OE_9_vs_In_OE_9_peak_1513	Solyc09g009520.3
3.19458	IP_OE_9_vs_In_OE_9_peak_1514	Solyc09g009980.2
5.32544	IP_OE_9_vs_In_OE_9_peak_1515	Solyc09g010000.3
4.62211	IP_OE_9_vs_In_OE_9_peak_1516	Solyc09g010220.3
4.87975	IP_OE_9_vs_In_OE_9_peak_1517	Solyc09g010230.2
5.7764	IP_OE_9_vs_In_OE_9_peak_1518	Solyc09g010640.2
3.485	IP_OE_9_vs_In_OE_9_peak_1519	Solyc09g010800.4
4.94789	IP_OE_9_vs_In_OE_9_peak_1520	Solyc09g010950.3
4.33431	IP_OE_9_vs_In_OE_9_peak_1521	Solyc09g010960.3
3.54891	IP_OE_9_vs_In_OE_9_peak_1522	Solyc09g011270.3
5.26999	IP_OE_9_vs_In_OE_9_peak_1523	Solyc09g011310.3
6.21059	IP_OE_9_vs_In_OE_9_peak_1524	Solyc09g011400.1
6.21059	IP_OE_9_vs_In_OE_9_peak_1525	Solyc09g011715.1
2.1695	IP_OE_9_vs_In_OE_9_peak_1526	Solyc09g011720.3
5.32336	IP_OE_9_vs_In_OE_9_peak_1527	Solyc09g014250.3
6.21059	IP_OE_9_vs_In_OE_9_peak_1528	Solyc09g014380.3
3.99252	IP_OE_9_vs_In_OE_9_peak_1529	Solyc09g014520.3
5.32336	IP_OE_9_vs_In_OE_9_peak_1530	Solyc09g014900.3
7.09781	IP_OE_9_vs_In_OE_9_peak_1531	Solyc09g014910.3
3.99252	IP_OE_9_vs_In_OE_9_peak_1532	Solyc09g015600.2
4.35624	IP_OE_9_vs_In_OE_9_peak_1533	Solyc09g015690.1
4.87975	IP_OE_9_vs_In_OE_9_peak_1534	Solyc09g015700.3
4.28179	IP_OE_9_vs_In_OE_9_peak_1535	Solyc09g015770.3
3.99252	IP_OE_9_vs_In_OE_9_peak_1536	Solyc09g015880.3
5.32336	IP_OE_9_vs_In_OE_9_peak_1537	Solyc09g015900.1
5.70933	IP_OE_9_vs_In_OE_9_peak_1538	Solyc09g018160.3
5.48358	IP_OE_9_vs_In_OE_9_peak_1539	Solyc09g018170.3
5.32336	IP_OE_9_vs_In_OE_9_peak_1540	Solyc09g018220.2
4.87975	IP_OE_9_vs_In_OE_9_peak_1541	Solyc09g018250.2
5.46101	IP_OE_9_vs_In_OE_9_peak_1542	Solyc09g018490.3
1.85843	IP_OE_9_vs_In_OE_9_peak_1543	Solyc09g018720.3
2.55428	IP_OE_9_vs_In_OE_9_peak_1544	Solyc09g018850.3
3.65768	IP_OE_9_vs_In_OE_9_peak_1545	Solyc09g042260.3
4.87975	IP_OE_9_vs_In_OE_9_peak_1546	Solyc09g042600.1
3.54891	IP_OE_9_vs_In_OE_9_peak_1547	Solyc09g042722.1
3.71092	IP_OE_9_vs_In_OE_9_peak_1548	Solyc09g031840.1
4.87975	IP_OE_9_vs_In_OE_9_peak_1549	Solyc09g031820.1

3.54891	IP_OE_9_vs_In_OE_9_peak_1550	Solyc09g031522.1
4.45922	IP_OE_9_vs_In_OE_9_peak_1551	Solyc09g030420.3
3.40703	IP_OE_9_vs_In_OE_9_peak_1552	Solyc09g050020.2
2.70318	IP_OE_9_vs_In_OE_9_peak_1553	Solyc09g050040.1
3.54891	IP_OE_9_vs_In_OE_9_peak_1554	Solyc09g050050.1
3.83883	IP_OE_9_vs_In_OE_9_peak_1555	Solyc09g055200.1
4.30852	IP_OE_9_vs_In_OE_9_peak_1556	Solyc09g055260.3
3.8835	IP_OE_9_vs_In_OE_9_peak_1557	Solyc09g055920.3
4.21734	IP_OE_9_vs_In_OE_9_peak_1558	Solyc09g055950.1
4.4085	IP_OE_9_vs_In_OE_9_peak_1559	Solyc09g055960.1
4.43613	IP_OE_9_vs_In_OE_9_peak_1560	Solyc09g056360.3
4.53556	IP_OE_9_vs_In_OE_9_peak_1561	Solyc09g059170.2
6.6542	IP_OE_9_vs_In_OE_9_peak_1562	Solyc09g059510.3
4.77667	IP_OE_9_vs_In_OE_9_peak_1563	Solyc09g059750.1
5.55603	IP_OE_9_vs_In_OE_9_peak_1564	Solyc09g059880.1
4.18428	IP_OE_9_vs_In_OE_9_peak_1565	Solyc09g061840.3
5.32055	IP_OE_9_vs_In_OE_9_peak_1566	Solyc09g063015.1
4.87975	IP_OE_9_vs_In_OE_9_peak_1567	Solyc09g063070.3
5.32336	IP_OE_9_vs_In_OE_9_peak_1568	Solyc09g064410.1
4.43613	IP_OE_9_vs_In_OE_9_peak_1569	Solyc09g064580.2
6.21059	IP_OE_9_vs_In_OE_9_peak_1570	Solyc09g064820.1
4.94263	IP_OE_9_vs_In_OE_9_peak_1571	Solyc09g064840.3
7.54143	IP_OE_9_vs_In_OE_9_peak_1572	Solyc09g064860.3
3.54891	IP_OE_9_vs_In_OE_9_peak_1573	Solyc09g065150.1
3.93769	IP_OE_9_vs_In_OE_9_peak_1574	Solyc09g065710.1
4.5386	IP_OE_9_vs_In_OE_9_peak_1575	Solyc09g065790.1
5.66638	IP_OE_9_vs_In_OE_9_peak_1576	Solyc09g065820.3
4.43613	IP_OE_9_vs_In_OE_9_peak_1577	Solyc09g066010.3
4.87975	IP_OE_9_vs_In_OE_9_peak_1578	Solyc09g066495.1
4.61116	IP_OE_9_vs_In_OE_9_peak_1579	Solyc09g074050.3
6.21059	IP_OE_9_vs_In_OE_9_peak_1580	Solyc09g074500.2
3.99252	IP_OE_9_vs_In_OE_9_peak_1581	Solyc09g074560.3
5.13397	IP_OE_9_vs_In_OE_9_peak_1582	Solyc09g075010.3
5.89176	IP_OE_9_vs_In_OE_9_peak_1583	Solyc09g075110.1
3.64846	IP_OE_9_vs_In_OE_9_peak_1584	Solyc09g075445.1
5.32336	IP_OE_9_vs_In_OE_9_peak_1585	Solyc09g075460.3
3.8835	IP_OE_9_vs_In_OE_9_peak_1586	Solyc09g075830.3
3.71092	IP_OE_9_vs_In_OE_9_peak_1587	Solyc09g075970.3
5.32336	IP_OE_9_vs_In_OE_9_peak_1588	Solyc09g076000.3
5.5618	IP_OE_9_vs_In_OE_9_peak_1589	Solyc09g082220.1
3.95242	IP_OE_9_vs_In_OE_9_peak_1590	Solyc09g082490.3
7.09781	IP_OE_9_vs_In_OE_9_peak_1591	Solyc09g082500.3
3.84338	IP_OE_9_vs_In_OE_9_peak_1592	Solyc09g082505.1
4.43613	IP_OE_9_vs_In_OE_9_peak_1593	Solyc09g082630.3
5.32336	IP_OE_9_vs_In_OE_9_peak_1594	Solyc09g082780.3
4.42533	IP_OE_9_vs_In_OE_9_peak_1595	Solyc09g083000.3
3.99252	IP_OE_9_vs_In_OE_9_peak_1596	Solyc09g083050.3

4.62211	IP_OE_9_vs_In_OE_9_peak_1597	Solyc09g083200.3
4.84014	IP_OE_9_vs_In_OE_9_peak_1598	Solyc09g083210.3
3.99252	IP_OE_9_vs_In_OE_9_peak_1599	Solyc09g083280.3
3.62306	IP_OE_9_vs_In_OE_9_peak_1600	Solyc09g083390.2
3.99252	IP_OE_9_vs_In_OE_9_peak_1601	Solyc09g089650.1
4.23654	IP_OE_9_vs_In_OE_9_peak_1602	Solyc09g089670.3
5.13397	IP_OE_9_vs_In_OE_9_peak_1603	Solyc09g089890.1
4.56467	IP_OE_9_vs_In_OE_9_peak_1604	Solyc09g089910.1
4.43613	IP_OE_9_vs_In_OE_9_peak_1605	Solyc09g090070.1
5.36021	IP_OE_9_vs_In_OE_9_peak_1606	Solyc09g090080.1
3.99252	IP_OE_9_vs_In_OE_9_peak_1607	Solyc09g090140.3
3.99252	IP_OE_9_vs_In_OE_9_peak_1608	Solyc09g090170.1
5.47092	IP_OE_9_vs_In_OE_9_peak_1609	Solyc09g090200.3
5.32336	IP_OE_9_vs_In_OE_9_peak_1610	Solyc09g090270.3
3.99252	IP_OE_9_vs_In_OE_9_peak_1611	Solyc09g090470.3
5.32336	IP_OE_9_vs_In_OE_9_peak_1612	Solyc09g090680.3
3.39535	IP_OE_9_vs_In_OE_9_peak_1613	Solyc09g090730.2
4.27831	IP_OE_9_vs_In_OE_9_peak_1614	Solyc09g090960.3
4.23654	IP_OE_9_vs_In_OE_9_peak_1615	Solyc09g090980.3
4.87975	IP_OE_9_vs_In_OE_9_peak_1616	Solyc09g091090.2
7.98504	IP_OE_9_vs_In_OE_9_peak_1617	Solyc09g091250.3
4.70614	IP_OE_9_vs_In_OE_9_peak_1618	Solyc09g091370.3
4.43613	IP_OE_9_vs_In_OE_9_peak_1619	Solyc09g091410.1
4.43613	IP_OE_9_vs_In_OE_9_peak_1620	Solyc09g091810.1
5.76697	IP_OE_9_vs_In_OE_9_peak_1621	Solyc09g092270.3
4.43613	IP_OE_9_vs_In_OE_9_peak_1622	Solyc09g092550.3
3.8835	IP_OE_9_vs_In_OE_9_peak_1623	Solyc09g098070.3
3.13804	IP_OE_9_vs_In_OE_9_peak_1624	Solyc09g098290.3
3.39535	IP_OE_9_vs_In_OE_9_peak_1625	Solyc10g005000.3
4.43613	IP_OE_9_vs_In_OE_9_peak_1626	Solyc10g005100.3
4.42533	IP_OE_9_vs_In_OE_9_peak_1627	Solyc10g005195.1
3.99252	IP_OE_9_vs_In_OE_9_peak_1628	Solyc10g005360.3
5.70583	IP_OE_9_vs_In_OE_9_peak_1629	Solyc10g005510.3
5.70933	IP_OE_9_vs_In_OE_9_peak_1630	Solyc10g006140.3
3.54891	IP_OE_9_vs_In_OE_9_peak_1631	Solyc10g006250.3
3.6999	IP_OE_9_vs_In_OE_9_peak_1632	Solyc10g006910.2
3.99252	IP_OE_9_vs_In_OE_9_peak_1633	Solyc10g007560.2
4.86786	IP_OE_9_vs_In_OE_9_peak_1634	Solyc10g008260.2
3.94514	IP_OE_9_vs_In_OE_9_peak_1635	Solyc10g008270.3
5.32336	IP_OE_9_vs_In_OE_9_peak_1636	Solyc10g008520.3
4.35058	IP_OE_9_vs_In_OE_9_peak_1637	Solyc10g008660.2
4.5386	IP_OE_9_vs_In_OE_9_peak_1638	Solyc10g008680.2
4.28179	IP_OE_9_vs_In_OE_9_peak_1639	Solyc10g009100.2
3.99252	IP_OE_9_vs_In_OE_9_peak_1640	Solyc10g009110.1
5.1057	IP_OE_9_vs_In_OE_9_peak_1641	Solyc10g009120.2
4.87975	IP_OE_9_vs_In_OE_9_peak_1642	Solyc10g009190.1
3.99252	IP_OE_9_vs_In_OE_9_peak_1643	Solyc10g009270.3

4.87975	IP_OE_9_vs_In_OE_9_peak_1644	Solyc10g009360.3
4.43613	IP_OE_9_vs_In_OE_9_peak_1645	Solyc10g009550.3
4.87975	IP_OE_9_vs_In_OE_9_peak_1646	Solyc10g012230.1
5.70583	IP_OE_9_vs_In_OE_9_peak_1647	Solyc10g017810.2
4.87975	IP_OE_9_vs_In_OE_9_peak_1648	Solyc10g017890.1
4.14049	IP_OE_9_vs_In_OE_9_peak_1649	Solyc10g017920.1
4.53556	IP_OE_9_vs_In_OE_9_peak_1650	Solyc10g017950.2
5.76697	IP_OE_9_vs_In_OE_9_peak_1651	Solyc10g018340.1
4.26257	IP_OE_9_vs_In_OE_9_peak_1652	Solyc10g018520.1
4.12324	IP_OE_9_vs_In_OE_9_peak_1653	Solyc10g018600.2
4.77667	IP_OE_9_vs_In_OE_9_peak_1654	Solyc10g018780.2
4.54656	IP_OE_9_vs_In_OE_9_peak_1655	Solyc10g018907.1
4.43613	IP_OE_9_vs_In_OE_9_peak_1656	Solyc10g039230.1
5.87044	IP_OE_9_vs_In_OE_9_peak_1657	Solyc10g039295.1
3.62306	IP_OE_9_vs_In_OE_9_peak_1658	Solyc10g061990.2
3.71092	IP_OE_9_vs_In_OE_9_peak_1659	Solyc10g044490.2
5.36021	IP_OE_9_vs_In_OE_9_peak_1660	Solyc10g045240.2
5.31039	IP_OE_9_vs_In_OE_9_peak_1661	Solyc10g045380.2
6.6542	IP_OE_9_vs_In_OE_9_peak_1662	Solyc10g046790.1
5.25025	IP_OE_9_vs_In_OE_9_peak_1663	Solyc10g047520.1
3.7807	IP_OE_9_vs_In_OE_9_peak_1664	Solyc10g047680.2
4.43613	IP_OE_9_vs_In_OE_9_peak_1665	Solyc10g048060.1
4.43613	IP_OE_9_vs_In_OE_9_peak_1666	Solyc10g048065.1
4.62211	IP_OE_9_vs_In_OE_9_peak_1667	Solyc10g049640.2
4.43613	IP_OE_9_vs_In_OE_9_peak_1668	Solyc10g049930.1
5.58241	IP_OE_9_vs_In_OE_9_peak_1669	Solyc10g050920.1
3.99252	IP_OE_9_vs_In_OE_9_peak_1670	Solyc10g050970.1
4.12324	IP_OE_9_vs_In_OE_9_peak_1671	Solyc10g051080.1
6.70786	IP_OE_9_vs_In_OE_9_peak_1672	Solyc10g051225.1
4.24695	IP_OE_9_vs_In_OE_9_peak_1673	Solyc10g054030.2
5.32336	IP_OE_9_vs_In_OE_9_peak_1674	Solyc10g054170.2
4.87975	IP_OE_9_vs_In_OE_9_peak_1675	Solyc10g054440.2
3.99252	IP_OE_9_vs_In_OE_9_peak_1676	Solyc10g054540.1
4.87975	IP_OE_9_vs_In_OE_9_peak_1677	Solyc10g054570.2
4.63003	IP_OE_9_vs_In_OE_9_peak_1678	Solyc10g054910.1
4.87313	IP_OE_9_vs_In_OE_9_peak_1679	Solyc10g055680.1
7.09781	IP_OE_9_vs_In_OE_9_peak_1680	Solyc10g055750.1
6.08622	IP_OE_9_vs_In_OE_9_peak_1681	Solyc10g074730.3
4.11867	IP_OE_9_vs_In_OE_9_peak_1682	Solyc10g074935.1
4.43613	IP_OE_9_vs_In_OE_9_peak_1683	Solyc10g075070.2
4.23654	IP_OE_9_vs_In_OE_9_peak_1684	Solyc10g076290.2
5.23051	IP_OE_9_vs_In_OE_9_peak_1685	Solyc10g077040.2
6.85795	IP_OE_9_vs_In_OE_9_peak_1686	Solyc10g077130.1
4.94263	IP_OE_9_vs_In_OE_9_peak_1687	Solyc10g078340.2
6.6542	IP_OE_9_vs_In_OE_9_peak_1688	Solyc10g078610.1
5.36021	IP_OE_9_vs_In_OE_9_peak_1689	Solyc10g078900.2
7.12926	IP_OE_9_vs_In_OE_9_peak_1690	Solyc10g078920.2

4.9788	IP_OE_9_vs_In_OE_9_peak_1691	Solyc10g079060.2
4.56467	IP_OE_9_vs_In_OE_9_peak_1692	Solyc10g079350.2
4.86461	IP_OE_9_vs_In_OE_9_peak_1693	Solyc10g079620.2
5.54134	IP_OE_9_vs_In_OE_9_peak_1694	Solyc10g079640.2
4.43613	IP_OE_9_vs_In_OE_9_peak_1695	Solyc10g079755.1
4.87975	IP_OE_9_vs_In_OE_9_peak_1696	Solyc10g079790.1
6.18486	IP_OE_9_vs_In_OE_9_peak_1697	Solyc10g080050.2
3.80389	IP_OE_9_vs_In_OE_9_peak_1698	Solyc10g080360.1
4.58959	IP_OE_9_vs_In_OE_9_peak_1699	Solyc10g080450.2
4.56467	IP_OE_9_vs_In_OE_9_peak_1700	Solyc10g080470.1
4.15559	IP_OE_9_vs_In_OE_9_peak_1701	Solyc10g080590.2
5.32336	IP_OE_9_vs_In_OE_9_peak_1702	Solyc10g080600.2
4.5386	IP_OE_9_vs_In_OE_9_peak_1703	Solyc10g080900.2
4.43613	IP_OE_9_vs_In_OE_9_peak_1704	Solyc10g080920.2
4.87975	IP_OE_9_vs_In_OE_9_peak_1705	Solyc10g080940.2
5.14405	IP_OE_9_vs_In_OE_9_peak_1706	Solyc10g081050.1
4.53556	IP_OE_9_vs_In_OE_9_peak_1707	Solyc10g081170.2
4.94506	IP_OE_9_vs_In_OE_9_peak_1708	Solyc10g081190.2
4.82183	IP_OE_9_vs_In_OE_9_peak_1709	Solyc10g081320.1
5.18179	IP_OE_9_vs_In_OE_9_peak_1710	Solyc10g081460.2
4.87975	IP_OE_9_vs_In_OE_9_peak_1711	Solyc10g081500.1
4.79463	IP_OE_9_vs_In_OE_9_peak_1712	Solyc10g081900.2
4.126	IP_OE_9_vs_In_OE_9_peak_1713	Solyc10g083200.2
3.45453	IP_OE_9_vs_In_OE_9_peak_1714	Solyc10g083210.2
5.76697	IP_OE_9_vs_In_OE_9_peak_1715	Solyc10g083220.2
3.99252	IP_OE_9_vs_In_OE_9_peak_1716	Solyc10g083290.4
4.87975	IP_OE_9_vs_In_OE_9_peak_1717	Solyc10g083300.2
4.77667	IP_OE_9_vs_In_OE_9_peak_1718	Solyc10g083310.2
4.43613	IP_OE_9_vs_In_OE_9_peak_1719	Solyc10g083410.1
7.09781	IP_OE_9_vs_In_OE_9_peak_1720	Solyc10g083850.2
4.79463	IP_OE_9_vs_In_OE_9_peak_1721	Solyc10g083980.1
3.99252	IP_OE_9_vs_In_OE_9_peak_1722	Solyc10g084023.1
4.76774	IP_OE_9_vs_In_OE_9_peak_1723	Solyc10g084250.1
4.87975	IP_OE_9_vs_In_OE_9_peak_1724	Solyc10g084370.2
4.43613	IP_OE_9_vs_In_OE_9_peak_1725	Solyc10g084600.2
3.99252	IP_OE_9_vs_In_OE_9_peak_1726	Solyc10g084960.2
4.87975	IP_OE_9_vs_In_OE_9_peak_1727	Solyc10g084970.2
6.847	IP_OE_9_vs_In_OE_9_peak_1728	Solyc10g085010.2
6.21059	IP_OE_9_vs_In_OE_9_peak_1729	Solyc10g085020.2
4.87975	IP_OE_9_vs_In_OE_9_peak_1730	Solyc10g085460.2
3.54891	IP_OE_9_vs_In_OE_9_peak_1731	Solyc10g085480.2
3.29369	IP_OE_9_vs_In_OE_9_peak_1732	Solyc10g085800.2
4.87975	IP_OE_9_vs_In_OE_9_peak_1733	Solyc10g086000.2
4.87975	IP_OE_9_vs_In_OE_9_peak_1734	Solyc10g086280.2
2.31293	IP_OE_9_vs_In_OE_9_peak_1735	Solyc10g086330.2
2.8146	IP_OE_9_vs_In_OE_9_peak_1736	Solyc10g086380.1
2.07687	IP_OE_9_vs_In_OE_9_peak_1737	Solyc10g086520.2

3.99252	IP_OE_9_vs_In_OE_9_peak_1738	Solyc10g086730.2
4.87975	IP_OE_9_vs_In_OE_9_peak_1739	Solyc11g005700.1
4.10065	IP_OE_9_vs_In_OE_9_peak_1740	Solyc11g005710.2
6.21059	IP_OE_9_vs_In_OE_9_peak_1741	Solyc11g005860.2
4.28179	IP_OE_9_vs_In_OE_9_peak_1742	Solyc11g006710.2
4.43613	IP_OE_9_vs_In_OE_9_peak_1743	Solyc11g006900.1
3.71092	IP_OE_9_vs_In_OE_9_peak_1744	Solyc11g007430.2
3.99252	IP_OE_9_vs_In_OE_9_peak_1745	Solyc11g007660.1
4.94506	IP_OE_9_vs_In_OE_9_peak_1746	Solyc11g008140.2
4.37841	IP_OE_9_vs_In_OE_9_peak_1747	Solyc11g008200.1
3.99252	IP_OE_9_vs_In_OE_9_peak_1748	Solyc11g008670.2
2.8909	IP_OE_9_vs_In_OE_9_peak_1749	Solyc11g008780.2
4.83903	IP_OE_9_vs_In_OE_9_peak_1750	Solyc11g009050.2
5.32336	IP_OE_9_vs_In_OE_9_peak_1751	Solyc11g010120.2
3.49307	IP_OE_9_vs_In_OE_9_peak_1752	Solyc11g010260.2
4.86461	IP_OE_9_vs_In_OE_9_peak_1753	Solyc11g010280.1
4.72357	IP_OE_9_vs_In_OE_9_peak_1754	Solyc11g010270.2
2.09475	IP_OE_9_vs_In_OE_9_peak_1755	Solyc11g010330.2
2.63638	IP_OE_9_vs_In_OE_9_peak_1756	Solyc11g010430.2
3.06665	IP_OE_9_vs_In_OE_9_peak_1757	Solyc11g010500.1
4.43613	IP_OE_9_vs_In_OE_9_peak_1758	Solyc11g011040.2
8.42865	IP_OE_9_vs_In_OE_9_peak_1759	Solyc11g011050.2
3.99252	IP_OE_9_vs_In_OE_9_peak_1760	Solyc11g011240.1
3.28329	IP_OE_9_vs_In_OE_9_peak_1761	Solyc11g011260.1
4.43613	IP_OE_9_vs_In_OE_9_peak_1762	Solyc11g011380.2
3.80389	IP_OE_9_vs_In_OE_9_peak_1763	Solyc11g011502.1
3.8835	IP_OE_9_vs_In_OE_9_peak_1764	Solyc11g011780.2
4.43613	IP_OE_9_vs_In_OE_9_peak_1765	Solyc11g012250.1
5.32336	IP_OE_9_vs_In_OE_9_peak_1766	Solyc11g012320.2
16.36128	IP_OE_9_vs_In_OE_9_peak_1767	Solyc11g012360.2
5.76697	IP_OE_9_vs_In_OE_9_peak_1768	Solyc11g012455.1
4.84935	IP_OE_9_vs_In_OE_9_peak_1769	Solyc11g012670.1
5.88108	IP_OE_9_vs_In_OE_9_peak_1770	Solyc11g012680.2
2.54832	IP_OE_9_vs_In_OE_9_peak_1771	Solyc11g012700.2
2.58574	IP_OE_9_vs_In_OE_9_peak_1772	Solyc11g013010.2
2.40583	IP_OE_9_vs_In_OE_9_peak_1773	Solyc11g013170.2
4.87975	IP_OE_9_vs_In_OE_9_peak_1774	Solyc11g013190.2
3.71092	IP_OE_9_vs_In_OE_9_peak_1775	Solyc11g013250.1
4.12324	IP_OE_9_vs_In_OE_9_peak_1776	Solyc11g013450.2
3.90818	IP_OE_9_vs_In_OE_9_peak_1777	Solyc11g013480.2
3.56007	IP_OE_9_vs_In_OE_9_peak_1778	Solyc11g017000.2
2.94569	IP_OE_9_vs_In_OE_9_peak_1779	Solyc11g017280.2
5.45113	IP_OE_9_vs_In_OE_9_peak_1780	Solyc11g017450.2
4.94789	IP_OE_9_vs_In_OE_9_peak_1781	Solyc11g018560.2
4.27831	IP_OE_9_vs_In_OE_9_peak_1782	Solyc11g020217.1
5.66638	IP_OE_9_vs_In_OE_9_peak_1783	Solyc11g020600.1
3.48701	IP_OE_9_vs_In_OE_9_peak_1784	Solyc11g020670.1

5.26018	IP_OE_9_vs_In_OE_9_peak_1785	Solyc11g020930.1
4.87975	IP_OE_9_vs_In_OE_9_peak_1786	Solyc11g021140.1
5.32336	IP_OE_9_vs_In_OE_9_peak_1787	Solyc11g021160.1
3.8835	IP_OE_9_vs_In_OE_9_peak_1788	Solyc11g021210.1
4.47488	IP_OE_9_vs_In_OE_9_peak_1789	Solyc11g021270.1
5.76697	IP_OE_9_vs_In_OE_9_peak_1790	Solyc11g021310.1
4.53556	IP_OE_9_vs_In_OE_9_peak_1791	Solyc11g022610.1
5.36021	IP_OE_9_vs_In_OE_9_peak_1792	Solyc11g051170.2
4.35876	IP_OE_9_vs_In_OE_9_peak_1793	Solyc11g051200.1
4.53556	IP_OE_9_vs_In_OE_9_peak_1794	Solyc11g027630.1
4.87975	IP_OE_9_vs_In_OE_9_peak_1795	Solyc11g027645.1
4.781	IP_OE_9_vs_In_OE_9_peak_1796	Solyc11g027670.1
3.71092	IP_OE_9_vs_In_OE_9_peak_1797	Solyc11g027710.1
4.18428	IP_OE_9_vs_In_OE_9_peak_1798	Solyc11g027730.1
3.8835	IP_OE_9_vs_In_OE_9_peak_1799	Solyc11g027770.1
4.87975	IP_OE_9_vs_In_OE_9_peak_1800	Solyc11g030910.1
4.18428	IP_OE_9_vs_In_OE_9_peak_1801	Solyc11g045260.1
5.76697	IP_OE_9_vs_In_OE_9_peak_1802	Solyc11g045150.1
3.54891	IP_OE_9_vs_In_OE_9_peak_1803	Solyc11g044610.1
3.4235	IP_OE_9_vs_In_OE_9_peak_1804	Solyc11g044360.2
4.43613	IP_OE_9_vs_In_OE_9_peak_1805	Solyc11g044320.2
4.12324	IP_OE_9_vs_In_OE_9_peak_1806	Solyc11g042650.1
4.05384	IP_OE_9_vs_In_OE_9_peak_1807	Solyc11g040110.2
7.06703	IP_OE_9_vs_In_OE_9_peak_1808	Solyc11g039990.1
3.99252	IP_OE_9_vs_In_OE_9_peak_1809	Solyc11g039860.2
3.99252	IP_OE_9_vs_In_OE_9_peak_1810	Solyc11g039420.2
4.86461	IP_OE_9_vs_In_OE_9_peak_1811	Solyc11g039380.1
5.88108	IP_OE_9_vs_In_OE_9_peak_1812	Solyc11g039370.2
4.70021	IP_OE_9_vs_In_OE_9_peak_1813	Solyc11g039360.1
4.23654	IP_OE_9_vs_In_OE_9_peak_1814	Solyc11g039350.1
4.43613	IP_OE_9_vs_In_OE_9_peak_1815	Solyc11g038350.1
5.3638	IP_OE_9_vs_In_OE_9_peak_1816	Solyc11g038340.1
6.21059	IP_OE_9_vs_In_OE_9_peak_1817	Solyc11g056270.2
4.43613	IP_OE_9_vs_In_OE_9_peak_1818	Solyc11g056280.1
4.43379	IP_OE_9_vs_In_OE_9_peak_1819	Solyc11g056290.2
7.54143	IP_OE_9_vs_In_OE_9_peak_1820	Solyc11g056310.1
4.53556	IP_OE_9_vs_In_OE_9_peak_1821	Solyc11g056330.1
4.30213	IP_OE_9_vs_In_OE_9_peak_1822	Solyc11g056340.1
4.43613	IP_OE_9_vs_In_OE_9_peak_1823	Solyc11g056350.1
6.6542	IP_OE_9_vs_In_OE_9_peak_1824	Solyc11g056370.1
4.43613	IP_OE_9_vs_In_OE_9_peak_1825	Solyc11g056380.1
5.36021	IP_OE_9_vs_In_OE_9_peak_1826	Solyc11g056440.1
3.8576	IP_OE_9_vs_In_OE_9_peak_1827	Solyc11g056460.1
5.36021	IP_OE_9_vs_In_OE_9_peak_1828	Solyc11g056470.2
4.43613	IP_OE_9_vs_In_OE_9_peak_1829	Solyc11g056510.1
5.70583	IP_OE_9_vs_In_OE_9_peak_1830	Solyc11g056530.1
4.87975	IP_OE_9_vs_In_OE_9_peak_1831	Solyc11g061980.1

2.75549	IP_OE_9_vs_In_OE_9_peak_1832	Solyc11g062030.1
4.43613	IP_OE_9_vs_In_OE_9_peak_1833	Solyc11g062440.2
5.66638	IP_OE_9_vs_In_OE_9_peak_1834	Solyc11g063640.1
5.77253	IP_OE_9_vs_In_OE_9_peak_1835	Solyc11g065000.2
5.32336	IP_OE_9_vs_In_OE_9_peak_1836	Solyc11g065070.2
4.87975	IP_OE_9_vs_In_OE_9_peak_1837	Solyc11g065530.1
1.76181	IP_OE_9_vs_In_OE_9_peak_1838	Solyc11g065643.1
6.21059	IP_OE_9_vs_In_OE_9_peak_1839	Solyc11g065830.2
3.8835	IP_OE_9_vs_In_OE_9_peak_1840	Solyc11g066020.2
5.76697	IP_OE_9_vs_In_OE_9_peak_1841	Solyc11g066060.2
3.8835	IP_OE_9_vs_In_OE_9_peak_1842	Solyc11g066330.1
6.37466	IP_OE_9_vs_In_OE_9_peak_1843	Solyc11g066580.2
4.70614	IP_OE_9_vs_In_OE_9_peak_1844	Solyc11g066630.2
3.66674	IP_OE_9_vs_In_OE_9_peak_1845	Solyc11g066810.2
4.87975	IP_OE_9_vs_In_OE_9_peak_1846	Solyc11g066950.2
4.87975	IP_OE_9_vs_In_OE_9_peak_1847	Solyc11g067055.1
5.1364	IP_OE_9_vs_In_OE_9_peak_1848	Solyc11g067080.2
3.99252	IP_OE_9_vs_In_OE_9_peak_1849	Solyc11g068390.1
4.66144	IP_OE_9_vs_In_OE_9_peak_1850	Solyc11g068950.2
5.76697	IP_OE_9_vs_In_OE_9_peak_1851	Solyc11g068960.2
6.59718	IP_OE_9_vs_In_OE_9_peak_1852	Solyc11g069020.2
6.21059	IP_OE_9_vs_In_OE_9_peak_1853	Solyc11g069070.2
3.4235	IP_OE_9_vs_In_OE_9_peak_1854	Solyc11g069090.2
4.18428	IP_OE_9_vs_In_OE_9_peak_1855	Solyc11g069150.2
3.96765	IP_OE_9_vs_In_OE_9_peak_1856	Solyc11g069390.1
4.94263	IP_OE_9_vs_In_OE_9_peak_1857	Solyc11g069600.2
4.87717	IP_OE_9_vs_In_OE_9_peak_1858	Solyc11g069660.2
3.3106	IP_OE_9_vs_In_OE_9_peak_1859	Solyc11g069730.1
4.56467	IP_OE_9_vs_In_OE_9_peak_1860	Solyc11g069750.2
2.36278	IP_OE_9_vs_In_OE_9_peak_1861	Solyc11g070040.2
3.03853	IP_OE_9_vs_In_OE_9_peak_1862	Solyc11g072440.2
3.57671	IP_OE_9_vs_In_OE_9_peak_1863	Solyc11g072470.2
5.76697	IP_OE_9_vs_In_OE_9_peak_1864	Solyc11g072480.2
1.92866	IP_OE_9_vs_In_OE_9_peak_1865	Solyc11g072490.2
5.23051	IP_OE_9_vs_In_OE_9_peak_1866	Solyc11g072600.2
3.54891	IP_OE_9_vs_In_OE_9_peak_1867	Solyc11g072615.1
4.94506	IP_OE_9_vs_In_OE_9_peak_1868	Solyc11g072720.2
4.86461	IP_OE_9_vs_In_OE_9_peak_1869	Solyc11g072900.1
5.32336	IP_OE_9_vs_In_OE_9_peak_1870	Solyc12g005000.1
3.54891	IP_OE_9_vs_In_OE_9_peak_1871	Solyc12g005150.2
2.74585	IP_OE_9_vs_In_OE_9_peak_1872	Solyc12g005640.2
2.69408	IP_OE_9_vs_In_OE_9_peak_1873	Solyc12g005660.2
5.2902	IP_OE_9_vs_In_OE_9_peak_1874	Solyc12g005750.1
5.02816	IP_OE_9_vs_In_OE_9_peak_1875	Solyc12g005760.2
3.26681	IP_OE_9_vs_In_OE_9_peak_1876	Solyc12g005800.2
4.32136	IP_OE_9_vs_In_OE_9_peak_1877	Solyc12g005960.2
4.87975	IP_OE_9_vs_In_OE_9_peak_1878	Solyc12g006470.2

2.72986	IP_OE_9_vs_In_OE_9_peak_1879	Solyc12g006850.2
3.02177	IP_OE_9_vs_In_OE_9_peak_1880	Solyc12g006860.2
2.93343	IP_OE_9_vs_In_OE_9_peak_1881	Solyc12g007205.1
3.92946	IP_OE_9_vs_In_OE_9_peak_1882	Solyc12g007210.2
3.0366	IP_OE_9_vs_In_OE_9_peak_1883	Solyc12g008460.2
2.43392	IP_OE_9_vs_In_OE_9_peak_1884	Solyc12g008520.2
3.10396	IP_OE_9_vs_In_OE_9_peak_1885	Solyc12g008960.2
2.07	IP_OE_9_vs_In_OE_9_peak_1886	Solyc12g009520.2
2.78676	IP_OE_9_vs_In_OE_9_peak_1887	Solyc12g009790.2
2.54131	IP_OE_9_vs_In_OE_9_peak_1888	Solyc12g010130.1
2.78426	IP_OE_9_vs_In_OE_9_peak_1889	Solyc12g010540.1
2.6493	IP_OE_9_vs_In_OE_9_peak_1890	Solyc12g010720.1
2.05616	IP_OE_9_vs_In_OE_9_peak_1891	Solyc12g010730.2
2.18145	IP_OE_9_vs_In_OE_9_peak_1892	Solyc12g011100.2
3.4235	IP_OE_9_vs_In_OE_9_peak_1893	Solyc12g011320.2
3.2823	IP_OE_9_vs_In_OE_9_peak_1894	Solyc12g011460.1
4.23654	IP_OE_9_vs_In_OE_9_peak_1895	Solyc12g013840.2
6.21059	IP_OE_9_vs_In_OE_9_peak_1896	Solyc12g013895.1
6.70786	IP_OE_9_vs_In_OE_9_peak_1897	Solyc12g014040.1
5.76697	IP_OE_9_vs_In_OE_9_peak_1898	Solyc12g014417.1
6.60019	IP_OE_9_vs_In_OE_9_peak_1899	Solyc12g014420.2
4.43613	IP_OE_9_vs_In_OE_9_peak_1900	Solyc12g014490.2
4.87975	IP_OE_9_vs_In_OE_9_peak_1901	Solyc12g015700.2
5.59277	IP_OE_9_vs_In_OE_9_peak_1902	Solyc12g016217.1
6.21059	IP_OE_9_vs_In_OE_9_peak_1903	Solyc12g019480.2
3.91191	IP_OE_9_vs_In_OE_9_peak_1904	Solyc12g019885.1
3.4235	IP_OE_9_vs_In_OE_9_peak_1905	Solyc12g077590.2
4.87975	IP_OE_9_vs_In_OE_9_peak_1906	Solyc12g026400.2
3.63584	IP_OE_9_vs_In_OE_9_peak_1907	Solyc12g026470.2
5.32336	IP_OE_9_vs_In_OE_9_peak_1908	Solyc12g027820.1
3.99252	IP_OE_9_vs_In_OE_9_peak_1909	Solyc12g027850.2
4.43613	IP_OE_9_vs_In_OE_9_peak_1910	Solyc12g070080.2
4.87975	IP_OE_9_vs_In_OE_9_peak_1911	Solyc12g062840.1
3.58216	IP_OE_9_vs_In_OE_9_peak_1912	Solyc12g035620.2
6.6542	IP_OE_9_vs_In_OE_9_peak_1913	Solyc12g035650.2
4.86461	IP_OE_9_vs_In_OE_9_peak_1914	Solyc12g035675.1
4.87975	IP_OE_9_vs_In_OE_9_peak_1915	Solyc12g035826.1
5.62408	IP_OE_9_vs_In_OE_9_peak_1916	Solyc12g035875.1
4.12324	IP_OE_9_vs_In_OE_9_peak_1917	Solyc12g036165.1
3.8835	IP_OE_9_vs_In_OE_9_peak_1918	Solyc12g038540.2
6.81983	IP_OE_9_vs_In_OE_9_peak_1919	Solyc12g040860.2
3.54891	IP_OE_9_vs_In_OE_9_peak_1920	Solyc12g041880.2
5.32336	IP_OE_9_vs_In_OE_9_peak_1921	Solyc12g042077.1
6.08622	IP_OE_9_vs_In_OE_9_peak_1922	Solyc12g042230.2
5.32336	IP_OE_9_vs_In_OE_9_peak_1923	Solyc12g042460.2
3.53352	IP_OE_9_vs_In_OE_9_peak_1924	Solyc12g042480.2
4.43613	IP_OE_9_vs_In_OE_9_peak_1925	Solyc12g042730.1

4.70614	IP_OE_9_vs_In_OE_9_peak_1926	Solyc12g042760.1
5.32544	IP_OE_9_vs_In_OE_9_peak_1927	Solyc12g044600.3
4.46704	IP_OE_9_vs_In_OE_9_peak_1928	Solyc12g044670.1
4.53556	IP_OE_9_vs_In_OE_9_peak_1929	Solyc12g044750.2
4.76774	IP_OE_9_vs_In_OE_9_peak_1930	Solyc12g044800.2
4.05384	IP_OE_9_vs_In_OE_9_peak_1931	Solyc12g044940.2
3.79445	IP_OE_9_vs_In_OE_9_peak_1932	Solyc12g045030.2
4.87975	IP_OE_9_vs_In_OE_9_peak_1933	Solyc12g049030.1
4.43613	IP_OE_9_vs_In_OE_9_peak_1934	Solyc12g049190.2
5.32336	IP_OE_9_vs_In_OE_9_peak_1935	Solyc12g049500.2
4.56467	IP_OE_9_vs_In_OE_9_peak_1936	Solyc12g049560.2
4.12324	IP_OE_9_vs_In_OE_9_peak_1937	Solyc12g055710.1
4.87975	IP_OE_9_vs_In_OE_9_peak_1938	Solyc12g055840.2
3.8835	IP_OE_9_vs_In_OE_9_peak_1939	Solyc12g055990.2
3.54891	IP_OE_9_vs_In_OE_9_peak_1940	Solyc12g056140.1
5.30432	IP_OE_9_vs_In_OE_9_peak_1941	Solyc12g056350.2
6.2784	IP_OE_9_vs_In_OE_9_peak_1942	Solyc12g056370.1
5.76697	IP_OE_9_vs_In_OE_9_peak_1943	Solyc12g056510.2
3.80389	IP_OE_9_vs_In_OE_9_peak_1944	Solyc12g056520.2
5.13397	IP_OE_9_vs_In_OE_9_peak_1945	Solyc12g056580.2
5.32336	IP_OE_9_vs_In_OE_9_peak_1946	Solyc12g056620.2
3.35508	IP_OE_9_vs_In_OE_9_peak_1947	Solyc12g056625.1
3.70402	IP_OE_9_vs_In_OE_9_peak_1948	Solyc12g056740.2
4.87975	IP_OE_9_vs_In_OE_9_peak_1949	Solyc12g056980.1
3.99252	IP_OE_9_vs_In_OE_9_peak_1950	Solyc12g056990.1
4.85859	IP_OE_9_vs_In_OE_9_peak_1951	Solyc12g088580.1
4.43613	IP_OE_9_vs_In_OE_9_peak_1952	Solyc12g088660.2
3.80389	IP_OE_9_vs_In_OE_9_peak_1953	Solyc12g088670.2
3.99252	IP_OE_9_vs_In_OE_9_peak_1954	Solyc12g088680.2
4.43613	IP_OE_9_vs_In_OE_9_peak_1955	Solyc12g088720.2
4.54656	IP_OE_9_vs_In_OE_9_peak_1956	Solyc12g088760.1
6.21059	IP_OE_9_vs_In_OE_9_peak_1957	Solyc12g088940.2
6.21059	IP_OE_9_vs_In_OE_9_peak_1958	Solyc12g094400.2
6.26694	IP_OE_9_vs_In_OE_9_peak_1959	Solyc12g094620.2
4.28179	IP_OE_9_vs_In_OE_9_peak_1960	Solyc12g094650.2
4.86461	IP_OE_9_vs_In_OE_9_peak_1961	Solyc12g095750.2
5.66638	IP_OE_9_vs_In_OE_9_peak_1962	Solyc12g095800.2
4.38349	IP_OE_9_vs_In_OE_9_peak_1963	Solyc12g095980.2
4.03971	IP_OE_9_vs_In_OE_9_peak_1964	Solyc12g096210.2
4.87975	IP_OE_9_vs_In_OE_9_peak_1965	Solyc12g096350.2
4.93364	IP_OE_9_vs_In_OE_9_peak_1966	Solyc12g096670.2
4.58959	IP_OE_9_vs_In_OE_9_peak_1967	Solyc12g096860.2
5.32336	IP_OE_9_vs_In_OE_9_peak_1968	Solyc12g096870.1
4.61116	IP_OE_9_vs_In_OE_9_peak_1969	Solyc12g096880.2
5.64872	IP_OE_9_vs_In_OE_9_peak_1970	Solyc12g096910.1
5.76697	IP_OE_9_vs_In_OE_9_peak_1971	Solyc12g098430.2
5.32336	IP_OE_9_vs_In_OE_9_peak_1972	Solyc12g098480.1

3.62306	IP_OE_9_vs_In_OE_9_peak_1973	Solyc12g098620.2
6.97401	IP_OE_9_vs_In_OE_9_peak_1974	Solyc12g098750.1
7.54143	IP_OE_9_vs_In_OE_9_peak_1975	Solyc12g098910.2
5.23051	IP_OE_9_vs_In_OE_9_peak_1976	Solyc12g099460.1
6.59718	IP_OE_9_vs_In_OE_9_peak_1977	Solyc12g099780.2
6.847	IP_OE_9_vs_In_OE_9_peak_1978	Solyc12g100240.1
5.32336	IP_OE_9_vs_In_OE_9_peak_1979	
4.39179	IP_OE_9_vs_In_OE_9_peak_1980	
5.24036	IP_OE_9_vs_In_OE_9_peak_1981	
5.76697	IP_OE_9_vs_In_OE_9_peak_1982	
3.54891	IP_OE_9_vs_In_OE_9_peak_1983	
5.51094	IP_OE_9_vs_In_OE_9_peak_1984	
5.32336	IP_OE_9_vs_In_OE_9_peak_1985	
4.47344	IP_OE_9_vs_In_OE_9_peak_1986	
4.58959	IP_OE_9_vs_In_OE_9_peak_1987	
4.53556	IP_OE_9_vs_In_OE_9_peak_1988	
3.4235	IP_OE_9_vs_In_OE_9_peak_1989	
4.4085	IP_OE_9_vs_In_OE_9_peak_1990	
5.76697	IP_OE_9_vs_In_OE_9_peak_1991	
5.32336	IP_OE_9_vs_In_OE_9_peak_1992	
5.31039	IP_OE_9_vs_In_OE_9_peak_1993	
3.97521	IP_OE_9_vs_In_OE_9_peak_1994	
5.76697	IP_OE_9_vs_In_OE_9_peak_1995	
3.80389	IP_OE_9_vs_In_OE_9_peak_1996	
3.99252	IP_OE_9_vs_In_OE_9_peak_1997	
5.76697	IP_OE_9_vs_In_OE_9_peak_1998	
3.99041	IP_OE_9_vs_In_OE_9_peak_1999	
4.23654	IP_OE_9_vs_In_OE_9_peak_2000	
3.99252	IP_OE_9_vs_In_OE_9_peak_2001	
4.87975	IP_OE_9_vs_In_OE_9_peak_2002	
4.61116	IP_OE_9_vs_In_OE_9_peak_2003	
5.3638	IP_OE_9_vs_In_OE_9_peak_2004	
5.32336	IP_OE_9_vs_In_OE_9_peak_2005	
4.87975	IP_OE_9_vs_In_OE_9_peak_2006	
6.21059	IP_OE_9_vs_In_OE_9_peak_2007	
6.18486	IP_OE_9_vs_In_OE_9_peak_2008	
3.50679	IP_OE_9_vs_In_OE_9_peak_2009	
4.87975	IP_OE_9_vs_In_OE_9_peak_2010	
6.59718	IP_OE_9_vs_In_OE_9_peak_2011	
4.43613	IP_OE_9_vs_In_OE_9_peak_2012	
3.54891	IP_OE_9_vs_In_OE_9_peak_2013	
4.87975	IP_OE_9_vs_In_OE_9_peak_2014	
4.36697	IP_OE_9_vs_In_OE_9_peak_2015	
4.12324	IP_OE_9_vs_In_OE_9_peak_2016	
4.87975	IP_OE_9_vs_In_OE_9_peak_2017	
3.77541	IP_OE_9_vs_In_OE_9_peak_2018	
5.32336	IP_OE_9_vs_In_OE_9_peak_2019	

4.43613	IP_OE_9_vs_In_OE_9_peak_2020
4.87975	IP_OE_9_vs_In_OE_9_peak_2021
4.43613	IP_OE_9_vs_In_OE_9_peak_2022
4.32136	IP_OE_9_vs_In_OE_9_peak_2023
7.54143	IP_OE_9_vs_In_OE_9_peak_2024
4.5386	IP_OE_9_vs_In_OE_9_peak_2025
4.23654	IP_OE_9_vs_In_OE_9_peak_2026
6.94409	IP_OE_9_vs_In_OE_9_peak_2027
3.4288	IP_OE_9_vs_In_OE_9_peak_2028
2.60685	IP_OE_9_vs_In_OE_9_peak_2029
2.37192	IP_OE_9_vs_In_OE_9_peak_2030
2.61859	IP_OE_9_vs_In_OE_9_peak_2031
3.49579	IP_OE_9_vs_In_OE_9_peak_2032
1.88142	IP_OE_9_vs_In_OE_9_peak_2033
3.6256	IP_OE_9_vs_In_OE_9_peak_2034
6.21059	IP_OE_9_vs_In_OE_9_peak_2035
3.74052	IP_OE_9_vs_In_OE_9_peak_2036
1.94341	IP_OE_9_vs_In_OE_9_peak_2037
2.26407	IP_OE_9_vs_In_OE_9_peak_2038
2.37567	IP_OE_9_vs_In_OE_9_peak_2039
2.68059	IP_OE_9_vs_In_OE_9_peak_2040
3.56915	IP_OE_9_vs_In_OE_9_peak_2041
5.2897	IP_OE_9_vs_In_OE_9_peak_2042
2.60983	IP_OE_9_vs_In_OE_9_peak_2043
2.52328	IP_OE_9_vs_In_OE_9_peak_2044
2.0971	IP_OE_9_vs_In_OE_9_peak_2045
4.94053	IP_OE_9_vs_In_OE_9_peak_2046
2.12746	IP_OE_9_vs_In_OE_9_peak_2047
2.71435	IP_OE_9_vs_In_OE_9_peak_2048
4.86461	IP_OE_9_vs_In_OE_9_peak_2049
3.23564	IP_OE_9_vs_In_OE_9_peak_2050
4.80367	IP_OE_9_vs_In_OE_9_peak_2051
4.58959	IP_OE_9_vs_In_OE_9_peak_2052
5.02272	IP_OE_9_vs_In_OE_9_peak_2053
3.89359	IP_OE_9_vs_In_OE_9_peak_2054
2.67765	IP_OE_9_vs_In_OE_9_peak_2055
2.12509	IP_OE_9_vs_In_OE_9_peak_2056
3.07756	IP_OE_9_vs_In_OE_9_peak_2057
4.94506	IP_OE_9_vs_In_OE_9_peak_2058
2.59955	IP_OE_9_vs_In_OE_9_peak_2059
1.94834	IP_OE_9_vs_In_OE_9_peak_2060
3.8262	IP_OE_9_vs_In_OE_9_peak_2061
2.43208	IP_OE_9_vs_In_OE_9_peak_2062
2.66784	IP_OE_9_vs_In_OE_9_peak_2063
2.26578	IP_OE_9_vs_In_OE_9_peak_2064
3.1043	IP_OE_9_vs_In_OE_9_peak_2065
2.35636	IP_OE_9_vs_In_OE_9_peak_2066

2.15418	IP_OE_9_vs_In_OE_9_peak_2067
1.98488	IP_OE_9_vs_In_OE_9_peak_2068
2.21495	IP_OE_9_vs_In_OE_9_peak_2069
2.65792	IP_OE_9_vs_In_OE_9_peak_2070
2.19686	IP_OE_9_vs_In_OE_9_peak_2071
2.75278	IP_OE_9_vs_In_OE_9_peak_2072
2.05909	IP_OE_9_vs_In_OE_9_peak_2073
2.31681	IP_OE_9_vs_In_OE_9_peak_2074
2.34886	IP_OE_9_vs_In_OE_9_peak_2075
2.2247	IP_OE_9_vs_In_OE_9_peak_2076
2.98096	IP_OE_9_vs_In_OE_9_peak_2077
3.38811	IP_OE_9_vs_In_OE_9_peak_2078
2.27191	IP_OE_9_vs_In_OE_9_peak_2079
3.35159	IP_OE_9_vs_In_OE_9_peak_2080
2.44394	IP_OE_9_vs_In_OE_9_peak_2081
2.24087	IP_OE_9_vs_In_OE_9_peak_2082
1.94763	IP_OE_9_vs_In_OE_9_peak_2083
3.22412	IP_OE_9_vs_In_OE_9_peak_2084
2.91678	IP_OE_9_vs_In_OE_9_peak_2085
4.58959	IP_OE_9_vs_In_OE_9_peak_2086
4.53556	IP_OE_9_vs_In_OE_9_peak_2087
1.99467	IP_OE_9_vs_In_OE_9_peak_2088
7.54143	IP_OE_9_vs_In_OE_9_peak_2089
5.98963	IP_OE_9_vs_In_OE_9_peak_2090
4.62085	IP_OE_9_vs_In_OE_9_peak_2091
4.43613	IP_OE_9_vs_In_OE_9_peak_2092
7.09781	IP_OE_9_vs_In_OE_9_peak_2093
5.76697	IP_OE_9_vs_In_OE_9_peak_2094
5.62408	IP_OE_9_vs_In_OE_9_peak_2095
5.32336	IP_OE_9_vs_In_OE_9_peak_2096
4.87975	IP_OE_9_vs_In_OE_9_peak_2097
4.87975	IP_OE_9_vs_In_OE_9_peak_2098
5.76697	IP_OE_9_vs_In_OE_9_peak_2099
4.23654	IP_OE_9_vs_In_OE_9_peak_2100
4.9424	IP_OE_9_vs_In_OE_9_peak_2101
3.87192	IP_OE_9_vs_In_OE_9_peak_2102
3.46097	IP_OE_9_vs_In_OE_9_peak_2103
3.80389	IP_OE_9_vs_In_OE_9_peak_2104
3.99252	IP_OE_9_vs_In_OE_9_peak_2105
4.43613	IP_OE_9_vs_In_OE_9_peak_2106
5.32336	IP_OE_9_vs_In_OE_9_peak_2107
4.53556	IP_OE_9_vs_In_OE_9_peak_2108
3.80389	IP_OE_9_vs_In_OE_9_peak_2109
5.26999	IP_OE_9_vs_In_OE_9_peak_2110
3.89359	IP_OE_9_vs_In_OE_9_peak_2111
4.05384	IP_OE_9_vs_In_OE_9_peak_2112
4.43613	IP_OE_9_vs_In_OE_9_peak_2113

3.7888	IP_OE_9_vs_In_OE_9_peak_2114
3.56463	IP_OE_9_vs_In_OE_9_peak_2115
4.80578	IP_OE_9_vs_In_OE_9_peak_2116
4.94506	IP_OE_9_vs_In_OE_9_peak_2117
4.23654	IP_OE_9_vs_In_OE_9_peak_2118
3.99252	IP_OE_9_vs_In_OE_9_peak_2119
3.72786	IP_OE_9_vs_In_OE_9_peak_2120
4.53556	IP_OE_9_vs_In_OE_9_peak_2121
3.71092	IP_OE_9_vs_In_OE_9_peak_2122
4.87975	IP_OE_9_vs_In_OE_9_peak_2123
5.32336	IP_OE_9_vs_In_OE_9_peak_2124
5.70583	IP_OE_9_vs_In_OE_9_peak_2125
3.8835	IP_OE_9_vs_In_OE_9_peak_2126
3.71092	IP_OE_9_vs_In_OE_9_peak_2127
6.21059	IP_OE_9_vs_In_OE_9_peak_2128
3.71092	IP_OE_9_vs_In_OE_9_peak_2129
4.35624	IP_OE_9_vs_In_OE_9_peak_2130
6.21059	IP_OE_9_vs_In_OE_9_peak_2131
4.43613	IP_OE_9_vs_In_OE_9_peak_2132
4.87975	IP_OE_9_vs_In_OE_9_peak_2133
3.4235	IP_OE_9_vs_In_OE_9_peak_2134
5.57208	IP_OE_9_vs_In_OE_9_peak_2135
6.00177	IP_OE_9_vs_In_OE_9_peak_2136
3.04532	IP_OE_9_vs_In_OE_9_peak_2137
4.87975	IP_OE_9_vs_In_OE_9_peak_2138
5.76697	IP_OE_9_vs_In_OE_9_peak_2139
4.43613	IP_OE_9_vs_In_OE_9_peak_2140
4.87975	IP_OE_9_vs_In_OE_9_peak_2141
4.94506	IP_OE_9_vs_In_OE_9_peak_2142
3.70402	IP_OE_9_vs_In_OE_9_peak_2143
4.43613	IP_OE_9_vs_In_OE_9_peak_2144
5.23051	IP_OE_9_vs_In_OE_9_peak_2145
5.32336	IP_OE_9_vs_In_OE_9_peak_2146
5.32336	IP_OE_9_vs_In_OE_9_peak_2147
4.31816	IP_OE_9_vs_In_OE_9_peak_2148
4.38349	IP_OE_9_vs_In_OE_9_peak_2149
5.92864	IP_OE_9_vs_In_OE_9_peak_2150
4.33431	IP_OE_9_vs_In_OE_9_peak_2151
2.72968	IP_OE_9_vs_In_OE_9_peak_2152
3.43004	IP_OE_9_vs_In_OE_9_peak_2153
4.82183	IP_OE_9_vs_In_OE_9_peak_2154
4.87975	IP_OE_9_vs_In_OE_9_peak_2155
4.33048	IP_OE_9_vs_In_OE_9_peak_2156
3.80389	IP_OE_9_vs_In_OE_9_peak_2157
4.87975	IP_OE_9_vs_In_OE_9_peak_2158
6.21059	IP_OE_9_vs_In_OE_9_peak_2159
4.87975	IP_OE_9_vs_In_OE_9_peak_2160

4.01269	IP_OE_9_vs_In_OE_9_peak_2161
5.13397	IP_OE_9_vs_In_OE_9_peak_2162
4.83097	IP_OE_9_vs_In_OE_9_peak_2163
4.43613	IP_OE_9_vs_In_OE_9_peak_2164
3.80244	IP_OE_9_vs_In_OE_9_peak_2165
4.53556	IP_OE_9_vs_In_OE_9_peak_2166
2.54549	IP_OE_9_vs_In_OE_9_peak_2167
3.90088	IP_OE_9_vs_In_OE_9_peak_2168
4.12324	IP_OE_9_vs_In_OE_9_peak_2169
4.94053	IP_OE_9_vs_In_OE_9_peak_2170
2.36949	IP_OE_9_vs_In_OE_9_peak_2171
4.5386	IP_OE_9_vs_In_OE_9_peak_2172
4.43379	IP_OE_9_vs_In_OE_9_peak_2173
4.01269	IP_OE_9_vs_In_OE_9_peak_2174
1.91765	IP_OE_9_vs_In_OE_9_peak_2175
3.42056	IP_OE_9_vs_In_OE_9_peak_2176
4.33431	IP_OE_9_vs_In_OE_9_peak_2177
7.83415	IP_OE_9_vs_In_OE_9_peak_2178
4.58959	IP_OE_9_vs_In_OE_9_peak_2179
3.92288	IP_OE_9_vs_In_OE_9_peak_2180
4.43613	IP_OE_9_vs_In_OE_9_peak_2181
2.43511	IP_OE_9_vs_In_OE_9_peak_2182
4.87975	IP_OE_9_vs_In_OE_9_peak_2183
7.42385	IP_OE_9_vs_In_OE_9_peak_2184
4.87975	IP_OE_9_vs_In_OE_9_peak_2185
4.87975	IP_OE_9_vs_In_OE_9_peak_2186
4.87975	IP_OE_9_vs_In_OE_9_peak_2187
4.53556	IP_OE_9_vs_In_OE_9_peak_2188
6.19546	IP_OE_9_vs_In_OE_9_peak_2189
5.64872	IP_OE_9_vs_In_OE_9_peak_2190
3.4235	IP_OE_9_vs_In_OE_9_peak_2191
3.71092	IP_OE_9_vs_In_OE_9_peak_2192
6.72124	IP_OE_9_vs_In_OE_9_peak_2193
4.87975	IP_OE_9_vs_In_OE_9_peak_2194
6.00177	IP_OE_9_vs_In_OE_9_peak_2195
7.98504	IP_OE_9_vs_In_OE_9_peak_2196
3.00107	IP_OE_9_vs_In_OE_9_peak_2197
3.71092	IP_OE_9_vs_In_OE_9_peak_2198
5.14346	IP_OE_9_vs_In_OE_9_peak_2199
5.32336	IP_OE_9_vs_In_OE_9_peak_2200
5.76697	IP_OE_9_vs_In_OE_9_peak_2201
5.32336	IP_OE_9_vs_In_OE_9_peak_2202
4.87975	IP_OE_9_vs_In_OE_9_peak_2203
5.36021	IP_OE_9_vs_In_OE_9_peak_2204
3.99252	IP_OE_9_vs_In_OE_9_peak_2205
3.41809	IP_OE_9_vs_In_OE_9_peak_2206
4.75884	IP_OE_9_vs_In_OE_9_peak_2207

4.87975	IP_OE_9_vs_In_OE_9_peak_2208
5.19728	IP_OE_9_vs_In_OE_9_peak_2209
4.5386	IP_OE_9_vs_In_OE_9_peak_2210
3.54891	IP_OE_9_vs_In_OE_9_peak_2211
5.97624	IP_OE_9_vs_In_OE_9_peak_2212
4.58959	IP_OE_9_vs_In_OE_9_peak_2213
4.5386	IP_OE_9_vs_In_OE_9_peak_2214
3.54891	IP_OE_9_vs_In_OE_9_peak_2215
4.80453	IP_OE_9_vs_In_OE_9_peak_2216
4.94789	IP_OE_9_vs_In_OE_9_peak_2217
4.43613	IP_OE_9_vs_In_OE_9_peak_2218
4.20077	IP_OE_9_vs_In_OE_9_peak_2219
5.36021	IP_OE_9_vs_In_OE_9_peak_2220
5.5618	IP_OE_9_vs_In_OE_9_peak_2221
5.75293	IP_OE_9_vs_In_OE_9_peak_2222
3.99252	IP_OE_9_vs_In_OE_9_peak_2223
4.87975	IP_OE_9_vs_In_OE_9_peak_2224
6.06207	IP_OE_9_vs_In_OE_9_peak_2225
3.7807	IP_OE_9_vs_In_OE_9_peak_2226
4.12324	IP_OE_9_vs_In_OE_9_peak_2227
4.93869	IP_OE_9_vs_In_OE_9_peak_2228
4.38258	IP_OE_9_vs_In_OE_9_peak_2229
7.54143	IP_OE_9_vs_In_OE_9_peak_2230
4.43613	IP_OE_9_vs_In_OE_9_peak_2231
5.9676	IP_OE_9_vs_In_OE_9_peak_2232
4.68022	IP_OE_9_vs_In_OE_9_peak_2233
4.30852	IP_OE_9_vs_In_OE_9_peak_2234
4.06583	IP_OE_9_vs_In_OE_9_peak_2235
3.99252	IP_OE_9_vs_In_OE_9_peak_2236
4.43613	IP_OE_9_vs_In_OE_9_peak_2237
4.43613	IP_OE_9_vs_In_OE_9_peak_2238
3.8835	IP_OE_9_vs_In_OE_9_peak_2239
4.67165	IP_OE_9_vs_In_OE_9_peak_2240
3.4235	IP_OE_9_vs_In_OE_9_peak_2241
3.98416	IP_OE_9_vs_In_OE_9_peak_2242
3.99252	IP_OE_9_vs_In_OE_9_peak_2243
