# Supplementary material for: The tomato WRKY-B transcription factor modulates lateral branching by targeting BLIND, PIN4, and IAA15
Source: Hortic Res. 2024 Jul 11;11(9):uhae193. doi: 10.1093/hr/uhae193 (PMC11384121; doi:10.1093/hr/uhae193)
Supplement: Web_Material_uhae193 [file web_material_uhae193.zip › Figure s1-6.doc]

**Figure S1.**

**Figure S1. Phylogenetic analysis of WRKY proteins from tomato and *Arabidopsis***

Red circles indicate tomato and green triangles indicate *Arabidopsis*. Utilizing the MEGA6.0 software, the neighbour-joining phylogenetic tree was created.

**Figure S2.**

**Figure S2. Verification of the *wrky-b-c* mutants.**

(a) Generation of *wrky-b* mutants by utilizing CRISPR/Cas9 involves the use of two single-guide RNAs (sgRNA), referred to as target 1 and target 2 (illustrated with red arrows). The sgRNA target sequences are marked in red, while the protospacer-adjacent motif sequences are in bold. Black dashes represent the deletion sites, and the lengths of these sequence gaps are indicated within parentheses.

(b) Relative expression levels of *WRKY-B* in WT, *wrky-b* mutants and WRKY-B-GFP overexpressing transgenic lines. Error bars represent the SD of three biological replicates. Different letters indicate significant differences according to Student’s t test (**p <0.01, ***p <0.001)

**Figure S3.**

**Figure S3. Hierarchical clustering and heatmap analysis of the WRKY-B-regulated DEGs**

Hierarchical clustering and heatmap analysis of the WRKY-B-regulated DEGs in which involved in auxin signaling(a), auxin transport (b) and auxin-dependent lateral branching genes(c), respectively.

**Figure S4.**

**Figure S4. Genome browser view of WRKY-B ChIP-seq signals and the sequence of WRKY-B binding box at the target genes *BL*、*PIN4* and *IAA15*.** (a) Genome browser view of WRKY-B ChIP-seq signals of WRKY-B binding box at the target genes. The red vertical line represents the binding site. (b) The WRKY-B binding box-containing promoter sequence of *BL*, *PIN4*, and *IAA15* as DNA probes for EMSA. WRKY-B binding box sequences are highlighted in red.

**Figure S5.**

**Figure S5. Generation of *bl* and *pin4* mutants by CRISPR/Cas9**

CRISPR/Cas9 was used to edit *BL* and *PIN4* by utilizing two sgRNAs (target 1 and target 2 (indicated by red arrows)). The sgRNA targets and protospacer-adjacent motif sequences are highlighted in bold and red font, respectively. Insertions are shown in blue font, while deletions are denoted by dashes. The length of the sequence gap is displayed in parentheses.

**Figure S6.**

**Figure S6**. **Both *wrky-b* and *bl* mutants displayed the determinate growth habits.** The image (a) and (b) represent the *wrky-b* mutants and *bl* mutants, respectively.
