# Supplementary material for: The tomato WRKY-B transcription factor modulates lateral branching by targeting BLIND, PIN4, and IAA15
Source: Hortic Res. 2024 Jul 11;11(9):uhae193. doi: 10.1093/hr/uhae193 (PMC11384121; doi:10.1093/hr/uhae193)
Supplement: Web_Material_uhae193 [file web_material_uhae193.zip › table s1.doc]

**Table S1. Primers used in this study.**

| **Primers for gene amplification and vector construction** | |
| --- | --- |
| **Primers for targets detection** | |
| CR-*wrky-B*-F | ACAAAAGTGGGGTCTACATCTATTC |
| CR-*wrky-B*-R | CCATCTTCAAGATGATCAACTTCAC |
| CR-*bl*-F | ACTCTTGTCCATTTGTCTATATATC |
| CR-*bl*-R | CATTTCTTTCTGAACCCTAATTTAA |
| CR-*pin4-*F | AAATGGTAGCCTTGAATGGAGT |
| CR-*pin4*-R | AGAAAACTGTGCTCCGGGAG |
| **Primers for WRKY-B-GFP** | |
| *WRKY-B-GFP*-F | TCTCGAGCTTTCGCGAGCTCATGGGTGATGAACTAAGAGATTT |
| *WRKY-B-GFP*-R | CCCTTGCTCACCATGGATCCTGGTTCTTGTTTAAGATTAAACATAG |
| **Primers for electrophoretic mobility shift assay** | |
| Probe-*BL* | AAGTAAGTTAAAAATAGGTCTGTCCTTTTGACTTTGTAATTATAAATATA |
| mProbe-*BL* | AAGTAAGTTAAAAATAGGTCTGTCCAAAACTGAAAGTAATTATAAATATA |
| Probe-*PIN4* | GTTCTGACCGAAAGTTAAAATGAAAAGTCTAAATTACTATTTATAATATC |
| mProbe-*PIN4* | GTTCTGACCGAAAGTTAAAATGATTTACGATTCTTACTATTTATAATATC |
| Probe-*IAA15* | ATTGACCAGATAGCAGTTTTGATAGTCAACTAACTCAGCTGCTAAGATGT |
| mProbe-*IAA15* | ATTGACCAGATAGCAGTTTTGGCAAGTCCAGAACTCAGCTGCTAAGATGT |
| **Primers for LUC activity measure assay** | |
| pGreenII-*BL*-S | GTCGACGGTATCGATAAGCTTTACACTTATAATTATACATTATACAACCATTTTAC |
| pGreenII-*BL*-AS | CGCTCTAGAACTAGTGGATCCTTTTTCTTTTGTTAAAAACAC |
| pGreenII-*IAA15*-3-S | GTCGACGGTATCGATAAGCTTTTTTTCTTTTGTTAAAAACAC |
| pGreenII-*IAA15*-3-AS | CGCTCTAGAACTAGTGGATCCAAATTAAACTTCATCATTTTCACCCTC |
| pGreenII*-PIN4-*S | GTCGACGGTATCGATAAGCTT CAAAGACATTACATATTGCAAAAATAGATG |
| pGreenII-*PIN4*-AS | CGCTCTAGAACTAGTGGATCC AAGGTGTAAAATGTGGAAGATTTAT |
| **Primers for yeast one-hybrid** | |
| AD-TF-TYB-S | TGGAGGCCAGTGAATTCATGGGTGATGAACTAAGAGATTTGT |
| AD-TF-TYB-AS | GCCCACCCGGGTGGAATTCTCATGGTTCTTGTTTAAGATTAAACATAGAAG |
| pHIS2-*PIN*-1-S | GACTCACTATAGGGCGAATTC TTGGCAGAATTTTTTAATCTTTAAACATAGGA |
| pHIS2-*PIN*-1-AS | GCGTGAGCTCCCCGGGAATTC AAGAATCGTATTCATCTCGAGAGTT |
| pHIS2*-BL*-S | GACTCACTATAGGGCGAATTC TTATGACTGGACGCTATTAAAATCAAAT |
| pHIS2*-BL*-AS | GCGTGAGCTCCCCGGGAATTCTTTTTCTTTTGTTAAAAACACACTAAAAGTAT |
| pHIS2-*IAA15*-S | GACTCACTATAGGGCGAATTC TTCGTAATGATTTAGGGAATGAGATTACAA |
| pHIS2-*IAA15*-AS | GCGTGAGCTCCCCGGGAATTCCTAATTCTCTTACACGTATCAACAAACATT |
| **Primers for qRT-PCR** | |
| *WRKY-B*-F | GTAGCGTCGCAAGGATGTTG |
| *WRKY-B*-R | TGCTCAAAGCCTCATGGTTCT |
| *BRC1b*-F | ACAGCAAGATCAACACCGCT |
| *BRC1b*-R | GCTGGCCTTATCGAATCCCA |
| *BL*-F | TGGTCAATTATAGCGGCGCA |
| *BL*-R | TTGTTTTGTTGGGCTGCTGG |
| *IAA15*-F | CGAGACTATCTAAAAAGGGGG |
| *IAA15*-R | TGGTAACTGTCGTACATCTCC |
| *PIN4-*F | CCACCAAAGAAAAGAAAAACAAAAC |
| *PIN4*-R | TAATATCATAGCAACATACAGAGGA |
| *ACTIN2*-F | TTTGCCGCATGCCATTCTTC |
| *ACTIN2*-R | CGGACAATTTCCCGTTCAGC |

**Gene information used in this study.**

| **Description** | **Subject Id** | **Species** |
| --- | --- | --- |
| *SlARF1* | *Solyc01g103050* | *Solanum lycopersicum* |
| *SlARF2* | *Solyc03g118290* | *Solanum lycopersicum* |
| *SlARF3* | *Solyc02g077560* | *Solanum lycopersicum* |
| *SlARF4* | *Solyc11g069190* | *Solanum lycopersicum* |
| *SlARF5* | *Solyc04g081240* | *Solanum lycopersicum* |
| *SlARF6* | *Solyc12g006340* | *Solanum lycopersicum* |
| *SlARF7* | *Solyc07g042260* | *Solanum lycopersicum* |
| *SlARF8* | *Solyc02g037530* | *Solanum lycopersicum* |
| *SlARF9* | *Solyc08g082630* | *Solanum lycopersicum* |
| *SlARF10* | *Solyc11g069500* | *Solanum lycopersicum* |
| *SlARF11* | *Solyc12g042070* | *Solanum lycopersicum* |
| *SlARF12* | *Solyc08g008380* | *Solanum lycopersicum* |
| *SlARF13* | *Solyc05g056040* | *Solanum lycopersicum* |
| *SlARF16* | *Solyc09g007810* | *Solanum lycopersicum* |
| *SlARF18* | *Solyc01g096070* | *Solanum lycopersicum* |
| *SlARF19* | *Solyc07g016180* | *Solanum lycopersicum* |
| *SlPIN1* | *Solyc03g118740* | *Solanum lycopersicum* |
| *SlPIN9* | *Solyc10g078370* | *Solanum lycopersicum* |
| *SlPIN7* | *Solyc10g080880* | *Solanum lycopersicum* |
| *SlPIN4* | *Solyc05g008060* | *Solanum lycopersicum* |
| *SlPIN3* | *Solyc04g007690* | *Solanum lycopersicum* |
| *SlPIN8* | *Solyc02g087660* | *Solanum lycopersicum* |
| *SlPIN5* | *Solyc01g068410* | *Solanum lycopersicum* |
| *SlLAX1* | *Solyc09g014380* | *Solanum lycopersicum* |
| *SlLAX2* | *Solyc01g111310* | *Solanum lycopersicum* |
| *SlLAX3* | *Solyc11g013310* | *Solanum lycopersicum* |
| *SlLAX4* | *Solyc10g076790* | *Solanum lycopersicum* |
| *SlLAX5* | *Solyc10g055260* | *Solanum lycopersicum* |
| *SlIAA1* | *Solyc09g083280.3* | *Solanum lycopersicum* |
| *SlIAA3* | *Solyc09g065850.3* | *Solanum lycopersicum* |
| *SlIAA4* | *Solyc06g053840.3* | *Solanum lycopersicum* |
| *SlIAA8* | *Solyc12g007230.2* | *Solanum lycopersicum* |
| *SlIAA9* | *Solyc04g076850.3* | *Solanum lycopersicum* |
| *SlIAA11* | *Solyc12g096980.2* | *Solanum lycopersicum* |
| *SlIAA12* | *Solyc09g064530.3* | *Solanum lycopersicum* |
| *SlIAA14* | *Solyc09g083290.3* | *Solanum lycopersicum* |
| *SlIAA15* | *Solyc03g120390* | *Solanum lycopersicum* |
| *SlIAA16* | *Solyc01g097290.3* | *Solanum lycopersicum* |
| *SlIAA17* | *Solyc06g008590.3* | *Solanum lycopersicum* |
| *SlIAA19* | *Solyc03g120380* | *Solanum lycopersicum* |
| *SlIAA22* | *Solyc06g008580.3* | *Solanum lycopersicum* |
| *SlIAA23* | *Solyc04g054280.2* | *Solanum lycopersicum* |
| *SlIAA27* | *Solyc03g120500.3* | *Solanum lycopersicum* |
| *SlIAA29* | *Solyc08g021820.3* | *Solanum lycopersicum* |
| *SlIAA35* | *Solyc07g008020* | *Solanum lycopersicum* |
| *SlIAA36* | *Solyc06g066020.3* | *Solanum lycopersicum* |
| *WRKY-B* | *Solyc02g071130* | *Solanum lycopersicum* |
| *Blind (BL)* | *Solyc11g069030.1* | *Solanum lycopersicum* |
| *BZR1* | *Solyc02g063010* | *Solanum lycopersicum* |
| *D14* | [*Solyc04g077860.2*](https://solgenomics.net/feature/17806004/details) | *Solanum lycopersicum* |
| *ABCB19* | *Solyc02g087870.3.1* | *Solanum lycopersicum* |
| *SPL15* | *Solyc10g078700* | *Solanum lycopersicum* |
| *SPL13* | *Solyc05g015840* | *Solanum lycopersicum* |
| *SlGOB* | *Solyc07g062840* | *Solanum lycopersicum* |
| *LOG1* | *Solyc10g084150* | *Solanum lycopersicum* |
| *CKX2* | *Solyc01g088160* | *Solanum lycopersicum* |
| *RAX2* | *Solyc09g008250.3* | *Solanum lycopersicum* |
| *BRC1b* | *Solyc06g069240.2.1* | *Solanum lycopersicum* |
